# Ant Colonies Do Not Trade-Off Reproduction against Maintenance

**DOI:** 10.1371/journal.pone.0137969

**Published:** 2015-09-18

**Authors:** Boris H. Kramer, Alexandra Schrempf, Alexander Scheuerlein, Jürgen Heinze

**Affiliations:** 1 Max-Planck-Institute for Demographic Research, Konrad-Zuse-Str. 1, D-18055, Rostock, Germany; 2 Zoology / Evolutionary Biology lab, University of Regensburg, Universitätsstraße 31, D-93040, Regensburg, Germany; Universidade de São Paulo, Faculdade de Filosofia Ciências e Letras de Ribeirão Preto, BRAZIL

## Abstract

The question on how individuals allocate resources into maintenance and reproduction is one of the central questions in life history theory. Yet, resource allocation into maintenance on the organismic level can only be measured indirectly. This is different in a social insect colony, a “superorganism” where workers represent the soma and the queen the germ line of the colony. Here, we investigate whether trade-offs exist between maintenance and reproduction on two levels of biological organization, queens and colonies, by following single-queen colonies of the ant *Cardiocondyla obscurior* throughout the entire lifespan of the queen. Our results show that maintenance and reproduction are positively correlated on the colony level, and we confirm results of an earlier study that found no trade-off on the individual (queen) level. We attribute this unexpected outcome to the existence of a positive feedback loop where investment into maintenance (workers) increases the rate of resource acquisition under laboratory conditions. Even though food was provided ad libitum, variation in productivity among the colonies suggests that resources can only be utilized and invested into additional maintenance and reproduction by the colony if enough workers are available. The resulting relationship between per-capita and colony productivity in our study fits well with other studies conducted in the field, where decreasing per-capita productivity and the leveling off of colony productivity have been linked to density dependent effects due to competition among colonies. This suggests that the absence of trade-offs in our laboratory study might also be prevalent under natural conditions, leading to a positive association of maintenance, (= growth) and reproduction. In this respect, insect colonies resemble indeterminate growing organisms.

## Introduction

A central question in life history theory is how individuals allocate resources into maintenance and reproduction to maximize evolutionary fitness [1, 2]. As resources are typically finite, individuals must find a balance between investment in reproduction and maintenance. Allocation of resources into maintenance can be measured only indirectly, but the underlying trade-off causes a negative correlation between reproductive effort and lifespan, which is commonly observed throughout the animal kingdom [3].

Eusocial animals are special because their reproductives are unusually long-lived and highly fertile at the same time [4,5]. Workers have considerable shorter lifespan and forego reproduction [4,6] even though both castes develop from the same genome and live in the same environment [7]. Long queen lifespan has been connected to extrinsic mortality rate [8] or in case of the worker to lower costs due to efficiency gains by the division of labor [6,9].

In social insects selection acts simultaneously on the individual and the colony level [6,10]. Considering an insect colony as a “superorganism” [11], the colony workers are equivalent to the soma [sensu 12], whereas the queen takes the role as the germ line [13]. Investment into workers can therefore be budgeted as maintenance costs, whereas the production of sexual offspring represents investment into reproduction [6]. Hence, insect colonies offer the unique opportunity to directly quantify the life history traits maintenance (in this case equal to colony growth), reproduction, and lifespan, and therefore represent an ideal system to follow the shifting allocation of resources over the lifetime of the colony (e.g. [14]). In addition, it is also possible to measure individual-level trade-offs with reproduction as a direct measure, and lifespan as an indirect measure of maintenance., (e.g., [15,16]).

Here, we study investment into maintenance and reproduction both on the individual and the colony level in the ant *Cardiocondyla obscurior*. Building on an earlier study with experimentally manipulated colonies [15] we followed the natural growth of experimental colonies until the death of their queen and quantified the number of sexuals and workers produced. To our knowledge, this is the first study on perennial social insects (see [17] for annual bumble bees) with complete data of investment into offspring, as we counted actual brood size and composition, as well as the number of live individuals during the lifespan of a colony in close intervals. For analyses on the individual level we used queen lifetime reproductive effort as a measure of investment into reproduction and queen longevity as an indirect measure of investment into maintenance [6]. On the colony level we used the total number and the biomass of workers produced as a measure of investment into maintenance, and the number and biomass of sexuals (queens and males) as a measure of investment into reproduction.

Furthermore, we compared two distinct populations of *C*. *obscurior* (from Brazil and Japan) to study whether traits such as queen longevity and reproductive output are fixed within species. This is of special interest as colonies of the two populations differ in several aspects such as body size and queen/worker ratio [18, this study].

## Material and Methods

### Study species

*Cardiocondyla obscurior* (Wheeler, 1929) is a cosmopolitan tramp species, presumably of Southeast Asian origin, which has been spread by man across large parts of the tropics and subtropics (e.g.[19,20]). Colonies are facultatively polygynous, i.e., several queens may reproduce within the same colony [19]. Colonies were collected in Itabuna, Bahia Brazil and Okinawa, Japan, and since then reared as stock colonies in the laboratory (for details see [21]). Collecting of Brazilian colonies was allowed by Ministério do Meio Ambiente—MMA (Instituto Brasileiro do Meio Ambiente e dos Recursos Naturais Renováveis—IBAMA; Instituto Chico Mendes de Conservação da Biodiversidade—ICMBio) Número: 20324–1 and the collection of Japanese colonies did not necessitate a special permit. In both countries *Cardiocondyla obscurior* is not protected and an invasive tramp species. All animal treatment guidelines applicable to ants under international and German law have been followed.

### Experimental set-up

We set up 30 Brazilian and 60 Japanese experimental colonies from the stock colonies and followed them until the death of the queen. Colonies were kept in three-chambered nest boxes with a plaster floor in incubators under semi-natural temperature and light cycles (12h light 28°C / 12h dark 23°C). Colonies were fed two times a week with honey and pieces of insects (cockroaches and fruitflies).

Taking advantage of their natural mode of colony founding by budding (i.e. queens and workers form a satellite nest in the neighborhood of their natal nest) and regular inbreeding [22], we transferred a single queen pupa, a single pupa of a wingless male, and 20 young workers from the same stock colony into a new nest box. After the eclosion of the sexual pupae and their mating as adults the queen started egg laying. Henceforward, we counted the numbers of eggs, pupae and adult workers that emerged / developed in the experimental colonies twice per week. During the course of the experiment all dead workers were removed to quantify total investment in workers. Furthermore, we removed all sexual pupae to prevent a second queen from reproducing. Workers that died during the initial phase of the experiment were replaced with new workers from the stock-colony to maintain the initial number of twenty workers until the first pupae appeared in the colony. All brood remaining after the death of the queen was allowed to develop into pupae. In case the body of the queen remained intact after death, it was stored in 100% ethanol for subsequent size measurement (thorax length). Unfortunately, queen corpses were often dismembered by workers or disappeared and we were able to measure only 34 and 5 queens from Japan and Brazil, respectively.

### Dry weight

To estimate the biomass investment of the colonies on the basis of dry weight, we measured dry weight of the different castes from the Japanese population. Data on workers, queens, and males from the Brazilian population were already available [23], but confirmed by weighing 12 additional workers. From the Okinawa population, twelve workers, wingless males, and fertile queens were dried for 24 h at 65°C and individually weighed three times to the nearest 0.1 μg with a fine scale (Sartorius Micro SC2).

### Statistical Analysis

We performed CoxPH models to test for differences between queen lifespan as well as the onset of sexual reproduction between the Japanese and the Brazilian populations. We found that the measures we took for maintenance and reproduction where highly correlated both on the individual level as well as the colony level. We therefore performed Principal component (PCA) and tree analysis (recursive partitioning [24]) to identify the single measure with the highest explanatory power. We performed linear models to characterize existing trade-offs between factors characterizing maintenance and reproduction both on the individual level and the colony level. We further used ANCOVAs to test for subpopulation differences in the trade-off analysis and Wilcoxon rank sum tests to quantify differences between the populations with respect to egg-laying rate, differences in thorax length, the number of sexuals and workers produced, and sex ratio. We further prepared boxplots to visualize colony level and per-capita productivity as well as the egg-laying rates of the two populations. Several variables were log-transformed as indicated in the text to achieve normality and homoscedasticity.

All statistical analyses were performed using the package survival [25] and tree [26] within the R-statistical software [27].

## Results

### 1) Population comparisons

Queen survival was identical for both populations (CoxPH model: n = 84, events (i.e. uncensored deaths) = 71, coef = -0.115, p = 0.681), but Japanese queens had a lower daily egg-laying rate (the sum of all counted eggs during observations divided by queen lifespan in days) than Brazilian queens (Wilcoxon rank sum test: W = 485, p = 0.0157; mean Japan = 1.811, mean Brazil = 2.472). Despite of this, there was no significant difference in the total number of sexuals produced (Wilcoxon rank sum test: W = 597.5, p-value = 0.1945; mean Japan = 21.393, mean Brazil = 32.5), sex ratio (queens/(queens + males)) (Wilcoxon rank sum test: W = 577.5, p = 0.523; mean Japan = 0.700, mean Brazil = 0.748), the mean number of workers throughout the colony life cycle (Wilcoxon rank sum test: W = 552, p-value = 0.080; mean Japan = 20.538, mean Brazil = 25.932), the maximum number of workers in the experimental colonies (Wilcoxon rank sum test: W = 652, p-value = 0.447; mean Japan = 35.071, mean Brazil = 46.923), and the total number of workers produced throughout the experiment (Wilcoxon rank sum test: W = 889.5, p = 0.2445; mean Japan = 80.338, mean Brazil = 71.231).

We also did not find a significant difference in the onset of sexual reproduction between the two populations (CoxPH model: first sexual pupa (male or queen pupae) n = 77, events = 77, coef = -0.432, p = 0.0895), but the onset of queen production was on average 8 days earlier in the Japanese population (CoxPH model: first queen pupa, n = 73, events = 73, coef = -0.596, p = 0.026) while no differences appear in the production of males (CoxPH model: first male pupa, n = 69, events = 69, coef = -0.042, p = 0.872). On average the colonies started to produce their first sexuals after 70–78 days without a preference for male or queen production.

Contrary to our expectation, queen lifespan was independent of body size (linear model Brazil: intercept: 553.8, p = 0.212; slope: 665.0, p = 0.332; df = 3; r² = 0.308; Japan: intercept: 69.49, p = 0.705; slope: 98.33, p = 0.726; df = 32; r² = 0.003). Similarly, the number of eggs laid by a queen was independent of her body size (linear model Brazil: intercept: 498.6, p = 0.826; slope: -443.7, p = 0.905; df = 3; r² = 0.006; Japan: intercept: -22.76, p = 0.951; slope: 260.83, p = 0.644; df = 32; r² = 0.007).

### 2) Individual level trade-off

**Fig 1:**
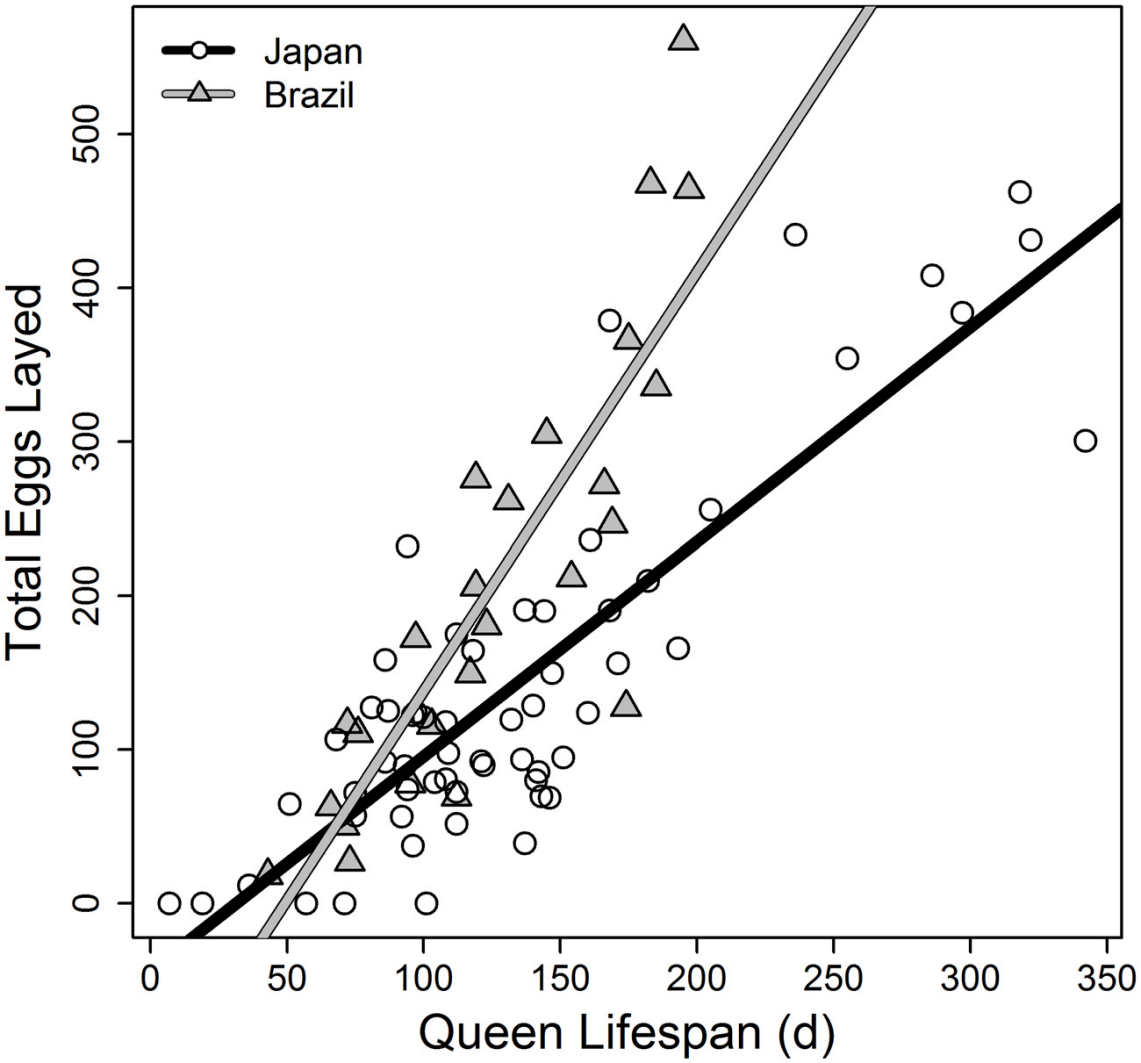
Individual level trade-off for queens. Correlation between total number of eggs laid and queen lifespan in Japanese (circles) and Brazilian (triangles) queens in single-queen colonies of the ant *Cardiocondyla obscurior*. The lines represent linear model fits for each subpopulation. Fecundity and lifespan are positively correlated.

All measures of reproduction were highly correlated, as tested by PCA and tree analysis: Queens, which laid more eggs, produced more workers and sexuals and had a higher number of eggs in the colony at any time. We therefore chose the total number of eggs laid during the lifespan of a colony as a measure of reproductive effort. There was a significant positive association between reproductive effort and queen lifespan in both populations (Fig 1), with the Brazilian queens achieving a certain reproductive effort earlier than the Japanese queens (linear models: Japan: intercept: -43.64, p = 0.015, slope: 1.39, p < 0.001, df = 57; r² = 0.72; Brazil: intercept: -134.03, p = 0.008, slope: 2.72, p < 0.001, df = 23; r² = 0.73). ANCOVA with queen lifespan as the covariate, population as explanatory variable, and the total number of eggs laid as the response variable showed a significant interaction term (queen lifespan × population¸ *F*2,81 = P<0.001) indicating different slopes among Japanese and Brazilian queens with respect to the association of lifespan and the total number of eggs laid by the queen (Fig 1).

**Fig 2:**
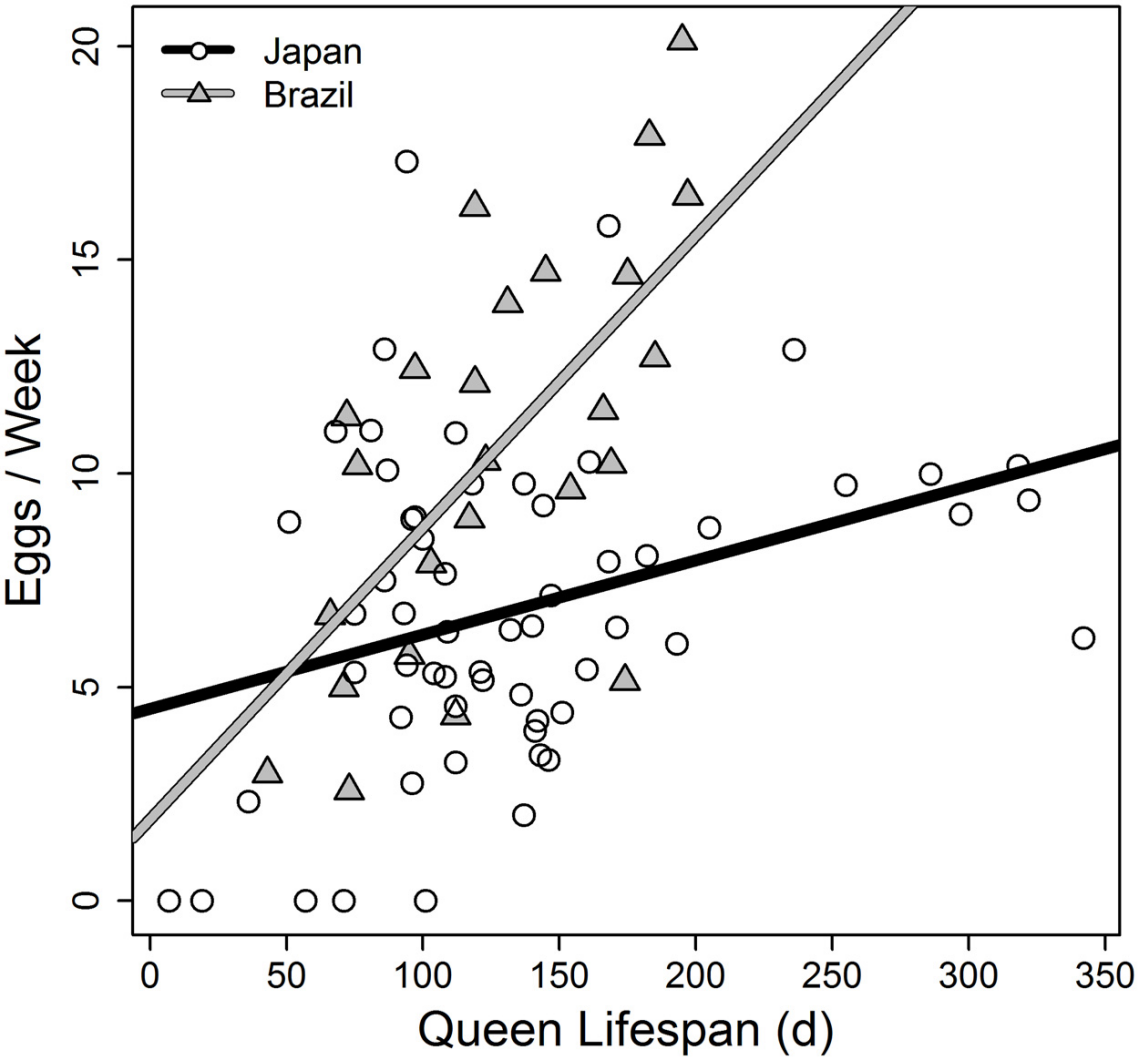
Individual level trade-off for queens. Association between egg laying rate and lifespan in Japanese (circles) and Brazilian (triangles) queens in single-queen colonies of the ant *Cardiocondyla obscurior*. The lines represent linear model fits for each subpopulation. Egg laying rate and lifespan are positively correlated.

Most importantly, queens that lived longer also had a higher weekly egg-laying rate (linear models: Japan: intercept: 4.501, p < 0.001, slope: 0.017, p = 0.00996, df = 57; r² = 0.11; Brazil: intercept: 1.92, p = 0.388, slope: 0.068, p < 0.001, df = 23; r² = 0.43; Fig 2). Again, Brazilian queens achieved a high egg-laying rate at earlier ages than Japanese queens.

**Fig 3:**
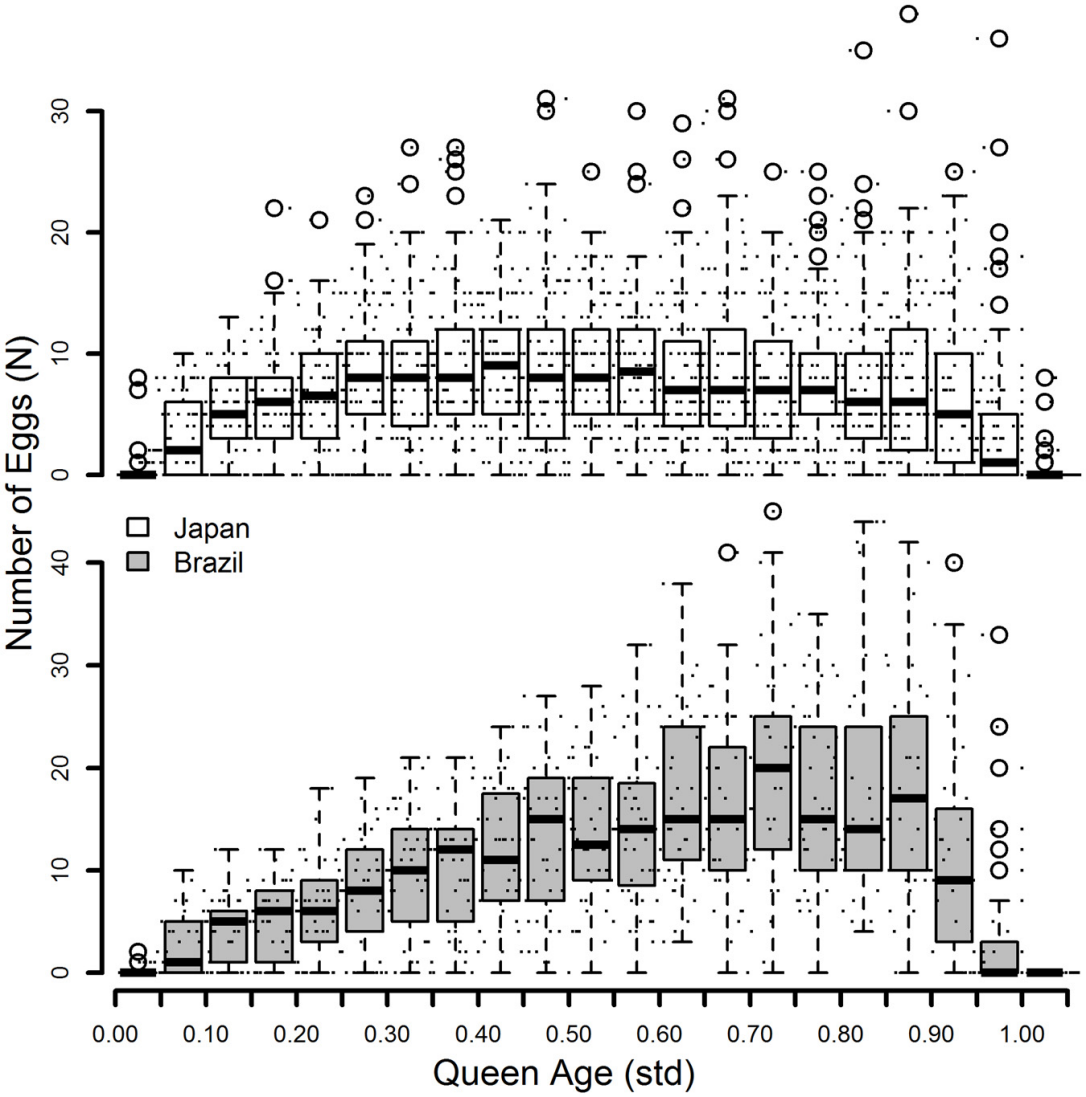
Number of eggs in the experimental colonies. Change in the number of eggs laid within 5% fractions of normalized lifespan from birth to death of queens of the ant *Cardiocondyla obscurior* (0 = birth, 1 = death). Small dots indicate the original data on which the boxplots where created, the circles represent outliers. Japanese colonies shown above, Brazilian colonies below.

While we found support for an increase in weekly egg-laying rate with colony age in the Brazilian population (see [15]), egg-laying rate appeared to stagnate after an initial increase in Japanese queens. In both populations, egg-laying rate decreased shortly before the queen’s death (Fig 3).

### 3) Trade-offs at the colony-level

Following Kramer and Schaible [6], we used the log of the total number of workers produced during the lifetime of a colony as a measure of colony maintenance. Similar to the individual level, there was a positive association between maintenance (total number of workers produced) and reproductive effort (total number of sexuals (males & queens) produced) (Linear models on log transformed data, Japan (Brazil): intercept: -3.231 (-2.176), slope: 1.271 (1.203), r² = 0.93, df = 81, p < 0.001). Brazilian colonies produced a given number of sexuals with fewer workers than Japanese colonies (Welch two sample t-test: t = -2.17, df = 43.66, p = 0.03, mean Japan (Brazil): 0.17 (0.3) sexuals/workers). Onset of the production of sexuals was identical between Brazilian and Japanese colonies (Japan: 67.7 ± 13.8 days; Brazil: 73.3 ± 20.2 days), independent of the number of workers in the colonies.

**Fig 4:**
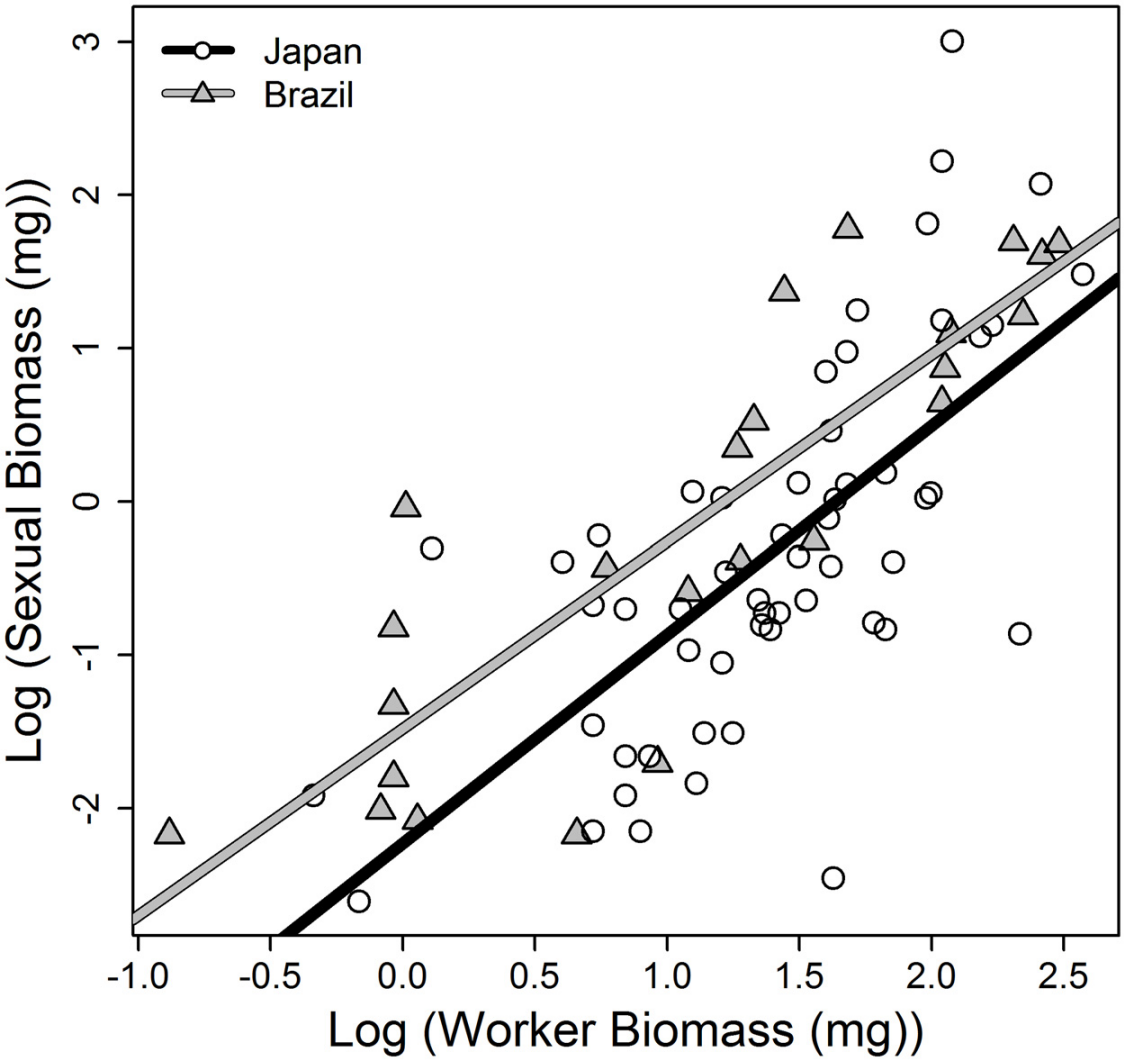
Colony level trade-off. Association between biomass investment into maintenance (log-transformed worker biomass) and reproduction (log-transformed sexual biomass) in single-queen colonies of the ant *Cardiocondyla obscurior* from two populations. The lines represent linear model fits for each subpopulation.

When comparing investments into biomass, thereby accounting for the different masses of queens, workers, and males, the results were similar: there was no trade-off between maintenance and reproduction, in the contrary, both traits are positively correlated (Linear models on log transformed data, Japan (Brazil): intercept: -2.231 (-1.481), slope: 1.362 (1.219), r² = 0.581, df = 75, p < 0.001; Fig 4). Colonies with a higher biomass of workers also invested more biomass in the production of sexuals. However, the Brazilian colonies seemed to invest more resources into the production of sexual biomass than the Japanese colonies but the result was not significant (Welch two sample t-test comparing biomass investment into sexuals relative to investment into workers: T = -1.183, df = 60.069, p = 0.062, mean Japan (Brazil): 0.28 (0.37)).

**Fig 5:**
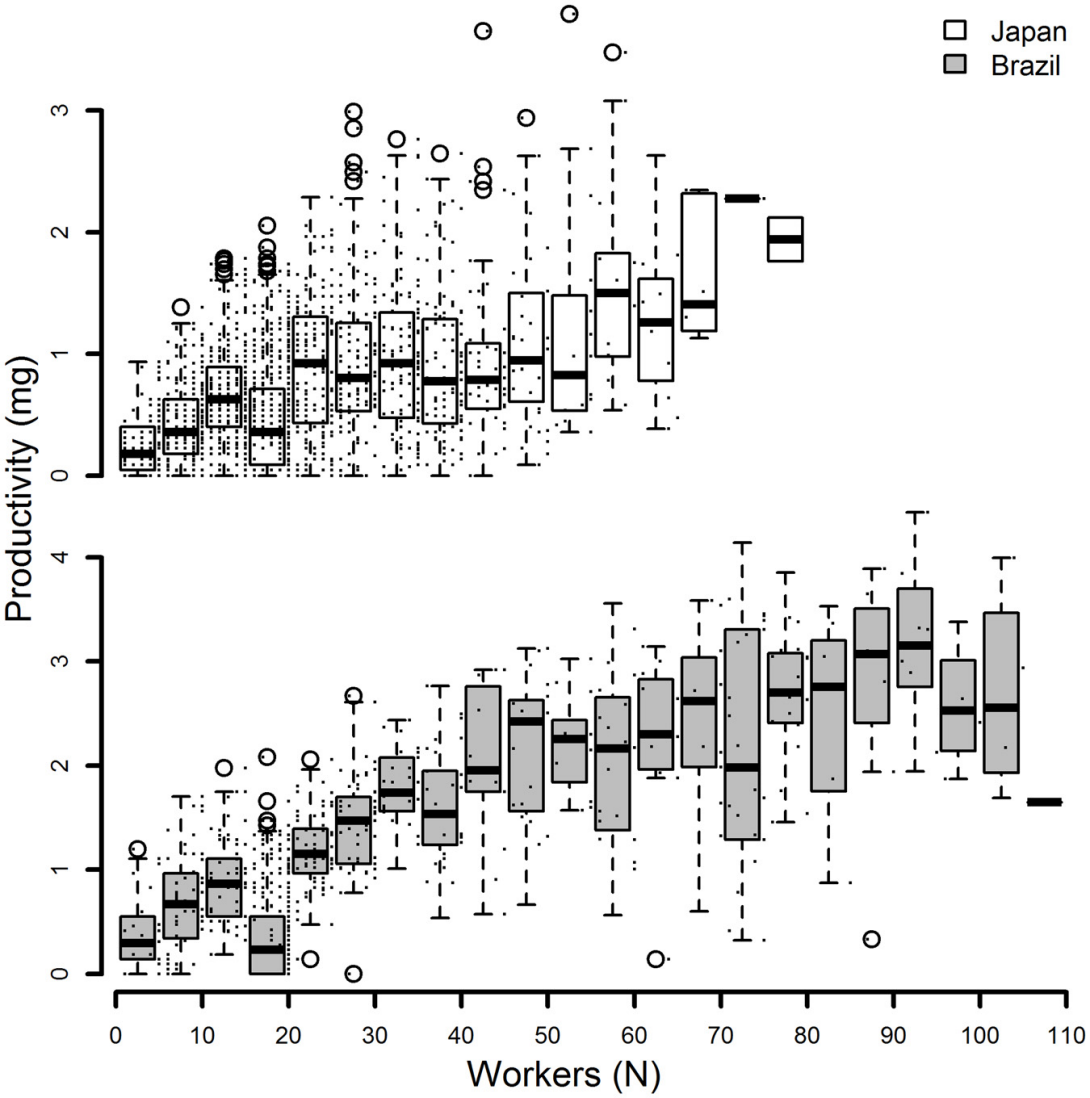
Colony level productivity. Colony productivity in mg biomass across different colony sizes of the ant *Cardiocondyla obscurior* expressed as the number of workers in the colony. Small dots indicate the original data on which the boxplots where created, the circles represent outliers. Japanese colonies shown above, Brazilian colonies below.

**Fig 6:**
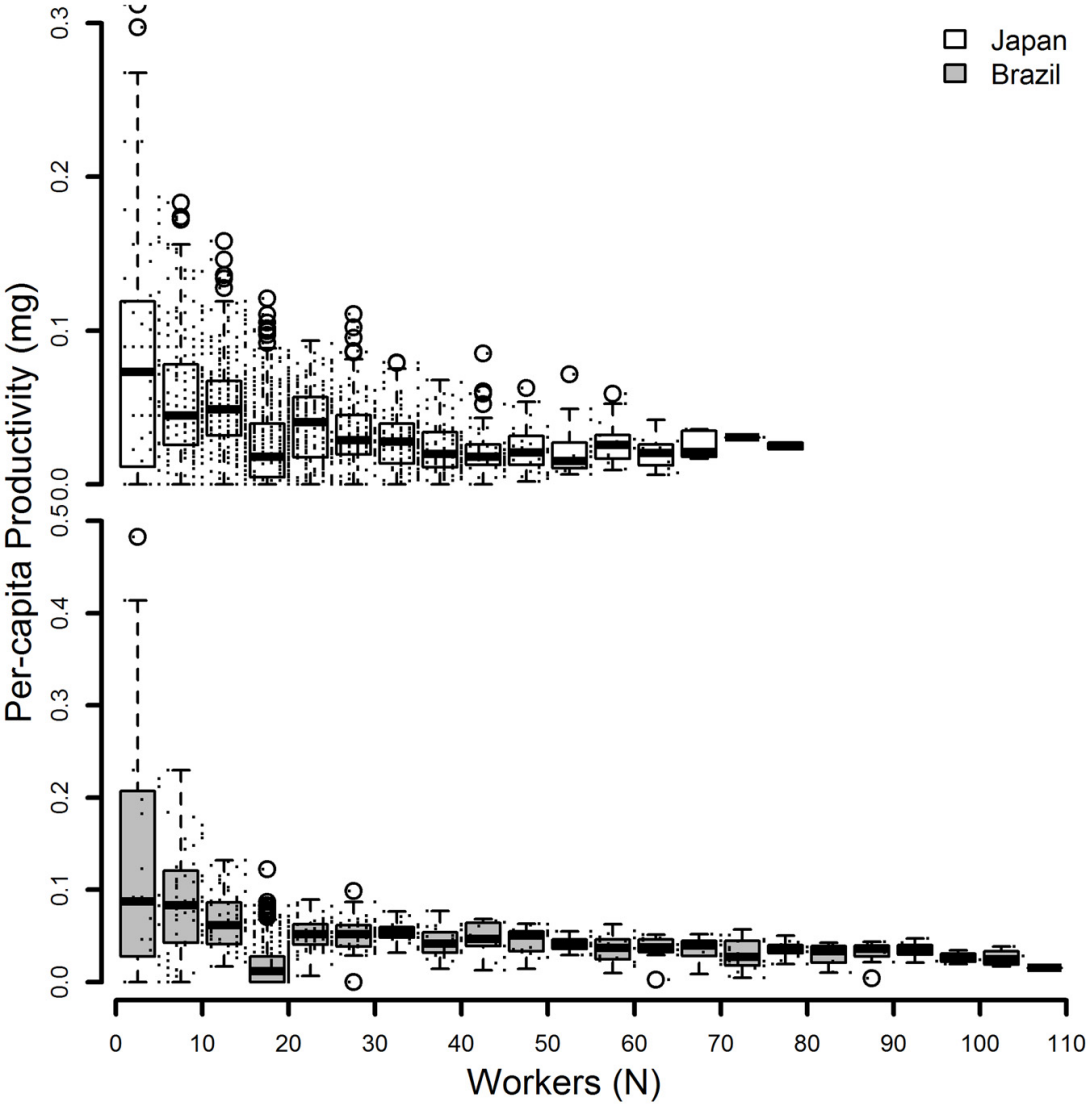
Per-capita productivity. Colony productivity in mg biomass divided by the number of workers in the colony across different colony sizes of the ant *Cardiocondyla obscurior*, expressed as the number of workers on the x- axis. Small dots indicate the original data on which the boxplots where created, the circles represent outliers. Japanese colonies shown above, Brazilian colonies below.

Figs 5 & 6 show colony, and per-capita productivity respectively. Colony productivity leveled off for both populations (Fig 5) and per-capita productivity showed a decline with increasing colony size as well as a decrease in variance with increasing colony size (Fig 6), patterns typical for social insect colonies.

## Discussion

Our comparison of the investment of colonies of *Cardiocondyla obscurior* into sexuals and workers allows assessing whether a trade-offs exists between maintenance and reproduction on the level of the individual queen and the level of the whole colony. Our data clearly show that maintenance and reproduction are positively correlated on both levels and that—at least under laboratory conditions with food provided ad libitum—a trade-off is not visible. This confirms the results of a similar study on the related species *C*. *kagutsuchi*, which, however, did only record the number of workers present at the time of the queen’s death instead of the total number of workers produced [16]. Furthermore, our results also do not substantiate an individual-level trade-off between maintenance and reproduction for individual queens and thus corroborates earlier studies [15,16].

While the absence of a trade-off on the level of the individual queen might be explained by the costs of reproduction being in part borne by the workers, the absence of the trade-off on the colony level is unexpected. Kramer and Schaible [5] hypothesized that trade-offs in insect colonies could be moved to the colony level. Hence, colonies with only a finite amount of resources available face the decision on how to allocate these limited resources between maintenance and reproduction—i.e., the investment into workers or sexuals. Trade-offs are hard to detect if resource levels vary among individuals or, in our case, colonies [28]. The production of new workers by the queen increases colony size as well as the number of individuals that can retrieve resources from the environment [29]. These additional resources can then be utilized by the colony to further increase colony growth or reproduction, setting off a self-reinforcing feedback loop that continually leads to an increase in resources available for the colony while defying the trade-off on the colony level. When resources are provided ad libitum and neither predation nor density-dependent effects exist—as in laboratory conditions—investment in reproduction and maintenance may be positively correlated (Fig 4). For example, similar “benign” conditions have been suggested to explain the absence of a trade-off between reproduction and longevity in zoo animals [30].

Several studies revealed that existing trade-offs may not be detectable by an observer if individuals differ in their capability of resource acquisition (e.g. [31]). In our study, some colonies could not make full use of the surplus resources available despite the ad libitum feeding- regime in the laboratory, probably because their workforce was insufficient. This invariably led to strong heterogeneity among colonies in the level of resources that were available. In addition, variations among queens with respect to egg-laying / resource-level reaction norms might have been exacerbated by the above mentioned positive feedback loop. Consequently, lifespan and reproductive output of queens were extremely variable among colonies, even if queens only differed a little in their ability to utilize continually increasing resources.

In most insect colonies studied so far productivity levels off with increasing colony size [32,33] an effect that we also found in our study (Fig 5). This translates into a decrease in per-capita productivity (Fig 6), also known as ‘Michener’s paradox’ [34,], which is accompanied by a typical decrease in the variance of per-capita productivity with increasing colony size [32,35,36]. It is striking that we found a similar pattern also in our ad libitum fed colonies. The decreasing variance in per-capita productivity hasso far has been understood as being caused by an increasing chance to find and retrieve food items from the environment as forager numbers increase[32]. As the colony continues to grow negative density dependent effects caused by increasing foraging distances, and a higher chance of competition with other conspecific colonies should ultimately lead to a leveling off of colony productivity. However, in our study we find the same pattern in the laboratory without resource limitation [32,36,37]. Hence, colony-level productivity does not seem to be limited by the amount of resources available (which can be ruled out in our study) but rather by worker number, which determines the resources that can be used by the colony. The high variance in productivity at small colony sizes in the absence of resource limitation points to organizational constraints within the workforce of the respective colonies. However, as long as the amount of resources that can be efficiently utilized by the colony increases (despite the above-mentioned diminishing returns with increasing colony size), longer lifespan will be correlated with increased reproductive output. Therefore, the positive relationship of maintenance and reproduction may not only be a result of ad libitum feeding regimes in the laboratory, but may be also found under natural conditions.

To be able to follow the lifespan and egg-laying of individual queens, we had to remove sexual brood. Under natural conditions, sexual offspring would eventually stay in the nest and contribute to offspring production, which would generate potentially immortal colonies, where trade-offs are hard to measure. Under these conditions, the lower productivity of some queens may be compensated by the performance of more productive offspring queens, and it remains to be investigated whether trade-offs on the colony level change when colonies are allowed to form polygynous colonies. In any case, it was striking that all colonies initiated sexual production at a certain time point regardless of colony size. This contrasts with the expectation of an optimal threshold colony size. However, *C*. *obscurior* and other species with facultative polygyny, mating in the nest, and the ability to form new colonies by fragmentation (budding) appear to be adapted to unpredictable and disturbed habitats [38]. Thus, as in other r-strategists, there is a strong selective advantage to produce sexual offspring early and independent of worker number, and to quickly increase colony size after a colonization event [39,40]. At least in laboratory colonies of *C*. *obscurior*, queen number is much more important for colony growth than worker number, as more queens contribute to a higher egg number [41].

Although egg-laying rate and lifespan were positively correlated in both populations, the strength of the association differed. Hence, the association between these two life history traits appears not to be fixed across populations but seems to be adaptable to specific environmental parameters such as the size and stability of natural nest cavities etc. In the field, colonies nest in in rolled leaves of lemon trees and aborted fruits of coconuts in Brazil (Heinze et al. 2006), while they can be found in bark cavities in coral trees in Japan. Queen number under natural conditions is larger in Japanese colonies (Schrader et al. 2014), but it is unclear whether this is a result of relaxed selection on competitive abilities and a lower egg-laying rate in Japanese queens, or whether a lower egg-laying rate promotes more queens staying together. Japanese queens are larger than Brazilian queens (Schrader et al. 2014), and, generally, lifespan and fecundity seem to be independent of body size in *C*. *obscurior*, as confirmed in the intra-population comparisons. This matches observations in some animals, but not in others [42–46].

In both populations, egg-laying rate increased after egg-laying started, but then either remained on a plateau (Japan population) or further increased with age (Brazil population) and decreased sharply short before the queen’s death, regardless of her age (Fig 3). This is different to what has been shown in solitary insects [47,48] or captive mammal and bird species, in which fertility increases for some time after sexual maturity, but then decreases at ages where more than 60% of the initial cohort are still alive [49]. Constant or even increasing fertility over age is rare and can only be found in organisms with infinite growth, such as some species of trees, fish, mollusks, crocodiles, and turtles [50]). Common to all these organisms is a prolonged growth period where large size is associated with higher fitness, such as a reduction in mortality risk at large sizes, or an increase in the capability for acquiring resources. When a *C*. *obscurior* colony is treated as a superorganism their life history stunningly resembles that of the above-mentioned organisms with indeterminate growth. Interestingly, the observed decline immediately before death in our study stands in contrast to what has been shown previously [15] and might be attributed to early costs of reproduction, which did not exist in the former study in which colony size was restricted. However, since the decline in egg-laying is immediately followed by the death of the queen, it may also be a sign of physiological senescence as a result of biochemical deterioration of the organism. Future studies under strong resource limitation could show how colonies allocate finite resources into reproduction and maintenance, and whether this causes a similar trade-off in individual queens as well as colonies.

## Supporting Information

S1 File SI-file contains all data used for the statistical tests in the results section as well as all the data used for plotting the figures and is ordered according to the results section. (DOCX)
