# Supplementary material for: Ant Colonies Do Not Trade-Off Reproduction against Maintenance
Source: PLoS One. 2015 Sep 18;10(9):e0137969. doi: 10.1371/journal.pone.0137969 (PMC4575186; doi:10.1371/journal.pone.0137969)
Supplement: S1 File — (DOCX) [file pone.0137969.s001.docx]

**Supplememtal material**:

data used for all analysis; ordered following the results section of the publication.

**1)Population comparisons**

-queen survival

qls censored japBra

[1,] 100 1 1

[2,] 174 1 2

[3,] 147 1 1

[4,] 197 0 2

[5,] 297 1 1

[6,] 95 1 2

[7,] 236 1 1

[8,] 175 1 2

[9,] 183 1 2

[10,] 145 1 2

[11,] 112 1 1

[12,] 185 0 2

[13,] 112 1 1

[14,] 119 1 2

[15,] 123 1 2

[16,] 81 1 1

[17,] 195 1 2

[18,] NA 1 2

[19,] 182 1 1

[20,] 112 1 2

[21,] 342 1 1

[22,] NA 1 2

[23,] 75 1 1

[24,] 73 0 2

[25,] 322 1 1

[26,] 169 0 2

[27,] 286 1 1

[28,] 131 1 2

[29,] 318 1 1

[30,] 66 1 2

[31,] 137 1 1

[32,] 132 1 1

[33,] NA 1 2

[34,] 71 0 1

[35,] 97 1 2

[36,] 101 0 1

[37,] NA 1 2

[38,] 109 1 1

[39,] 76 0 2

[40,] 108 1 1

[41,] 71 1 2

[42,] 97 1 1

[43,] 72 0 2

[44,] 96 1 1

[45,] 141 0 1

[46,] 93 1 1

[47,] 104 1 1

[48,] 86 1 1

[49,] 86 1 1

[50,] 205 1 1

[51,] 168 0 1

[52,] 36 1 1

[53,] 75 1 1

[54,] 43 1 2

[55,] 144 1 1

[56,] 94 1 1

[57,] 94 1 1

[58,] 121 1 1

[59,] 122 1 1

[60,] 171 1 1

[61,] 87 1 1

[62,] 146 1 1

[63,] 118 1 1

[64,] 255 1 1

[65,] 117 1 2

[66,] 136 1 1

[67,] 108 0 1

[68,] 160 0 1

[69,] 193 1 1

[70,] 143 1 1

[71,] 140 1 1

[72,] 154 1 2

[73,] 142 1 1

[74,] 112 1 1

[75,] 19 1 1

[76,] 137 1 1

[77,] 161 1 1

[78,] 68 1 1

[79,] 119 0 2

[80,] 92 1 1

[81,] 57 1 1

[82,] 96 1 1

[83,] 151 1 1

[84,] 7 1 1

[85,] 51 1 1

[86,] 103 1 2

[87,] 168 1 1

[88,] 166 1 2

-egg laying rate

eggsPerDay japBra

[1,] 1.9108911 1

[2,] 2.4739884 1

[3,] 1.2845528 1

[4,] 1.5526316 1

[5,] 2.3966942 1

[6,] 1.4071429 1

[7,] 2.3913043 1

[8,] 1.3913043 1

[9,] 2.2678571 1

[10,] 1.9256757 1

[11,] 2.5167785 1

[12,] 0.7321429 1

[13,] 2.5178571 1

[14,] 2.0987654 1

[15,] 1.8060606 1

[16,] 1.2352941 1

[17,] 2.4503106 1

[18,] 2.7353952 1

[19,] 2.9192308 1

[20,] 2.6788321 1

[21,] 1.5939850 1

[22,] 0.5211268 1

[23,] 1.5392157 1

[24,] 1.8679245 1

[25,] 2.5384615 1

[26,] 2.7582418 1

[27,] 0.9579832 1

[28,] 1.0487805 1

[29,] 1.5047619 1

[30,] 2.9135802 1

[31,] 1.5061728 1

[32,] 2.2000000 1

[33,] 4.4161491 1

[34,] 0.7709924 1

[35,] 2.7000000 1

[36,] 3.9473684 1

[37,] 1.2808989 1

[38,] 1.3000000 1

[39,] 1.4715447 1

[40,] 1.9580838 1

[41,] 2.3181818 1

[42,] 0.6338028 1

[43,] 2.8487395 1

[44,] 1.1212121 1

[45,] 1.5068493 1

[46,] 1.5531915 1

[47,] 0.8057554 1

[48,] 1.0935252 1

[49,] 1.3097345 1

[50,] 0.5597015 1

[51,] 2.6855346 1

[52,] 1.2282609 1

[53,] 1.0175439 1

[54,] 0.7684211 1

[55,] 1.2770270 1

[56,] 1.1800000 1

[57,] 1.0555556 2

[58,] 1.1415929 2

[59,] 1.2266667 2

[60,] 0.3720930 2

[61,] 2.5299145 2

[62,] 2.4527027 2

[63,] 2.7451923 2

[64,] 1.0212766 2

[65,] 3.0679012 2

[66,] 4.1858407 2

[67,] 1.4788732 2

[68,] 3.8407643 2

[69,] 4.8804348 2

[70,] 3.9590164 2

[71,] 2.9581152 2

[72,] 2.9130435 2

[73,] 2.4933333 2

[74,] 2.0000000 2

[75,] 4.7754011 2

[76,] 0.5614035 2

[77,] 2.3537736 2

[78,] 3.0809249 2

[79,] 1.7538462 2

[80,] 2.8800000 2

[81,] 2.2033898 2

[82,] 2.3304348 2

- total number of sexuals produced

sexuals japBra

[1,] 15 1

[2,] 39 1

[3,] 0 1

[4,] 9 1

[5,] 87 1

[6,] 17 1

[7,] 13 1

[8,] 8 1

[9,] 22 1

[10,] 33 1

[11,] 131 1

[12,] 4 1

[13,] 13 1

[14,] 16 1

[15,] 8 1

[16,] 12 1

[17,] 50 1

[18,] 50 1

[19,] 262 1

[20,] 8 1

[21,] 16 1

[22,] 2 1

[23,] 0 1

[24,] 2 1

[25,] 49 1

[26,] 7 1

[27,] 3 1

[28,] 3 1

[29,] 8 1

[30,] 16 1

[31,] 8 1

[32,] 10 1

[33,] 69 1

[34,] 6 1

[35,] 9 1

[36,] 16 1

[37,] 8 1

[38,] 7 1

[39,] 10 1

[40,] 2 1

[41,] 17 1

[42,] 2 1

[43,] 19 1

[44,] 3 1

[45,] 9 1

[46,] 6 1

[47,] 3 1

[48,] 8 1

[49,] 11 1

[50,] 1 1

[51,] 46 1

[52,] 3 1

[53,] 10 1

[54,] 2 1

[55,] 10 1

[56,] 0 1

[57,] 4 2

[58,] 25 2

[59,] 2 2

[60,] 8 2

[61,] 43 2

[62,] 13 2

[63,] 37 2

[64,] 3 2

[65,] 70 2

[66,] 96 2

[67,] 6 2

[68,] 99 2

[69,] 62 2

[70,] 56 2

[71,] 116 2

[72,] 13 2

[73,] 17 2

[74,] 10 2

[75,] 98 2

[76,] 0 2

[77,] 27 2

[78,] 31 2

[79,] 0 2

[80,] 2 2

[81,] 3 2

[82,] 4 2

- sex ratio

sexratio japBra

[1,] 0.8000000 1

[2,] 0.8205128 1

[3,] 0.8888889 1

[4,] 0.8965517 1

[5,] 0.6470588 1

[6,] 0.8461538 1

[7,] 0.7500000 1

[8,] 0.9545455 1

[9,] 0.8787879 1

[10,] 0.8931298 1

[11,] 0.5000000 1

[12,] 0.6153846 1

[13,] 0.8750000 1

[14,] 0.6250000 1

[15,] 0.5000000 1

[16,] 0.5200000 1

[17,] 0.6600000 1

[18,] 0.9427481 1

[19,] 0.3750000 1

[20,] 0.6875000 1

[21,] 1.0000000 1

[22,] 0.5000000 1

[23,] 0.9183673 1

[24,] 0.8571429 1

[25,] 0.6666667 1

[26,] 1.0000000 1

[27,] 0.6250000 1

[28,] 0.7500000 1

[29,] 0.7500000 1

[30,] 0.8000000 1

[31,] 0.6811594 1

[32,] 0.6666667 1

[33,] 0.3333333 1

[34,] 0.6875000 1

[35,] 0.3750000 1

[36,] 0.5714286 1

[37,] 0.8000000 1

[38,] 0.0000000 1

[39,] 0.7647059 1

[40,] 0.5000000 1

[41,] 0.6315789 1

[42,] 0.3333333 1

[43,] 0.2222222 1

[44,] 0.5000000 1

[45,] 0.6666667 1

[46,] 0.6250000 1

[47,] 0.9090909 1

[48,] 1.0000000 1

[49,] 0.9130435 1

[50,] 1.0000000 1

[51,] 1.0000000 1

[52,] 1.0000000 1

[53,] 0.4000000 1

[54,] 0.8000000 2

[55,] 0.8205128 2

[56,] 0.8888889 2

[57,] 0.8965517 2

[58,] 0.6470588 2

[59,] 0.8461538 2

[60,] 0.7500000 2

[61,] 0.9545455 2

[62,] 0.8787879 2

[63,] 0.8931298 2

[64,] 0.5000000 2

[65,] 0.6153846 2

[66,] 0.8750000 2

[67,] 0.6250000 2

[68,] 0.5000000 2

[69,] 0.5200000 2

[70,] 0.6600000 2

[71,] 0.9427481 2

[72,] 0.3750000 2

[73,] 0.6875000 2

[74,] 1.0000000 2

[75,] 0.5000000 2

[76,] 0.9183673 2

[77,] 0.8571429 2

- mean number of workers

meanWorkers japBra

[1,] 19.20588 1

[2,] 20.47458 1

[3,] 13.20000 1

[4,] 24.68000 1

[5,] 35.08696 1

[6,] 17.22222 1

[7,] 30.56000 1

[8,] 29.33333 1

[9,] 26.04167 1

[10,] 24.57778 1

[11,] 25.11111 1

[12,] 14.93333 1

[13,] 26.00000 1

[14,] 27.64000 1

[15,] 10.74468 1

[16,] 19.56818 1

[17,] 17.64286 1

[18,] 16.79775 1

[19,] 32.43243 1

[20,] 22.42500 1

[21,] 14.61111 1

[22,] 22.95238 1

[23,] 13.86667 1

[24,] 14.54545 1

[25,] 31.76667 1

[26,] 14.91892 1

[27,] 15.59375 1

[28,] 23.43478 1

[29,] 14.86667 1

[30,] 29.03571 1

[31,] 27.40000 1

[32,] 15.50943 1

[33,] 37.40000 1

[34,] 19.40000 1

[35,] 16.02439 1

[36,] 27.73333 1

[37,] 26.54167 1

[38,] 28.32258 1

[39,] 14.97297 1

[40,] 18.43182 1

[41,] 25.85714 1

[42,] 17.13514 1

[43,] 21.47619 1

[44,] 12.53846 1

[45,] 16.10256 1

[46,] 10.80392 1

[47,] 15.02778 1

[48,] 20.85714 1

[49,] 12.41176 1

[50,] 11.94737 1

[51,] 26.80000 1

[52,] 20.38710 1

[53,] 16.95238 1

[54,] 12.78571 1

[55,] 10.41176 1

[56,] 17.60000 1

[57,] 13.00000 2

[58,] 16.70588 2

[59,] 18.90476 2

[60,] 16.76923 2

[61,] 36.04762 2

[62,] 14.25000 2

[63,] 30.05882 2

[64,] 17.84375 2

[65,] 31.62000 2

[66,] 48.42105 2

[67,] 15.81481 2

[68,] 51.18367 2

[69,] 38.62745 2

[70,] 39.68293 2

[71,] 25.88636 2

[72,] 16.82353 2

[73,] 36.59259 2

[74,] 20.31429 2

[75,] 49.61818 2

[76,] 17.90476 2

[77,] 23.34694 2

[78,] 23.35897 2

[79,] 17.72222 2

[80,] 13.40000 2

[81,] 17.62500 2

[82,] 22.70833 2

- maximum number of workers

maxWorkers japBra

[1,] 26 1

[2,] 38 1

[3,] 20 1

[4,] 37 1

[5,] 68 1

[6,] 25 1

[7,] 64 1

[8,] 49 1

[9,] 48 1

[10,] 40 1

[11,] 46 1

[12,] 20 1

[13,] 43 1

[14,] 50 1

[15,] 28 1

[16,] 29 1

[17,] 47 1

[18,] 38 1

[19,] 66 1

[20,] 30 1

[21,] 20 1

[22,] 39 1

[23,] 20 1

[24,] 23 1

[25,] 51 1

[26,] 25 1

[27,] 27 1

[28,] 49 1

[29,] 20 1

[30,] 48 1

[31,] 59 1

[32,] 31 1

[33,] 78 1

[34,] 30 1

[35,] 20 1

[36,] 55 1

[37,] 49 1

[38,] 45 1

[39,] 20 1

[40,] 29 1

[41,] 43 1

[42,] 21 1

[43,] 39 1

[44,] 20 1

[45,] 21 1

[46,] 20 1

[47,] 22 1

[48,] 29 1

[49,] 20 1

[50,] 20 1

[51,] 50 1

[52,] 26 1

[53,] 23 1

[54,] 20 1

[55,] 20 1

[56,] 20 1

[57,] 20 2

[58,] 20 2

[59,] 22 2

[60,] 20 2

[61,] 83 2

[62,] 25 2

[63,] 83 2

[64,] 20 2

[65,] 56 2

[66,] 105 2

[67,] 20 2

[68,] 107 2

[69,] 83 2

[70,] 82 2

[71,] 41 2

[72,] 22 2

[73,] 75 2

[74,] 30 2

[75,] 104 2

[76,] 20 2

[77,] 32 2

[78,] 40 2

[79,] 20 2

[80,] 20 2

[81,] 29 2

[82,] 41 2

- total number of workers

totalWorkers japBra

[1,] 95 1

[2,] 1 2

[3,] 91 1

[4,] 224 2

[5,] 152 1

[6,] 1 2

[7,] 230 1

[8,] 240 2

[9,] 207 2

[10,] 153 2

[11,] 26 1

[12,] 97 2

[13,] 74 1

[14,] 27 2

[15,] 83 2

[16,] 100 1

[17,] 199 2

[18,] 44 2

[19,] 100 1

[20,] 2 2

[21,] 113 1

[22,] NA 2

[23,] 80 1

[24,] -9 2

[25,] 179 1

[26,] 57 2

[27,] 188 1

[28,] 62 2

[29,] 159 1

[30,] -14 2

[31,] 119 1

[32,] 55 1

[33,] NA 2

[34,] 32 1

[35,] 0 2

[36,] 19 1

[37,] NA 2

[38,] 26 1

[39,] 22 2

[40,] 16 1

[41,] -11 2

[42,] 105 1

[43,] 37 2

[44,] 68 1

[45,] 37 1

[46,] 58 1

[47,] 32 1

[48,] 145 1

[49,] 73 1

[50,] 123 1

[51,] 273 1

[52,] 46 1

[53,] 56 1

[54,] 1 2

[55,] 83 1

[56,] 142 1

[57,] 70 1

[58,] 211 1

[59,] 21 1

[60,] 94 1

[61,] 80 1

[62,] 35 1

[63,] 119 1

[64,] 143 1

[65,] 149 2

[66,] 48 1

[67,] 93 1

[68,] 67 1

[69,] 55 1

[70,] 32 1

[71,] 47 1

[72,] 58 2

[73,] 44 1

[74,] 27 1

[75,] -5 1

[76,] -1 1

[77,] 152 1

[78,] 92 1

[79,] 147 2

[80,] 50 1

[81,] 5 1

[82,] -4 1

[83,] 26 1

[84,] -15 1

[85,] 66 1

[86,] 3 2

[87,] 93 1

[88,] 72 2

-onset of sexual reproduction

Age1queenPupae japBra

[1,] 110 2

[2,] NA 2

[3,] 71 2

[4,] 30 2

[5,] 89 2

[6,] 85 2

[7,] 134 2

[8,] NA 2

[9,] 85 2

[10,] 71 2

[11,] 78 2

[12,] 89 2

[13,] 82 2

[14,] 69 2

[15,] 75 2

[16,] 74 2

[17,] 61 2

[18,] 82 2

[19,] 85 2

[20,] NA 2

[21,] 79 2

[22,] 78 2

[23,] NA 2

[24,] NA 2

[25,] 61 2

[26,] 69 2

[27,] 76 1

[28,] 71 1

[29,] NA 1

[30,] 66 1

[31,] 67 1

[32,] 74 1

[33,] 79 1

[34,] 50 1

[35,] 56 1

[36,] 72 1

[37,] 78 1

[38,] 60 1

[39,] 77 1

[40,] 77 1

[41,] 60 1

[42,] 81 1

[43,] 74 1

[44,] 91 1

[45,] 60 1

[46,] 81 1

[47,] 50 1

[48,] 67 1

[49,] NA 1

[50,] 74 1

[51,] 70 1

[52,] 59 1

[53,] 60 1

[54,] 68 1

[55,] 53 1

[56,] 63 1

[57,] 71 1

[58,] 67 1

[59,] 66 1

[60,] 65 1

[61,] 66 1

[62,] 81 1

[63,] 68 1

[64,] 47 1

[65,] 60 1

[66,] NA 1

[67,] 71 1

[68,] 58 1

[69,] 88 1

[70,] 128 1

[71,] 101 1

[72,] 121 1

[73,] 76 1

[74,] 72 1

[75,] 47 1

[76,] 54 1

[77,] 71 1

[78,] 67 1

[79,] 50 1

[80,] 71 1

[81,] 85 1

[82,] NA 1

Age1MalePupae japBra

[1,] 99 2

[2,] 89 2

[3,] NA 2

[4,] 40 2

[5,] 100 2

[6,] 68 2

[7,] 137 2

[8,] 94 2

[9,] 82 2

[10,] 64 2

[11,] 54 2

[12,] 79 2

[13,] 85 2

[14,] 58 2

[15,] 71 2

[16,] 50 2

[17,] 71 2

[18,] 96 2

[19,] 79 2

[20,] NA 2

[21,] 61 2

[22,] 61 2

[23,] NA 2

[24,] 76 2

[25,] NA 2

[26,] 63 2

[27,] 76 1

[28,] 75 1

[29,] NA 1

[30,] 76 1

[31,] 74 1

[32,] 74 1

[33,] 72 1

[34,] 63 1

[35,] 105 1

[36,] 75 1

[37,] 99 1

[38,] 60 1

[39,] 81 1

[40,] 74 1

[41,] 57 1

[42,] 74 1

[43,] 74 1

[44,] 85 1

[45,] 74 1

[46,] 64 1

[47,] 88 1

[48,] NA 1

[49,] NA 1

[50,] 92 1

[51,] 63 1

[52,] 98 1

[53,] 60 1

[54,] NA 1

[55,] 66 1

[56,] 63 1

[57,] 71 1

[58,] 88 1

[59,] 56 1

[60,] 54 1

[61,] 59 1

[62,] 67 1

[63,] 61 1

[64,] 44 1

[65,] 64 1

[66,] 97 1

[67,] 74 1

[68,] 58 1

[69,] 80 1

[70,] 104 1

[71,] 79 1

[72,] 114 1

[73,] 90 1

[74,] 107 1

[75,] 36 1

[76,] NA 1

[77,] 92 1

[78,] NA 1

[79,] NA 1

[80,] NA 1

[81,] 111 1

[82,] NA 1

- queen lifespan & body size

queensize qls japBra

[1,] NA 100 1

[2,] 0.5590 174 2

[3,] NA 147 1

[4,] NA 197 2

[5,] NA 297 1

[6,] NA 95 2

[7,] 0.6622 236 1

[8,] NA 175 2

[9,] NA 183 2

[10,] NA 145 2

[11,] 0.6020 112 1

[12,] NA 185 2

[13,] 0.6106 112 1

[14,] NA 119 2

[15,] NA 123 2

[16,] 0.6536 81 1

[17,] 0.6106 195 2

[18,] NA NA 2

[19,] 0.6278 182 1

[20,] 0.6536 112 2

[21,] NA 342 1

[22,] NA NA 2

[23,] 0.6794 75 1

[24,] NA 73 2

[25,] NA 322 1

[26,] NA 169 2

[27,] 0.6192 286 1

[28,] NA 131 2

[29,] NA 318 1

[30,] NA 66 2

[31,] NA 137 1

[32,] 0.6794 132 1

[33,] NA NA 2

[34,] NA 71 1

[35,] NA 97 2

[36,] NA 101 1

[37,] NA NA 2

[38,] NA 109 1

[39,] NA 76 2

[40,] 0.6020 108 1

[41,] NA 71 2

[42,] NA 97 1

[43,] NA 72 2

[44,] 0.6020 96 1

[45,] 0.6278 141 1

[46,] NA 93 1

[47,] 0.6364 104 1

[48,] 0.7224 86 1

[49,] NA 86 1

[50,] 0.6880 205 1

[51,] 0.6708 168 1

[52,] 0.6278 36 1

[53,] NA 75 1

[54,] NA 43 2

[55,] 0.6278 144 1

[56,] 0.6536 94 1

[57,] 0.6622 94 1

[58,] 0.6364 121 1

[59,] 0.7224 122 1

[60,] NA 171 1

[61,] 0.6708 87 1

[62,] NA 146 1

[63,] 0.6192 118 1

[64,] 0.7052 255 1

[65,] NA 117 2

[66,] 0.6794 136 1

[67,] 0.6708 108 1

[68,] NA 160 1

[69,] 0.6364 193 1

[70,] 0.6192 143 1

[71,] NA 140 1

[72,] NA 154 2

[73,] 0.6794 142 1

[74,] 0.6450 112 1

[75,] NA 19 1

[76,] 0.6880 137 1

[77,] NA 161 1

[78,] NA 68 1

[79,] NA 119 2

[80,] NA 92 1

[81,] 0.6450 57 1

[82,] NA 96 1

[83,] 0.6880 151 1

[84,] NA 7 1

[85,] NA 51 1

[86,] 0.6106 103 2

[87,] 0.6020 168 1

[88,] 0.6020 166 2

- number of eggs & body size

queensize totalEggs japBra

[1,] NA 121.082707 1

[2,] 0.5590 127.842857 2

[3,] NA 150.000000 1

[4,] NA 464.202857 2

[5,] NA 383.815552 1

[6,] NA 78.000000 2

[7,] 0.6622 434.857143 1

[8,] NA 366.240000 2

[9,] NA 467.750000 2

[10,] NA 304.922857 2

[11,] 0.6020 51.948052 1

[12,] NA 335.867143 2

[13,] 0.6106 175.155280 1

[14,] NA 205.982857 2

[15,] NA 180.714286 2

[16,] 0.6536 127.384615 1

[17,] 0.6106 560.657143 2

[18,] NA 135.651429 2

[19,] 0.6278 210.000000 1

[20,] 0.6536 69.440000 2

[21,] NA 300.771429 1

[22,] NA 7.542857 2

[23,] 0.6794 57.272727 1

[24,] NA 26.954286 2

[25,] NA 431.375661 1

[26,] NA 246.705714 2

[27,] 0.6192 408.205128 1

[28,] NA 261.638571 2

[29,] NA 462.523810 1

[30,] NA 62.971429 2

[31,] NA 191.055556 1

[32,] 0.6794 119.532020 1

[33,] NA 0.000000 2

[34,] NA 0.000000 1

[35,] NA 172.354286 2

[36,] NA 0.000000 1

[37,] NA 0.000000 2

[38,] NA 98.000000 1

[39,] NA 110.550000 2

[40,] 0.6020 80.888889 1

[41,] NA 50.571429 2

[42,] NA 124.500000 1

[43,] NA 116.550000 2

[44,] 0.6020 122.500000 1

[45,] 0.6278 80.071429 1

[46,] NA 89.415584 1

[47,] 0.6364 79.040000 1

[48,] 0.7224 158.571429 1

[49,] NA 92.077143 1

[50,] 0.6880 255.862857 1

[51,] 0.6708 379.034843 1

[52,] 0.6278 11.931429 1

[53,] NA 72.000000 1

[54,] NA 18.285714 2

[55,] 0.6278 190.324286 1

[56,] 0.6536 232.333333 1

[57,] 0.6622 74.151429 1

[58,] 0.6364 92.650000 1

[59,] 0.7224 90.108225 1

[60,] NA 156.274286 1

[61,] 0.6708 125.250000 1

[62,] NA 68.914286 1

[63,] 0.6192 164.500000 1

[64,] 0.7052 354.283019 1

[65,] NA 149.460000 2

[66,] 0.6794 93.708571 1

[67,] 0.6708 118.217143 1

[68,] NA 123.918367 1

[69,] 0.6364 165.958571 1

[70,] 0.6192 69.804286 1

[71,] NA 128.653061 1

[72,] NA 211.750000 2

[73,] 0.6794 85.541126 1

[74,] 0.6450 72.800000 1

[75,] NA 0.000000 1

[76,] 0.6880 39.285714 1

[77,] NA 236.179487 1

[78,] NA 106.666667 1

[79,] NA 276.000000 2

[80,] NA 56.520000 1

[81,] 0.6450 0.000000 1

[82,] NA 37.803571 1

[83,] 0.6880 95.142857 1

[84,] NA 0.000000 1

[85,] NA 64.628571 1

[86,] 0.6106 116.022857 2

[87,] 0.6020 190.500000 1

[88,] 0.6020 272.090000 2

**2) Individual level trade-off**

-Fig 1

QLS totEggs japBra

[1,] 100 121.082707 1

[2,] 174 127.842857 2

[3,] 147 150.000000 1

[4,] 197 464.202857 2

[5,] 297 383.815552 1

[6,] 95 78.000000 2

[7,] 236 434.857143 1

[8,] 175 366.240000 2

[9,] 183 467.750000 2

[10,] 145 304.922857 2

[11,] 112 51.948052 1

[12,] 185 335.867143 2

[13,] 112 175.155280 1

[14,] 119 205.982857 2

[15,] 123 180.714286 2

[16,] 81 127.384615 1

[17,] 195 560.657143 2

[18,] NA 135.651429 2

[19,] 182 210.000000 1

[20,] 112 69.440000 2

[21,] 342 300.771429 1

[22,] NA 7.542857 2

[23,] 75 57.272727 1

[24,] 73 26.954286 2

[25,] 322 431.375661 1

[26,] 169 246.705714 2

[27,] 286 408.205128 1

[28,] 131 261.638571 2

[29,] 318 462.523810 1

[30,] 66 62.971429 2

[31,] 137 191.055556 1

[32,] 132 119.532020 1

[33,] NA 0.000000 2

[34,] 71 0.000000 1

[35,] 97 172.354286 2

[36,] 101 0.000000 1

[37,] NA 0.000000 2

[38,] 109 98.000000 1

[39,] 76 110.550000 2

[40,] 108 80.888889 1

[41,] 71 50.571429 2

[42,] 97 124.500000 1

[43,] 72 116.550000 2

[44,] 96 122.500000 1

[45,] 141 80.071429 1

[46,] 93 89.415584 1

[47,] 104 79.040000 1

[48,] 86 158.571429 1

[49,] 86 92.077143 1

[50,] 205 255.862857 1

[51,] 168 379.034843 1

[52,] 36 11.931429 1

[53,] 75 72.000000 1

[54,] 43 18.285714 2

[55,] 144 190.324286 1

[56,] 94 232.333333 1

[57,] 94 74.151429 1

[58,] 121 92.650000 1

[59,] 122 90.108225 1

[60,] 171 156.274286 1

[61,] 87 125.250000 1

[62,] 146 68.914286 1

[63,] 118 164.500000 1

[64,] 255 354.283019 1

[65,] 117 149.460000 2

[66,] 136 93.708571 1

[67,] 108 118.217143 1

[68,] 160 123.918367 1

[69,] 193 165.958571 1

[70,] 143 69.804286 1

[71,] 140 128.653061 1

[72,] 154 211.750000 2

[73,] 142 85.541126 1

[74,] 112 72.800000 1

[75,] 19 0.000000 1

[76,] 137 39.285714 1

[77,] 161 236.179487 1

[78,] 68 106.666667 1

[79,] 119 276.000000 2

[80,] 92 56.520000 1

[81,] 57 0.000000 1

[82,] 96 37.803571 1

[83,] 151 95.142857 1

[84,] 7 0.000000 1

[85,] 51 64.628571 1

[86,] 103 116.022857 2

[87,] 168 190.500000 1

[88,] 166 272.090000 2

- Fig 2 :

QLS eggsperweek japbra

[1,] 100 8.475789 1

[2,] 174 5.143103 2

[3,] 147 7.142857 1

[4,] 197 16.494518 2

[5,] 297 9.046158 1

[6,] 95 5.747368 2

[7,] 236 12.898305 1

[8,] 175 14.649600 2

[9,] 183 17.892077 2

[10,] 145 14.720414 2

[11,] 112 3.246753 1

[12,] 185 12.708486 2

[13,] 112 10.947205 1

[14,] 119 12.116639 2

[15,] 123 10.284553 2

[16,] 81 11.008547 1

[17,] 195 20.126154 2

[18,] NA NA 2

[19,] 182 8.076923 1

[20,] 112 4.340000 2

[21,] 342 6.156140 1

[22,] NA NA 2

[23,] 75 5.345455 1

[24,] 73 2.584658 2

[25,] 322 9.377732 1

[26,] 169 10.218580 2

[27,] 286 9.991035 1

[28,] 131 13.980687 2

[29,] 318 10.181342 1

[30,] 66 6.678788 2

[31,] 137 9.761963 1

[32,] 132 6.338819 1

[33,] NA NA 2

[34,] 71 0.000000 1

[35,] 97 12.437938 2

[36,] 101 0.000000 1

[37,] NA NA 2

[38,] 109 6.293578 1

[39,] 76 10.182237 2

[40,] 108 5.242798 1

[41,] 71 4.985915 2

[42,] 97 8.984536 1

[43,] 72 11.331250 2

[44,] 96 8.932292 1

[45,] 141 3.975177 1

[46,] 93 6.730205 1

[47,] 104 5.320000 1

[48,] 86 12.906977 1

[49,] 86 7.494651 1

[50,] 205 8.736780 1

[51,] 168 15.793118 1

[52,] 36 2.320000 1

[53,] 75 6.720000 1

[54,] 43 2.976744 2

[55,] 144 9.251875 1

[56,] 94 17.301418 1

[57,] 94 5.521915 1

[58,] 121 5.359917 1

[59,] 122 5.170144 1

[60,] 171 6.397193 1

[61,] 87 10.077586 1

[62,] 146 3.304110 1

[63,] 118 9.758475 1

[64,] 255 9.725416 1

[65,] 117 8.942051 2

[66,] 136 4.823235 1

[67,] 108 7.662222 1

[68,] 160 5.421429 1

[69,] 193 6.019223 1

[70,] 143 3.416993 1

[71,] 140 6.432653 1

[72,] 154 9.625000 2

[73,] 142 4.216816 1

[74,] 112 4.550000 1

[75,] 19 0.000000 1

[76,] 137 2.007299 1

[77,] 161 10.268673 1

[78,] 68 10.980392 1

[79,] 119 16.235294 2

[80,] 92 4.300435 1

[81,] 57 0.000000 1

[82,] 96 2.756510 1

[83,] 151 4.410596 1

[84,] 7 0.000000 1

[85,] 51 8.870588 1

[86,] 103 7.885049 2

[87,] 168 7.937500 1

[88,] 166 11.473675 2

-Fig 3:

"stdAge" "eggcount" "japBra"

"1" 0.0099009900990099 0 1

"2" 0.128712871287129 1 1

"3" 0.168316831683168 0 1

"4" 0.227722772277228 0 1

"5" 0.297029702970297 5 1

"6" 0.336633663366337 8 1

"7" 0.366336633663366 7 1

"8" 0.405940594059406 13 1

"9" 0.445544554455446 0 1

"10" 0.475247524752475 14 1

"11" 0.504950495049505 8 1

"12" 0.544554455445545 4 1

"13" 0.574257425742574 13 1

"14" 0.613861386138614 16 1

"15" 0.653465346534653 12 1

"16" 0.683168316831683 8 1

"17" 0.712871287128713 16 1

"18" 0.752475247524752 3 1

"19" 0.782178217821782 16 1

"20" 0.821782178217822 8 1

"21" 0.851485148514851 15 1

"22" 0.891089108910891 8 1

"23" 0.920792079207921 8 1

"24" 0.96039603960396 5 1

"25" 1 3 1

"26" 1.02970297029703 2 1

"27" 1.05940594059406 0 1

"28" 1.0990099009901 0 1

"29" 1.12871287128713 0 1

"30" 1.16831683168317 0 1

"31" 1.1980198019802 0 1

"32" 1.23762376237624 0 1

"33" 1.26732673267327 0 1

"34" 1.30693069306931 0 1

"35" 0.00578034682080925 0 1

"36" 0.0289017341040462 1 1

"37" 0.0520231213872832 5 1

"38" 0.069364161849711 5 1

"39" 0.0867052023121387 4 1

"40" 0.109826589595376 5 1

"41" 0.127167630057803 4 1

"42" 0.15028901734104 3 1

"43" 0.173410404624277 3 1

"44" 0.190751445086705 4 1

"45" 0.208092485549133 1 1

"46" 0.23121387283237 6 1

"47" 0.248554913294798 10 1

"48" 0.271676300578035 5 1

"49" 0.289017341040462 11 1

"50" 0.312138728323699 10 1

"51" 0.329479768786127 5 1

"52" 0.352601156069364 10 1

"53" 0.375722543352601 12 1

"54" 0.393063583815029 7 1

"55" 0.410404624277457 12 1

"56" 0.433526011560694 10 1

"57" 0.450867052023121 20 1

"58" 0.473988439306358 16 1

"59" 0.491329479768786 12 1

"60" 0.514450867052023 13 1

"61" 0.531791907514451 5 1

"62" 0.554913294797688 8 1

"63" 0.572254335260116 10 1

"64" 0.595375722543353 9 1

"65" 0.61271676300578 10 1

"66" 0.635838150289017 8 1

"67" 0.653179190751445 12 1

"68" 0.676300578034682 10 1

"69" 0.69364161849711 18 1

"70" 0.716763005780347 15 1

"71" 0.745664739884393 9 1

"72" 0.757225433526012 15 1

"73" 0.774566473988439 9 1

"74" 0.797687861271676 5 1

"75" 0.815028901734104 6 1

"76" 0.838150289017341 6 1

"77" 0.855491329479769 13 1

"78" 0.878612716763006 10 1

"79" 0.895953757225434 10 1

"80" 0.919075144508671 8 1

"81" 0.936416184971098 12 1

"82" 0.959537572254335 11 1

"83" 0.976878612716763 7 1

"84" 1 6 1

"85" 1.01734104046243 2 1

"86" 1.04046242774566 0 1

"87" 1.05780346820809 0 1

"88" 1.08092485549133 0 1

"89" 1.09826589595376 0 1

"90" 1.12138728323699 0 1

"91" 1.13872832369942 0 1

"92" 1.16184971098266 0 1

"93" 1.17919075144509 0 1

"94" 0.00813008130081301 0 1

"95" 0.0569105691056911 0 1

"96" 0.0894308943089431 2 1

"97" 0.113821138211382 8 1

"98" 0.146341463414634 8 1

"99" 0.170731707317073 10 1

"100" 0.203252032520325 8 1

"101" 0.235772357723577 2 1

"102" 0.260162601626016 7 1

"103" 0.284552845528455 5 1

"104" 0.317073170731707 7 1

"105" 0.341463414634146 9 1

"106" 0.373983739837398 8 1

"107" 0.40650406504065 5 1

"108" 0.430894308943089 7 1

"109" 0.455284552845528 6 1

"110" 0.48780487804878 6 1

"111" 0.51219512195122 11 1

"112" 0.544715447154472 10 1

"113" 0.569105691056911 4 1

"114" 0.601626016260163 5 1

"115" 0.626016260162602 4 1

"116" 0.658536585365854 4 1

"117" 0.691056910569106 3 1

"118" 0.715447154471545 3 1

"119" 0.739837398373984 3 1

"120" 0.772357723577236 5 1

"121" 0.796747967479675 3 1

"122" 0.829268292682927 3 1

"123" 0.853658536585366 1 1

"124" 0.886178861788618 0 1

"125" 0.910569105691057 1 1

"126" 0.943089430894309 0 1

"127" 0.967479674796748 0 1

"128" 1 0 1

"129" 0.0131578947368421 0 1

"130" 0.0921052631578947 0 1

"131" 0.171052631578947 4 1

"132" 0.223684210526316 0 1

"133" 0.302631578947368 0 1

"134" 0.394736842105263 6 1

"135" 0.447368421052632 6 1

"136" 0.486842105263158 10 1

"137" 0.539473684210526 20 1

"138" 0.592105263157895 12 1

"139" 0.631578947368421 12 1

"140" 0.671052631578947 12 1

"141" 0.723684210526316 2 1

"142" 0.763157894736842 5 1

"143" 0.815789473684211 7 1

"144" 0.868421052631579 9 1

"145" 0.907894736842105 5 1

"146" 0.947368421052632 2 1

"147" 1 4 1

"148" 1.03947368421053 2 1

"149" 1.09210526315789 0 1

"150" 1.13157894736842 0 1

"151" 1.18421052631579 0 1

"152" 1.22368421052632 0 1

"153" 1.27631578947368 0 1

"154" 0.00413223140495868 0 1

"155" 0.0165289256198347 0 1

"156" 0.0330578512396694 0 1

"157" 0.0454545454545455 2 1

"158" 0.0619834710743802 6 1

"159" 0.0743801652892562 10 1

"160" 0.0909090909090909 8 1

"161" 0.107438016528926 5 1

"162" 0.119834710743802 6 1

"163" 0.132231404958678 6 1

"164" 0.148760330578512 8 1

"165" 0.161157024793388 12 1

"166" 0.177685950413223 10 1

"167" 0.194214876033058 16 1

"168" 0.206611570247934 10 1

"169" 0.21900826446281 11 1

"170" 0.235537190082645 8 1

"171" 0.247933884297521 11 1

"172" 0.264462809917355 7 1

"173" 0.276859504132231 15 1

"174" 0.293388429752066 18 1

"175" 0.305785123966942 16 1

"176" 0.322314049586777 12 1

"177" 0.338842975206612 16 1

"178" 0.351239669421488 17 1

"179" 0.363636363636364 17 1

"180" 0.380165289256198 12 1

"181" 0.392561983471074 13 1

"182" 0.409090909090909 10 1

"183" 0.421487603305785 18 1

"184" 0.43801652892562 17 1

"185" 0.450413223140496 10 1

"186" 0.466942148760331 20 1

"187" 0.479338842975207 20 1

"188" 0.495867768595041 21 1

"189" 0.508264462809917 18 1

"190" 0.524793388429752 10 1

"191" 0.537190082644628 17 1

"192" 0.553719008264463 14 1

"193" 0.566115702479339 13 1

"194" 0.582644628099174 18 1

"195" 0.603305785123967 4 1

"196" 0.611570247933884 7 1

"197" 0.62396694214876 5 1

"198" 0.640495867768595 10 1

"199" 0.652892561983471 8 1

"200" 0.669421487603306 2 1

"201" 0.681818181818182 5 1

"202" 0.698347107438017 7 1

"203" 0.710743801652893 9 1

"204" 0.727272727272727 7 1

"205" 0.739669421487603 1 1

"206" 0.756198347107438 5 1

"207" 0.768595041322314 9 1

"208" 0.785123966942149 8 1

"209" 0.797520661157025 4 1

"210" 0.814049586776859 1 1

"211" 0.826446280991736 0 1

"212" 0.84297520661157 0 1

"213" 0.855371900826446 1 1

"214" 0.871900826446281 0 1

"215" 0.884297520661157 2 1

"216" 0.900826446280992 2 1

"217" 0.913223140495868 2 1

"218" 0.929752066115702 0 1

"219" 0.942148760330578 3 1

"220" 0.975206611570248 0 1

"221" 0.987603305785124 0 1

"222" 1 0 1

"223" 0.00714285714285714 0 1

"224" 0.0857142857142857 3 1

"225" 0.128571428571429 0 1

"226" 0.157142857142857 0 1

"227" 0.2 0 1

"228" 0.25 0 1

"229" 0.278571428571429 0 1

"230" 0.3 0 1

"231" 0.328571428571429 4 1

"232" 0.357142857142857 5 1

"233" 0.378571428571429 8 1

"234" 0.4 7 1

"235" 0.428571428571429 8 1

"236" 0.45 8 1

"237" 0.478571428571429 9 1

"238" 0.507142857142857 8 1

"239" 0.528571428571429 10 1

"240" 0.55 8 1

"241" 0.578571428571429 9 1

"242" 0.6 8 1

"243" 0.628571428571429 10 1

"244" 0.65 7 1

"245" 0.678571428571429 7 1

"246" 0.7 10 1

"247" 0.728571428571429 7 1

"248" 0.757142857142857 9 1

"249" 0.778571428571429 7 1

"250" 0.8 5 1

"251" 0.828571428571429 5 1

"252" 0.85 10 1

"253" 0.878571428571429 8 1

"254" 0.9 7 1

"255" 0.928571428571429 5 1

"256" 0.95 0 1

"257" 0.978571428571429 5 1

"258" 1 0 1

"259" 1.02857142857143 0 1

"260" 1.05 0 1

"261" 1.07857142857143 0 1

"262" 1.1 0 1

"263" 1.12857142857143 0 1

"264" 1.15 0 1

"265" 1.17857142857143 0 1

"266" 1.21428571428571 0 1

"267" 1.22857142857143 0 1

"268" 0.0144927536231884 0 1

"269" 0.101449275362319 0 1

"270" 0.188405797101449 5 1

"271" 0.246376811594203 0 1

"272" 0.333333333333333 0 1

"273" 0.434782608695652 6 1

"274" 0.492753623188406 6 1

"275" 0.536231884057971 10 1

"276" 0.594202898550725 10 1

"277" 0.652173913043478 20 1

"278" 0.695652173913043 17 1

"279" 0.739130434782609 14 1

"280" 0.797101449275362 18 1

"281" 0.840579710144927 24 1

"282" 0.898550724637681 20 1

"283" 0.956521739130435 10 1

"284" 1 5 1

"285" 1.04347826086957 0 1

"286" 1.10144927536232 0 1

"287" 1.14492753623188 0 1

"288" 1.20289855072464 0 1

"289" 1.2463768115942 0 1

"290" 1.30434782608696 0 1

"291" 1.34782608695652 0 1

"292" 1.40579710144928 0 1

"293" 0.0217391304347826 0 1

"294" 0.152173913043478 6 1

"295" 0.260869565217391 0 1

"296" 0.391304347826087 0 1

"297" 0.478260869565217 0 1

"298" 0.608695652173913 0 1

"299" 0.760869565217391 15 1

"300" 0.847826086956522 15 1

"301" 0.91304347826087 15 1

"302" 1 12 1

"303" 1.08695652173913 1 1

"304" 1.15217391304348 0 1

"305" 1.21739130434783 0 1

"306" 1.30434782608696 0 1

"307" 1.3695652173913 0 1

"308" 1.45652173913043 0 1

"309" 1.54347826086957 0 1

"310" 1.60869565217391 0 1

"311" 1.67391304347826 0 1

"312" 1.76086956521739 0 1

"313" 1.91304347826087 0 1

"314" 0.00595238095238095 0 1

"315" 0.0416666666666667 1 1

"316" 0.0595238095238095 2 1

"317" 0.0833333333333333 10 1

"318" 0.101190476190476 10 1

"319" 0.125 8 1

"320" 0.148809523809524 7 1

"321" 0.166666666666667 10 1

"322" 0.18452380952381 6 1

"323" 0.208333333333333 7 1

"324" 0.226190476190476 12 1

"325" 0.25 7 1

"326" 0.273809523809524 11 1

"327" 0.291666666666667 14 1

"328" 0.30952380952381 19 1

"329" 0.333333333333333 10 1

"330" 0.351190476190476 15 1

"331" 0.375 8 1

"332" 0.392857142857143 14 1

"333" 0.416666666666667 15 1

"334" 0.43452380952381 13 1

"335" 0.458333333333333 15 1

"336" 0.482142857142857 8 1

"337" 0.5 3 1

"338" 0.517857142857143 17 1

"339" 0.541666666666667 18 1

"340" 0.55952380952381 13 1

"341" 0.583333333333333 10 1

"342" 0.601190476190476 18 1

"343" 0.625 15 1

"344" 0.642857142857143 10 1

"345" 0.666666666666667 7 1

"346" 0.68452380952381 3 1

"347" 0.708333333333333 4 1

"348" 0.726190476190476 2 1

"349" 0.75 6 1

"350" 0.767857142857143 5 1

"351" 0.791666666666667 0 1

"352" 0.80952380952381 0 1

"353" 0.833333333333333 0 1

"354" 0.863095238095238 5 1

"355" 0.875 4 1

"356" 0.892857142857143 6 1

"357" 0.916666666666667 7 1

"358" 0.93452380952381 4 1

"359" 0.958333333333333 1 1

"360" 0.976190476190476 1 1

"361" 1 0 1

"362" 0.00675675675675676 0 1

"363" 0.0337837837837838 0 1

"364" 0.0540540540540541 1 1

"365" 0.0810810810810811 6 1

"366" 0.108108108108108 6 1

"367" 0.128378378378378 8 1

"368" 0.148648648648649 8 1

"369" 0.175675675675676 8 1

"370" 0.195945945945946 5 1

"371" 0.222972972972973 6 1

"372" 0.25 7 1

"373" 0.27027027027027 6 1

"374" 0.290540540540541 7 1

"375" 0.317567567567568 10 1

"376" 0.337837837837838 9 1

"377" 0.364864864864865 10 1

"378" 0.385135135135135 15 1

"379" 0.412162162162162 15 1

"380" 0.432432432432432 15 1

"381" 0.459459459459459 5 1

"382" 0.486486486486487 6 1

"383" 0.506756756756757 7 1

"384" 0.527027027027027 9 1

"385" 0.554054054054054 5 1

"386" 0.574324324324324 9 1

"387" 0.601351351351351 8 1

"388" 0.621621621621622 10 1

"389" 0.648648648648649 12 1

"390" 0.668918918918919 10 1

"391" 0.695945945945946 0 1

"392" 0.716216216216216 5 1

"393" 0.743243243243243 3 1

"394" 0.763513513513513 5 1

"395" 0.790540540540541 6 1

"396" 0.810810810810811 5 1

"397" 0.837837837837838 10 1

"398" 0.858108108108108 10 1

"399" 0.885135135135135 5 1

"400" 0.918918918918919 8 1

"401" 0.932432432432432 5 1

"402" 0.952702702702703 0 1

"403" 0.97972972972973 0 1

"404" 1 0 1

"405" 1.02702702702703 0 1

"406" 1.0472972972973 0 1

"407" 0.00335570469798658 0 1

"408" 0.0134228187919463 0 1

"409" 0.0268456375838926 0 1

"410" 0.0369127516778524 0 1

"411" 0.0503355704697987 2 1

"412" 0.063758389261745 7 1

"413" 0.0738255033557047 7 1

"414" 0.0838926174496644 8 1

"415" 0.0973154362416107 5 1

"416" 0.10738255033557 4 1

"417" 0.120805369127517 5 1

"418" 0.134228187919463 7 1

"419" 0.144295302013423 6 1

"420" 0.154362416107383 8 1

"421" 0.167785234899329 6 1

"422" 0.177852348993289 8 1

"423" 0.191275167785235 8 1

"424" 0.201342281879195 15 1

"425" 0.214765100671141 10 1

"426" 0.224832214765101 9 1

"427" 0.238255033557047 10 1

"428" 0.251677852348993 9 1

"429" 0.261744966442953 4 1

"430" 0.271812080536913 11 1

"431" 0.285234899328859 12 1

"432" 0.295302013422819 10 1

"433" 0.308724832214765 11 1

"434" 0.318791946308725 15 1

"435" 0.332214765100671 10 1

"436" 0.342281879194631 7 1

"437" 0.355704697986577 5 1

"438" 0.365771812080537 5 1

"439" 0.379194630872483 5 1

"440" 0.389261744966443 11 1

"441" 0.402684563758389 15 1

"442" 0.412751677852349 12 1

"443" 0.426174496644295 9 1

"444" 0.436241610738255 13 1

"445" 0.449664429530201 8 1

"446" 0.466442953020134 13 1

"447" 0.473154362416107 16 1

"448" 0.483221476510067 12 1

"449" 0.496644295302013 9 1

"450" 0.506711409395973 11 1

"451" 0.520134228187919 10 1

"452" 0.530201342281879 7 1

"453" 0.543624161073825 15 1

"454" 0.553691275167785 11 1

"455" 0.567114093959732 10 1

"456" 0.577181208053691 12 1

"457" 0.590604026845638 14 1

"458" 0.600671140939597 3 1

"459" 0.614093959731544 7 1

"460" 0.624161073825503 14 1

"461" 0.63758389261745 12 1

"462" 0.647651006711409 9 1

"463" 0.661073825503356 17 1

"464" 0.671140939597315 13 1

"465" 0.684563758389262 15 1

"466" 0.694630872483222 19 1

"467" 0.708053691275168 18 1

"468" 0.718120805369127 13 1

"469" 0.731543624161074 18 1

"470" 0.741610738255034 5 1

"471" 0.768456375838926 10 1

"472" 0.778523489932886 10 1

"473" 0.788590604026846 12 1

"474" 0.802013422818792 9 1

"475" 0.812080536912752 7 1

"476" 0.825503355704698 5 1

"477" 0.838926174496644 5 1

"478" 0.848993288590604 2 1

"479" 0.86241610738255 13 1

"480" 0.87248322147651 10 1

"481" 0.88255033557047 14 1

"482" 0.895973154362416 8 1

"483" 0.909395973154362 10 1

"484" 0.919463087248322 11 1

"485" 0.932885906040268 10 1

"486" 0.942953020134228 0 1

"487" 0.956375838926175 4 1

"488" 0.966442953020134 7 1

"489" 0.976510067114094 3 1

"490" 0.98993288590604 0 1

"491" 1 0 1

"492" 1.01677852348993 0 1

"493" 1.03020134228188 0 1

"494" 1.03691275167785 0 1

"495" 1.04697986577181 0 1

"496" 1.06040268456376 0 1

"497" 0.00892857142857143 0 1

"498" 0.107142857142857 4 1

"499" 0.160714285714286 0 1

"500" 0.196428571428571 0 1

"501" 0.223214285714286 0 1

"502" 0.25 0 1

"503" 0.3125 5 1

"504" 0.348214285714286 7 1

"505" 0.375 8 1

"506" 0.410714285714286 5 1

"507" 0.446428571428571 6 1

"508" 0.473214285714286 5 1

"509" 0.5 5 1

"510" 0.535714285714286 3 1

"511" 0.5625 4 1

"512" 0.598214285714286 4 1

"513" 0.633928571428571 5 1

"514" 0.660714285714286 3 1

"515" 0.6875 0 1

"516" 0.723214285714286 2 1

"517" 0.75 1 1

"518" 0.785714285714286 1 1

"519" 0.8125 2 1

"520" 0.848214285714286 0 1

"521" 0.875 5 1

"522" 0.910714285714286 4 1

"523" 0.946428571428571 1 1

"524" 0.973214285714286 2 1

"525" 1 0 1

"526" 1.03571428571429 0 1

"527" 0.00892857142857143 0 1

"528" 0.107142857142857 5 1

"529" 0.160714285714286 0 1

"530" 0.196428571428571 0 1

"531" 0.25 0 1

"532" 0.3125 11 1

"533" 0.348214285714286 13 1

"534" 0.375 15 1

"535" 0.410714285714286 20 1

"536" 0.446428571428571 16 1

"537" 0.473214285714286 8 1

"538" 0.5 16 1

"539" 0.535714285714286 15 1

"540" 0.5625 16 1

"541" 0.598214285714286 15 1

"542" 0.633928571428571 11 1

"543" 0.660714285714286 11 1

"544" 0.6875 10 1

"545" 0.723214285714286 10 1

"546" 0.75 13 1

"547" 0.785714285714286 10 1

"548" 0.8125 12 1

"549" 0.848214285714286 20 1

"550" 0.875 8 1

"551" 0.910714285714286 15 1

"552" 0.946428571428571 7 1

"553" 0.973214285714286 5 1

"554" 1 0 1

"555" 1.03571428571429 0 1

"556" 1.0625 0 1

"557" 1.09821428571429 0 1

"558" 1.125 0 1

"559" 1.16071428571429 0 1

"560" 1.1875 0 1

"561" 0.0123456790123457 0 1

"562" 0.148148148148148 3 1

"563" 0.222222222222222 0 1

"564" 0.271604938271605 0 1

"565" 0.345679012345679 0 1

"566" 0.432098765432099 6 1

"567" 0.481481481481481 11 1

"568" 0.518518518518518 10 1

"569" 0.567901234567901 7 1

"570" 0.617283950617284 15 1

"571" 0.654320987654321 17 1

"572" 0.691358024691358 21 1

"573" 0.740740740740741 17 1

"574" 0.777777777777778 20 1

"575" 0.827160493827161 15 1

"576" 0.876543209876543 16 1

"577" 0.91358024691358 10 1

"578" 0.950617283950617 2 1

"579" 1 0 1

"580" 1.03703703703704 0 1

"581" 1.08641975308642 0 1

"582" 1.12345679012346 0 1

"583" 1.17283950617284 0 1

"584" 1.20987654320988 0 1

"585" 1.25925925925926 0 1

"586" 0.00303030303030303 0 1

"587" 0.0121212121212121 0 1

"588" 0.0333333333333333 7 1

"589" 0.0454545454545455 8 1

"590" 0.0545454545454545 8 1

"591" 0.0666666666666667 8 1

"592" 0.0787878787878788 7 1

"593" 0.0878787878787879 9 1

"594" 0.096969696969697 8 1

"595" 0.109090909090909 8 1

"596" 0.118181818181818 8 1

"597" 0.13030303030303 13 1

"598" 0.142424242424242 11 1

"599" 0.151515151515152 7 1

"600" 0.160606060606061 11 1

"601" 0.172727272727273 8 1

"602" 0.181818181818182 8 1

"603" 0.193939393939394 7 1

"604" 0.203030303030303 4 1

"605" 0.215151515151515 5 1

"606" 0.224242424242424 1 1

"607" 0.236363636363636 10 1

"608" 0.248484848484848 15 1

"609" 0.257575757575758 9 1

"610" 0.266666666666667 11 1

"611" 0.278787878787879 10 1

"612" 0.287878787878788 6 1

"613" 0.3 8 1

"614" 0.309090909090909 6 1

"615" 0.321212121212121 10 1

"616" 0.33030303030303 8 1

"617" 0.342424242424242 7 1

"618" 0.351515151515152 6 1

"619" 0.363636363636364 8 1

"620" 0.372727272727273 6 1

"621" 0.384848484848485 6 1

"622" 0.393939393939394 2 1

"623" 0.406060606060606 1 1

"624" 0.415151515151515 3 1

"625" 0.427272727272727 5 1

"626" 0.442424242424242 4 1

"627" 0.448484848484848 8 1

"628" 0.457575757575758 1 1

"629" 0.46969696969697 0 1

"630" 0.478787878787879 0 1

"631" 0.490909090909091 0 1

"632" 0.5 0 1

"633" 0.512121212121212 5 1

"634" 0.521212121212121 0 1

"635" 0.533333333333333 3 1

"636" 0.542424242424242 5 1

"637" 0.554545454545455 2 1

"638" 0.563636363636364 2 1

"639" 0.575757575757576 2 1

"640" 0.584848484848485 2 1

"641" 0.596969696969697 5 1

"642" 0.606060606060606 3 1

"643" 0.618181818181818 3 1

"644" 0.627272727272727 0 1

"645" 0.639393939393939 5 1

"646" 0.648484848484848 6 1

"647" 0.660606060606061 4 1

"648" 0.66969696969697 6 1

"649" 0.681818181818182 7 1

"650" 0.690909090909091 6 1

"651" 0.715151515151515 4 1

"652" 0.724242424242424 11 1

"653" 0.733333333333333 8 1

"654" 0.745454545454545 11 1

"655" 0.754545454545455 9 1

"656" 0.766666666666667 8 1

"657" 0.778787878787879 21 1

"658" 0.787878787878788 13 1

"659" 0.8 10 1

"660" 0.809090909090909 6 1

"661" 0.818181818181818 6 1

"662" 0.83030303030303 8 1

"663" 0.842424242424242 14 1

"664" 0.851515151515152 10 1

"665" 0.863636363636364 13 1

"666" 0.872727272727273 5 1

"667" 0.884848484848485 16 1

"668" 0.893939393939394 16 1

"669" 0.903030303030303 5 1

"670" 0.915151515151515 10 1

"671" 0.924242424242424 1 1

"672" 0.939393939393939 10 1

"673" 0.951515151515152 0 1

"674" 0.957575757575758 3 1

"675" 0.966666666666667 6 1

"676" 0.978787878787879 3 1

"677" 0.987878787878788 5 1

"678" 1 2 1

"679" 1.00909090909091 0 1

"680" 0.00588235294117647 0 1

"681" 0.0411764705882353 1 1

"682" 0.105882352941176 0 1

"683" 0.129411764705882 0 1

"684" 0.164705882352941 0 1

"685" 0.205882352941176 9 1

"686" 0.229411764705882 8 1

"687" 0.247058823529412 10 1

"688" 0.270588235294118 10 1

"689" 0.294117647058824 5 1

"690" 0.311764705882353 12 1

"691" 0.329411764705882 8 1

"692" 0.352941176470588 6 1

"693" 0.370588235294118 4 1

"694" 0.394117647058824 3 1

"695" 0.417647058823529 9 1

"696" 0.435294117647059 7 1

"697" 0.452941176470588 6 1

"698" 0.476470588235294 6 1

"699" 0.494117647058824 6 1

"700" 0.517647058823529 5 1

"701" 0.535294117647059 8 1

"702" 0.558823529411765 8 1

"703" 0.576470588235294 8 1

"704" 0.6 7 1

"705" 0.623529411764706 6 1

"706" 0.641176470588235 2 1

"707" 0.658823529411765 7 1

"708" 0.682352941176471 8 1

"709" 0.7 10 1

"710" 0.723529411764706 7 1

"711" 0.741176470588235 9 1

"712" 0.764705882352941 7 1

"713" 0.782352941176471 2 1

"714" 0.805882352941176 1 1

"715" 0.823529411764706 0 1

"716" 0.847058823529412 5 1

"717" 0.864705882352941 0 1

"718" 0.888235294117647 0 1

"719" 0.905882352941176 0 1

"720" 0.929411764705882 0 1

"721" 0.947058823529412 0 1

"722" 0.970588235294118 0 1

"723" 1 0 1

"724" 0.0031055900621118 0 1

"725" 0.0372670807453416 1 1

"726" 0.0559006211180124 0 1

"727" 0.0683229813664596 0 1

"728" 0.077639751552795 0 1

"729" 0.0869565217391304 0 1

"730" 0.108695652173913 0 1

"731" 0.12111801242236 4 1

"732" 0.130434782608696 8 1

"733" 0.142857142857143 8 1

"734" 0.15527950310559 8 1

"735" 0.164596273291925 6 1

"736" 0.173913043478261 12 1

"737" 0.186335403726708 4 1

"738" 0.195652173913043 6 1

"739" 0.208074534161491 5 1

"740" 0.220496894409938 2 1

"741" 0.229813664596273 5 1

"742" 0.239130434782609 7 1

"743" 0.251552795031056 12 1

"744" 0.260869565217391 10 1

"745" 0.273291925465839 10 1

"746" 0.282608695652174 16 1

"747" 0.295031055900621 10 1

"748" 0.304347826086957 8 1

"749" 0.316770186335404 4 1

"750" 0.329192546583851 5 1

"751" 0.338509316770186 8 1

"752" 0.347826086956522 10 1

"753" 0.360248447204969 6 1

"754" 0.369565217391304 6 1

"755" 0.381987577639752 5 1

"756" 0.391304347826087 8 1

"757" 0.403726708074534 12 1

"758" 0.41304347826087 7 1

"759" 0.425465838509317 3 1

"760" 0.434782608695652 9 1

"761" 0.447204968944099 1 1

"762" 0.456521739130435 7 1

"763" 0.468944099378882 6 1

"764" 0.478260869565217 4 1

"765" 0.490683229813665 3 1

"766" 0.5 1 1

"767" 0.512422360248447 4 1

"768" 0.527950310559006 14 1

"769" 0.53416149068323 12 1

"770" 0.543478260869565 11 1

"771" 0.555900621118012 3 1

"772" 0.565217391304348 0 1

"773" 0.577639751552795 12 1

"774" 0.58695652173913 12 1

"775" 0.599378881987578 12 1

"776" 0.608695652173913 13 1

"777" 0.62111801242236 12 1

"778" 0.630434782608696 9 1

"779" 0.642857142857143 9 1

"780" 0.652173913043478 5 1

"781" 0.664596273291925 9 1

"782" 0.673913043478261 12 1

"783" 0.686335403726708 12 1

"784" 0.695652173913043 6 1

"785" 0.708074534161491 9 1

"786" 0.717391304347826 8 1

"787" 0.729813664596273 5 1

"788" 0.739130434782609 6 1

"789" 0.751552795031056 18 1

"790" 0.760869565217391 21 1

"791" 0.773291925465838 20 1

"792" 0.782608695652174 14 1

"793" 0.807453416149068 22 1

"794" 0.816770186335404 18 1

"795" 0.826086956521739 15 1

"796" 0.838509316770186 12 1

"797" 0.847826086956522 19 1

"798" 0.860248447204969 12 1

"799" 0.872670807453416 14 1

"800" 0.881987577639752 11 1

"801" 0.894409937888199 11 1

"802" 0.903726708074534 15 1

"803" 0.91304347826087 23 1

"804" 0.925465838509317 25 1

"805" 0.937888198757764 23 1

"806" 0.947204968944099 25 1

"807" 0.959627329192547 17 1

"808" 0.968944099378882 3 1

"809" 0.981366459627329 5 1

"810" 0.990683229813665 4 1

"811" 1 0 1

"812" 1.01242236024845 0 1

"813" 1.02173913043478 0 1

"814" 1.03726708074534 0 1

"815" 1.04968944099379 0 1

"816" 1.05590062111801 0 1

"817" 1.06521739130435 0 1

"818" 1.0776397515528 0 1

"819" 1.08695652173913 0 1

"820" 1.09937888198758 0 1

"821" 1.10869565217391 0 1

"822" 0.00343642611683849 0 1

"823" 0.0240549828178694 0 1

"824" 0.0378006872852234 2 1

"825" 0.0481099656357388 2 1

"826" 0.0618556701030928 4 1

"827" 0.0721649484536082 6 1

"828" 0.0859106529209622 5 1

"829" 0.0996563573883162 6 1

"830" 0.109965635738832 8 1

"831" 0.120274914089347 6 1

"832" 0.134020618556701 5 1

"833" 0.144329896907216 10 1

"834" 0.15807560137457 8 1

"835" 0.171821305841924 8 1

"836" 0.18213058419244 15 1

"837" 0.192439862542955 22 1

"838" 0.206185567010309 10 1

"839" 0.216494845360825 3 1

"840" 0.230240549828179 2 1

"841" 0.240549828178694 15 1

"842" 0.254295532646048 15 1

"843" 0.264604810996564 15 1

"844" 0.278350515463918 7 1

"845" 0.292096219931271 21 1

"846" 0.302405498281787 15 1

"847" 0.312714776632302 24 1

"848" 0.326460481099656 15 1

"849" 0.336769759450172 16 1

"850" 0.350515463917526 10 1

"851" 0.360824742268041 13 1

"852" 0.374570446735395 17 1

"853" 0.384879725085911 10 1

"854" 0.398625429553265 7 1

"855" 0.40893470790378 10 1

"856" 0.422680412371134 5 1

"857" 0.43298969072165 9 1

"858" 0.446735395189003 10 1

"859" 0.457044673539519 11 1

"860" 0.470790378006873 5 1

"861" 0.481099656357388 9 1

"862" 0.494845360824742 1 1

"863" 0.512027491408935 7 1

"864" 0.518900343642612 4 1

"865" 0.529209621993127 4 1

"866" 0.542955326460481 6 1

"867" 0.553264604810997 10 1

"868" 0.56701030927835 4 1

"869" 0.577319587628866 8 1

"870" 0.59106529209622 7 1

"871" 0.601374570446735 8 1

"872" 0.615120274914089 1 1

"873" 0.625429553264605 0 1

"874" 0.639175257731959 6 1

"875" 0.649484536082474 5 1

"876" 0.663230240549828 8 1

"877" 0.673539518900344 2 1

"878" 0.687285223367698 1 1

"879" 0.697594501718213 5 1

"880" 0.711340206185567 8 1

"881" 0.721649484536082 10 1

"882" 0.735395189003436 12 1

"883" 0.745704467353952 10 1

"884" 0.759450171821306 13 1

"885" 0.769759450171821 15 1

"886" 0.783505154639175 15 1

"887" 0.793814432989691 16 1

"888" 0.821305841924399 12 1

"889" 0.831615120274914 13 1

"890" 0.84192439862543 12 1

"891" 0.855670103092783 20 1

"892" 0.865979381443299 22 1

"893" 0.879725085910653 20 1

"894" 0.893470790378007 22 1

"895" 0.903780068728522 12 1

"896" 0.917525773195876 20 1

"897" 0.927835051546392 10 1

"898" 0.938144329896907 21 1

"899" 0.951890034364261 10 1

"900" 0.965635738831615 18 1

"901" 0.975945017182131 17 1

"902" 0.989690721649485 0 1

"903" 1 0 1

"904" 1.01374570446735 0 1

"905" 1.02405498281787 0 1

"906" 1.03436426116838 0 1

"907" 1.04810996563574 0 1

"908" 1.05841924398625 0 1

"909" 1.07560137457045 0 1

"910" 1.0893470790378 0 1

"911" 0.00384615384615385 0 1

"912" 0.0153846153846154 0 1

"913" 0.0269230769230769 0 1

"914" 0.0423076923076923 2 1

"915" 0.0538461538461538 5 1

"916" 0.0692307692307692 9 1

"917" 0.0807692307692308 0 1

"918" 0.0961538461538462 0 1

"919" 0.111538461538462 7 1

"920" 0.123076923076923 6 1

"921" 0.134615384615385 13 1

"922" 0.15 12 1

"923" 0.161538461538462 13 1

"924" 0.176923076923077 7 1

"925" 0.192307692307692 16 1

"926" 0.203846153846154 10 1

"927" 0.215384615384615 21 1

"928" 0.230769230769231 8 1

"929" 0.242307692307692 16 1

"930" 0.257692307692308 10 1

"931" 0.269230769230769 19 1

"932" 0.284615384615385 15 1

"933" 0.296153846153846 15 1

"934" 0.311538461538462 10 1

"935" 0.326923076923077 27 1

"936" 0.338461538461538 20 1

"937" 0.35 11 1

"938" 0.365384615384615 25 1

"939" 0.376923076923077 27 1

"940" 0.392307692307692 20 1

"941" 0.403846153846154 21 1

"942" 0.419230769230769 17 1

"943" 0.430769230769231 10 1

"944" 0.446153846153846 20 1

"945" 0.457692307692308 22 1

"946" 0.473076923076923 10 1

"947" 0.484615384615385 15 1

"948" 0.5 17 1

"949" 0.511538461538461 16 1

"950" 0.526923076923077 18 1

"951" 0.538461538461538 16 1

"952" 0.553846153846154 5 1

"953" 0.573076923076923 10 1

"954" 0.580769230769231 5 1

"955" 0.592307692307692 10 1

"956" 0.607692307692308 7 1

"957" 0.619230769230769 7 1

"958" 0.634615384615385 4 1

"959" 0.646153846153846 6 1

"960" 0.661538461538462 5 1

"961" 0.673076923076923 7 1

"962" 0.688461538461538 4 1

"963" 0.7 4 1

"964" 0.715384615384615 8 1

"965" 0.726923076923077 5 1

"966" 0.742307692307692 2 1

"967" 0.753846153846154 9 1

"968" 0.769230769230769 10 1

"969" 0.780769230769231 8 1

"970" 0.796153846153846 8 1

"971" 0.807692307692308 6 1

"972" 0.823076923076923 6 1

"973" 0.834615384615385 4 1

"974" 0.85 3 1

"975" 0.861538461538462 8 1

"976" 0.876923076923077 8 1

"977" 0.888461538461538 12 1

"978" 0.919230769230769 15 1

"979" 0.930769230769231 16 1

"980" 0.942307692307692 5 1

"981" 0.957692307692308 8 1

"982" 0.969230769230769 4 1

"983" 0.984615384615385 5 1

"984" 1 9 1

"985" 0.0072992700729927 0 1

"986" 0.0291970802919708 1 1

"987" 0.0583941605839416 5 1

"988" 0.0875912408759124 5 1

"989" 0.109489051094891 8 1

"990" 0.131386861313869 6 1

"991" 0.160583941605839 6 1

"992" 0.182481751824818 6 1

"993" 0.211678832116788 7 1

"994" 0.240875912408759 6 1

"995" 0.262773722627737 8 1

"996" 0.284671532846715 6 1

"997" 0.313868613138686 9 1

"998" 0.335766423357664 10 1

"999" 0.364963503649635 7 1

"1000" 0.386861313868613 6 1

"1001" 0.416058394160584 9 1

"1002" 0.437956204379562 15 1

"1003" 0.467153284671533 10 1

"1004" 0.496350364963504 6 1

"1005" 0.518248175182482 3 1

"1006" 0.54014598540146 14 1

"1007" 0.569343065693431 5 1

"1008" 0.591240875912409 9 1

"1009" 0.62043795620438 15 1

"1010" 0.642335766423358 19 1

"1011" 0.671532846715328 14 1

"1012" 0.693430656934307 15 1

"1013" 0.722627737226277 15 1

"1014" 0.744525547445255 19 1

"1015" 0.773722627737226 17 1

"1016" 0.795620437956204 15 1

"1017" 0.824817518248175 15 1

"1018" 0.846715328467153 12 1

"1019" 0.875912408759124 15 1

"1020" 0.897810218978102 14 1

"1021" 0.927007299270073 10 1

"1022" 0.963503649635037 0 1

"1023" 0.978102189781022 5 1

"1024" 1 0 1

"1025" 0.0075187969924812 0 1

"1026" 0.0902255639097744 0 1

"1027" 0.135338345864662 6 1

"1028" 0.195488721804511 0 1

"1029" 0.263157894736842 5 1

"1030" 0.293233082706767 8 1

"1031" 0.315789473684211 10 1

"1032" 0.345864661654135 8 1

"1033" 0.37593984962406 5 1

"1034" 0.398496240601504 6 1

"1035" 0.421052631578947 6 1

"1036" 0.451127819548872 2 1

"1037" 0.473684210526316 5 1

"1038" 0.503759398496241 6 1

"1039" 0.533834586466165 4 1

"1040" 0.556390977443609 10 1

"1041" 0.578947368421053 11 1

"1042" 0.609022556390977 3 1

"1043" 0.631578947368421 5 1

"1044" 0.661654135338346 8 1

"1045" 0.684210526315789 10 1

"1046" 0.714285714285714 7 1

"1047" 0.736842105263158 11 1

"1048" 0.766917293233083 5 1

"1049" 0.796992481203007 9 1

"1050" 0.819548872180451 10 1

"1051" 0.842105263157895 8 1

"1052" 0.87218045112782 8 1

"1053" 0.894736842105263 9 1

"1054" 0.924812030075188 5 1

"1055" 0.947368421052632 12 1

"1056" 0.977443609022556 9 1

"1057" 1 1 1

"1058" 1.03007518796992 0 1

"1059" 1.05263157894737 0 1

"1060" 1.08270676691729 0 1

"1061" 0.0140845070422535 0 1

"1062" 0.169014084507042 3 1

"1063" 0.253521126760563 0 1

"1064" 0.366197183098592 0 1

"1065" 0.394366197183099 0 1

"1066" 0.492957746478873 7 1

"1067" 0.549295774647887 9 1

"1068" 0.591549295774648 6 1

"1069" 0.647887323943662 4 1

"1070" 0.704225352112676 3 1

"1071" 0.746478873239437 2 1

"1072" 0.788732394366197 2 1

"1073" 0.845070422535211 1 1

"1074" 0.887323943661972 0 1

"1075" 0.943661971830986 0 1

"1076" 1 0 1

"1077" 1.04225352112676 0 1

"1078" 1.08450704225352 0 1

"1079" 1.14084507042254 0 1

"1080" 1.1830985915493 0 1

"1081" 1.23943661971831 0 1

"1082" 0.00980392156862745 0 1

"1083" 0.0392156862745098 0 1

"1084" 0.0784313725490196 2 1

"1085" 0.117647058823529 3 1

"1086" 0.147058823529412 6 1

"1087" 0.176470588235294 9 1

"1088" 0.215686274509804 6 1

"1089" 0.245098039215686 2 1

"1090" 0.284313725490196 8 1

"1091" 0.323529411764706 3 1

"1092" 0.352941176470588 10 1

"1093" 0.382352941176471 3 1

"1094" 0.42156862745098 10 1

"1095" 0.450980392156863 10 1

"1096" 0.490196078431373 8 1

"1097" 0.519607843137255 8 1

"1098" 0.558823529411765 15 1

"1099" 0.588235294117647 9 1

"1100" 0.627450980392157 0 1

"1101" 0.666666666666667 5 1

"1102" 0.696078431372549 8 1

"1103" 0.725490196078431 11 1

"1104" 0.764705882352941 5 1

"1105" 0.794117647058823 2 1

"1106" 0.833333333333333 5 1

"1107" 0.862745098039216 6 1

"1108" 0.901960784313726 0 1

"1109" 0.931372549019608 0 1

"1110" 0.970588235294118 3 1

"1111" 1 0 1

"1112" 0.00943396226415094 0 1

"1113" 0.0377358490566038 0 1

"1114" 0.0754716981132075 6 1

"1115" 0.113207547169811 9 1

"1116" 0.141509433962264 10 1

"1117" 0.169811320754717 9 1

"1118" 0.207547169811321 7 1

"1119" 0.235849056603774 4 1

"1120" 0.273584905660377 2 1

"1121" 0.311320754716981 10 1

"1122" 0.339622641509434 8 1

"1123" 0.367924528301887 10 1

"1124" 0.405660377358491 14 1

"1125" 0.433962264150943 12 1

"1126" 0.471698113207547 17 1

"1127" 0.5 10 1

"1128" 0.537735849056604 0 1

"1129" 0.566037735849057 4 1

"1130" 0.60377358490566 3 1

"1131" 0.641509433962264 0 1

"1132" 0.669811320754717 10 1

"1133" 0.69811320754717 6 1

"1134" 0.735849056603774 7 1

"1135" 0.764150943396226 7 1

"1136" 0.80188679245283 6 1

"1137" 0.830188679245283 6 1

"1138" 0.867924528301887 3 1

"1139" 0.89622641509434 5 1

"1140" 0.933962264150943 5 1

"1141" 0.962264150943396 6 1

"1142" 1 2 1

"1143" 1.02830188679245 0 1

"1144" 1.06603773584906 0 1

"1145" 0.010989010989011 0 1

"1146" 0.0879120879120879 5 1

"1147" 0.153846153846154 0 1

"1148" 0.186813186813187 7 1

"1149" 0.230769230769231 6 1

"1150" 0.263736263736264 8 1

"1151" 0.307692307692308 6 1

"1152" 0.351648351648352 9 1

"1153" 0.384615384615385 11 1

"1154" 0.417582417582418 11 1

"1155" 0.461538461538462 3 1

"1156" 0.494505494505495 10 1

"1157" 0.538461538461538 15 1

"1158" 0.582417582417582 30 1

"1159" 0.615384615384615 8 1

"1160" 0.648351648351648 29 1

"1161" 0.692307692307692 20 1

"1162" 0.725274725274725 19 1

"1163" 0.769230769230769 10 1

"1164" 0.802197802197802 8 1

"1165" 0.846153846153846 5 1

"1166" 0.879120879120879 7 1

"1167" 0.923076923076923 3 1

"1168" 0.967032967032967 1 1

"1169" 1 0 1

"1170" 1.03296703296703 0 1

"1171" 1.07692307692308 0 1

"1172" 1.10989010989011 0 1

"1173" 1.15384615384615 0 1

"1174" 1.18681318681319 0 1

"1175" 0.010989010989011 0 1

"1176" 0.032967032967033 0 1

"1177" 0.0769230769230769 2 1

"1178" 0.10989010989011 5 1

"1179" 0.153846153846154 5 1

"1180" 0.186813186813187 7 1

"1181" 0.230769230769231 10 1

"1182" 0.274725274725275 8 1

"1183" 0.307692307692308 5 1

"1184" 0.340659340659341 9 1

"1185" 0.384615384615385 11 1

"1186" 0.417582417582418 9 1

"1187" 0.461538461538462 5 1

"1188" 0.505494505494505 8 1

"1189" 0.538461538461538 8 1

"1190" 0.571428571428571 5 1

"1191" 0.615384615384615 7 1

"1192" 0.648351648351648 15 1

"1193" 0.692307692307692 17 1

"1194" 0.725274725274725 16 1

"1195" 0.769230769230769 10 1

"1196" 0.802197802197802 19 1

"1197" 0.846153846153846 20 1

"1198" 0.89010989010989 15 1

"1199" 0.923076923076923 15 1

"1200" 0.956043956043956 14 1

"1201" 1 5 1

"1202" 1.03296703296703 1 1

"1203" 1.07692307692308 0 1

"1204" 1.10989010989011 0 1

"1205" 1.15384615384615 0 1

"1206" 1.18681318681319 0 1

"1207" 1.23076923076923 0 1

"1208" 1.26373626373626 0 1

"1209" 1.30769230769231 0 1

"1210" 1.34065934065934 0 1

"1211" 1.38461538461538 0 1

"1212" 0.00840336134453781 0 1

"1213" 0.0588235294117647 0 1

"1214" 0.126050420168067 0 1

"1215" 0.142857142857143 0 1

"1216" 0.201680672268908 8 1

"1217" 0.235294117647059 10 1

"1218" 0.260504201680672 13 1

"1219" 0.294117647058824 8 1

"1220" 0.327731092436975 2 1

"1221" 0.352941176470588 4 1

"1222" 0.378151260504202 4 1

"1223" 0.411764705882353 5 1

"1224" 0.436974789915966 1 1

"1225" 0.470588235294118 2 1

"1226" 0.504201680672269 5 1

"1227" 0.529411764705882 3 1

"1228" 0.554621848739496 5 1

"1229" 0.588235294117647 3 1

"1230" 0.613445378151261 0 1

"1231" 0.647058823529412 0 1

"1232" 0.672268907563025 4 1

"1233" 0.705882352941177 10 1

"1234" 0.73109243697479 4 1

"1235" 0.764705882352941 0 1

"1236" 0.798319327731092 6 1

"1237" 0.823529411764706 5 1

"1238" 0.848739495798319 4 1

"1239" 0.882352941176471 0 1

"1240" 0.907563025210084 0 1

"1241" 0.941176470588235 0 1

"1242" 0.966386554621849 8 1

"1243" 1 0 1

"1244" 0.0121951219512195 0 1

"1245" 0.0853658536585366 0 1

"1246" 0.158536585365854 2 1

"1247" 0.24390243902439 0 1

"1248" 0.268292682926829 0 1

"1249" 0.353658536585366 12 1

"1250" 0.402439024390244 12 1

"1251" 0.439024390243902 10 1

"1252" 0.48780487804878 16 1

"1253" 0.536585365853659 5 1

"1254" 0.573170731707317 13 1

"1255" 0.609756097560976 5 1

"1256" 0.658536585365854 4 1

"1257" 0.695121951219512 2 1

"1258" 0.74390243902439 4 1

"1259" 0.792682926829268 1 1

"1260" 0.829268292682927 0 1

"1261" 0.865853658536585 0 1

"1262" 0.914634146341463 0 1

"1263" 0.951219512195122 0 1

"1264" 1 0 1

"1265" 1.03658536585366 0 1

"1266" 1.08536585365854 0 1

"1267" 0.00952380952380952 0 1

"1268" 0.0761904761904762 0 1

"1269" 0.0952380952380952 0 1

"1270" 0.133333333333333 2 1

"1271" 0.161904761904762 7 1

"1272" 0.2 7 1

"1273" 0.228571428571429 5 1

"1274" 0.266666666666667 10 1

"1275" 0.304761904761905 3 1

"1276" 0.333333333333333 9 1

"1277" 0.361904761904762 7 1

"1278" 0.4 10 1

"1279" 0.428571428571429 9 1

"1280" 0.466666666666667 11 1

"1281" 0.504761904761905 11 1

"1282" 0.533333333333333 6 1

"1283" 0.561904761904762 6 1

"1284" 0.6 5 1

"1285" 0.628571428571429 9 1

"1286" 0.666666666666667 15 1

"1287" 0.695238095238095 8 1

"1288" 0.733333333333333 4 1

"1289" 0.761904761904762 2 1

"1290" 0.8 5 1

"1291" 0.838095238095238 6 1

"1292" 0.866666666666667 1 1

"1293" 0.895238095238095 0 1

"1294" 0.933333333333333 0 1

"1295" 0.961904761904762 0 1

"1296" 1 0 1

"1297" 0.0123456790123457 0 1

"1298" 0.0864197530864197 2 1

"1299" 0.234567901234568 0 1

"1300" 0.320987654320988 0 1

"1301" 0.345679012345679 0 1

"1302" 0.432098765432099 10 1

"1303" 0.481481481481481 12 1

"1304" 0.518518518518518 18 1

"1305" 0.567901234567901 15 1

"1306" 0.617283950617284 19 1

"1307" 0.654320987654321 10 1

"1308" 0.691358024691358 17 1

"1309" 0.740740740740741 15 1

"1310" 0.777777777777778 10 1

"1311" 0.827160493827161 10 1

"1312" 0.876543209876543 20 1

"1313" 0.91358024691358 16 1

"1314" 0.950617283950617 36 1

"1315" 1 18 1

"1316" 1.03703703703704 8 1

"1317" 1.08641975308642 0 1

"1318" 1.12345679012346 0 1

"1319" 1.17283950617284 0 1

"1320" 1.20987654320988 0 1

"1321" 1.25925925925926 0 1

"1322" 1.30864197530864 0 1

"1323" 1.34567901234568 0 1

"1324" 1.38271604938272 0 1

"1325" 0.0123456790123457 0 1

"1326" 0.0864197530864197 1 1

"1327" 0.234567901234568 0 1

"1328" 0.320987654320988 0 1

"1329" 0.345679012345679 0 1

"1330" 0.432098765432099 7 1

"1331" 0.481481481481481 10 1

"1332" 0.518518518518518 8 1

"1333" 0.567901234567901 10 1

"1334" 0.617283950617284 14 1

"1335" 0.654320987654321 12 1

"1336" 0.691358024691358 12 1

"1337" 0.740740740740741 15 1

"1338" 0.777777777777778 10 1

"1339" 0.827160493827161 7 1

"1340" 0.876543209876543 6 1

"1341" 0.91358024691358 7 1

"1342" 0.950617283950617 3 1

"1343" 1 0 1

"1344" 1.03703703703704 0 1

"1345" 1.08641975308642 0 1

"1346" 1.12345679012346 0 1

"1347" 1.17283950617284 0 1

"1348" 1.20987654320988 0 1

"1349" 1.25925925925926 0 1

"1350" 0.005 0 1

"1351" 0.035 1 1

"1352" 0.065 0 1

"1353" 0.095 0 1

"1354" 0.13 0 1

"1355" 0.195 10 1

"1356" 0.21 12 1

"1357" 0.23 13 1

"1358" 0.25 10 1

"1359" 0.265 8 1

"1360" 0.28 14 1

"1361" 0.3 17 1

"1362" 0.315 11 1

"1363" 0.335 10 1

"1364" 0.355 18 1

"1365" 0.37 10 1

"1366" 0.385 14 1

"1367" 0.405 14 1

"1368" 0.42 11 1

"1369" 0.44 8 1

"1370" 0.455 12 1

"1371" 0.475 10 1

"1372" 0.49 16 1

"1373" 0.51 12 1

"1374" 0.53 18 1

"1375" 0.545 15 1

"1376" 0.56 11 1

"1377" 0.58 6 1

"1378" 0.595 10 1

"1379" 0.615 8 1

"1380" 0.63 15 1

"1381" 0.65 10 1

"1382" 0.665 5 1

"1383" 0.685 4 1

"1384" 0.7 3 1

"1385" 0.72 7 1

"1386" 0.735 7 1

"1387" 0.755 10 1

"1388" 0.77 13 1

"1389" 0.79 7 1

"1390" 0.805 3 1

"1391" 0.825 1 1

"1392" 0.85 11 1

"1393" 0.86 4 1

"1394" 0.875 3 1

"1395" 0.895 10 1

"1396" 0.91 10 1

"1397" 0.93 2 1

"1398" 0.945 0 1

"1399" 0.965 5 1

"1400" 0.98 7 1

"1401" 1 4 1

"1402" 1.015 0 1

"1403" 0.0062111801242236 0 1

"1404" 0.0496894409937888 2 1

"1405" 0.062111801242236 0 1

"1406" 0.105590062111801 10 1

"1407" 0.130434782608696 10 1

"1408" 0.149068322981366 8 1

"1409" 0.173913043478261 8 1

"1410" 0.198757763975155 5 1

"1411" 0.217391304347826 12 1

"1412" 0.236024844720497 8 1

"1413" 0.260869565217391 17 1

"1414" 0.279503105590062 23 1

"1415" 0.304347826086957 20 1

"1416" 0.329192546583851 18 1

"1417" 0.347826086956522 15 1

"1418" 0.366459627329193 23 1

"1419" 0.391304347826087 15 1

"1420" 0.409937888198758 15 1

"1421" 0.434782608695652 20 1

"1422" 0.453416149068323 24 1

"1423" 0.478260869565217 30 1

"1424" 0.496894409937888 31 1

"1425" 0.521739130434783 25 1

"1426" 0.546583850931677 18 1

"1427" 0.565217391304348 17 1

"1428" 0.583850931677019 25 1

"1429" 0.608695652173913 20 1

"1430" 0.627329192546584 26 1

"1431" 0.652173913043478 30 1

"1432" 0.670807453416149 31 1

"1433" 0.695652173913043 23 1

"1434" 0.714285714285714 20 1

"1435" 0.739130434782609 15 1

"1436" 0.757763975155279 25 1

"1437" 0.782608695652174 10 1

"1438" 0.801242236024845 21 1

"1439" 0.826086956521739 17 1

"1440" 0.84472049689441 14 1

"1441" 0.869565217391304 20 1

"1442" 0.888198757763975 18 1

"1443" 0.91304347826087 8 1

"1444" 0.944099378881988 9 1

"1445" 0.956521739130435 5 1

"1446" 0.975155279503106 0 1

"1447" 1 0 1

"1448" 0.00763358778625954 0 1

"1449" 0.0534351145038168 0 1

"1450" 0.099236641221374 0 1

"1451" 0.152671755725191 0 1

"1452" 0.16793893129771 0 1

"1453" 0.221374045801527 6 1

"1454" 0.251908396946565 6 1

"1455" 0.274809160305344 8 1

"1456" 0.305343511450382 5 1

"1457" 0.33587786259542 3 1

"1458" 0.358778625954198 0 1

"1459" 0.381679389312977 0 1

"1460" 0.412213740458015 1 1

"1461" 0.435114503816794 0 1

"1462" 0.465648854961832 4 1

"1463" 0.49618320610687 0 1

"1464" 0.519083969465649 6 1

"1465" 0.541984732824427 7 1

"1466" 0.572519083969466 8 1

"1467" 0.595419847328244 2 1

"1468" 0.625954198473282 7 1

"1469" 0.648854961832061 5 1

"1470" 0.679389312977099 10 1

"1471" 0.702290076335878 0 1

"1472" 0.732824427480916 6 1

"1473" 0.763358778625954 2 1

"1474" 0.786259541984733 5 1

"1475" 0.809160305343511 1 1

"1476" 0.83969465648855 0 1

"1477" 0.862595419847328 1 1

"1478" 0.893129770992366 0 1

"1479" 0.916030534351145 1 1

"1480" 0.946564885496183 7 1

"1481" 0.969465648854962 0 1

"1482" 1 0 1

"1483" 0.00714285714285714 0 1

"1484" 0.0214285714285714 0 1

"1485" 0.05 1 1

"1486" 0.0714285714285714 2 1

"1487" 0.1 5 1

"1488" 0.121428571428571 9 1

"1489" 0.15 8 1

"1490" 0.178571428571429 8 1

"1491" 0.2 7 1

"1492" 0.221428571428571 9 1

"1493" 0.25 6 1

"1494" 0.271428571428571 11 1

"1495" 0.3 10 1

"1496" 0.328571428571429 5 1

"1497" 0.35 11 1

"1498" 0.371428571428571 17 1

"1499" 0.4 14 1

"1500" 0.421428571428571 7 1

"1501" 0.45 5 1

"1502" 0.471428571428571 12 1

"1503" 0.5 10 1

"1504" 0.521428571428571 13 1

"1505" 0.55 5 1

"1506" 0.578571428571429 11 1

"1507" 0.6 12 1

"1508" 0.621428571428571 12 1

"1509" 0.65 15 1

"1510" 0.671428571428571 14 1

"1511" 0.7 5 1

"1512" 0.721428571428571 11 1

"1513" 0.75 18 1

"1514" 0.771428571428571 10 1

"1515" 0.8 10 1

"1516" 0.821428571428571 9 1

"1517" 0.85 9 1

"1518" 0.871428571428571 17 1

"1519" 0.9 20 1

"1520" 0.921428571428571 18 1

"1521" 0.95 6 1

"1522" 0.971428571428571 6 1

"1523" 1 0 1

"1524" 0.0105263157894737 0 1

"1525" 0.136842105263158 4 1

"1526" 0.2 0 1

"1527" 0.294736842105263 0 1

"1528" 0.368421052631579 12 1

"1529" 0.410526315789474 12 1

"1530" 0.442105263157895 15 1

"1531" 0.484210526315789 18 1

"1532" 0.526315789473684 20 1

"1533" 0.557894736842105 15 1

"1534" 0.589473684210526 24 1

"1535" 0.631578947368421 22 1

"1536" 0.663157894736842 26 1

"1537" 0.705263157894737 6 1

"1538" 0.747368421052632 13 1

"1539" 0.778947368421053 15 1

"1540" 0.810526315789474 35 1

"1541" 0.852631578947368 30 1

"1542" 0.884210526315789 38 1

"1543" 0.926315789473684 25 1

"1544" 0.957894736842105 27 1

"1545" 1 12 1

"1546" 1.03157894736842 6 1

"1547" 1.07368421052632 0 1

"1548" 1.11578947368421 0 1

"1549" 1.14736842105263 0 1

"1550" 1.17894736842105 0 1

"1551" 1.22105263157895 0 1

"1552" 1.25263157894737 0 1

"1553" 1.29473684210526 0 1

"1554" 0.0112359550561798 0 1

"1555" 0.0786516853932584 1 1

"1556" 0.146067415730337 0 1

"1557" 0.247191011235955 0 1

"1558" 0.325842696629214 12 1

"1559" 0.370786516853933 8 1

"1560" 0.404494382022472 15 1

"1561" 0.449438202247191 15 1

"1562" 0.49438202247191 13 1

"1563" 0.528089887640449 12 1

"1564" 0.561797752808989 10 1

"1565" 0.606741573033708 6 1

"1566" 0.640449438202247 3 1

"1567" 0.685393258426966 8 1

"1568" 0.730337078651685 2 1

"1569" 0.764044943820225 2 1

"1570" 0.797752808988764 3 1

"1571" 0.842696629213483 1 1

"1572" 0.876404494382023 2 1

"1573" 0.921348314606742 0 1

"1574" 0.955056179775281 1 1

"1575" 1 0 1

"1576" 1.03370786516854 0 1

"1577" 1.07865168539326 0 1

"1578" 0.00909090909090909 0 1

"1579" 0.0636363636363636 6 1

"1580" 0.118181818181818 0 1

"1581" 0.2 0 1

"1582" 0.263636363636364 8 1

"1583" 0.3 15 1

"1584" 0.327272727272727 13 1

"1585" 0.363636363636364 12 1

"1586" 0.4 15 1

"1587" 0.427272727272727 10 1

"1588" 0.454545454545455 17 1

"1589" 0.490909090909091 10 1

"1590" 0.518181818181818 4 1

"1591" 0.554545454545455 1 1

"1592" 0.590909090909091 1 1

"1593" 0.618181818181818 0 1

"1594" 0.645454545454546 0 1

"1595" 0.681818181818182 1 1

"1596" 0.709090909090909 1 1

"1597" 0.745454545454545 3 1

"1598" 0.772727272727273 3 1

"1599" 0.809090909090909 6 1

"1600" 0.836363636363636 6 1

"1601" 0.872727272727273 2 1

"1602" 0.909090909090909 5 1

"1603" 0.936363636363636 4 1

"1604" 0.963636363636364 0 1

"1605" 1 0 1

"1606" 1.02727272727273 0 1

"1607" 1.06363636363636 0 1

"1608" 1.09090909090909 0 1

"1609" 0.00813008130081301 0 1

"1610" 0.032520325203252 0 1

"1611" 0.0650406504065041 2 1

"1612" 0.0894308943089431 5 1

"1613" 0.121951219512195 9 1

"1614" 0.154471544715447 7 1

"1615" 0.178861788617886 10 1

"1616" 0.203252032520325 6 1

"1617" 0.235772357723577 4 1

"1618" 0.260162601626016 6 1

"1619" 0.292682926829268 3 1

"1620" 0.32520325203252 4 1

"1621" 0.349593495934959 3 1

"1622" 0.373983739837398 0 1

"1623" 0.40650406504065 8 1

"1624" 0.430894308943089 6 1

"1625" 0.463414634146341 7 1

"1626" 0.48780487804878 7 1

"1627" 0.520325203252033 7 1

"1628" 0.544715447154472 8 1

"1629" 0.577235772357724 5 1

"1630" 0.609756097560976 1 1

"1631" 0.634146341463415 6 1

"1632" 0.658536585365854 4 1

"1633" 0.691056910569106 8 1

"1634" 0.715447154471545 7 1

"1635" 0.747967479674797 10 1

"1636" 0.772357723577236 8 1

"1637" 0.804878048780488 5 1

"1638" 0.829268292682927 3 1

"1639" 0.861788617886179 5 1

"1640" 0.886178861788618 5 1

"1641" 0.91869918699187 5 1

"1642" 0.943089430894309 5 1

"1643" 0.975609756097561 2 1

"1644" 1 0 1

"1645" 1.03252032520325 0 1

"1646" 0.00598802395209581 0 1

"1647" 0.0479041916167665 7 1

"1648" 0.0838323353293413 0 1

"1649" 0.137724550898204 0 1

"1650" 0.179640718562874 9 1

"1651" 0.203592814371257 10 1

"1652" 0.221556886227545 12 1

"1653" 0.245508982035928 11 1

"1654" 0.269461077844311 14 1

"1655" 0.287425149700599 13 1

"1656" 0.305389221556886 14 1

"1657" 0.329341317365269 11 1

"1658" 0.347305389221557 13 1

"1659" 0.37125748502994 5 1

"1660" 0.395209580838323 10 1

"1661" 0.413173652694611 11 1

"1662" 0.431137724550898 3 1

"1663" 0.455089820359281 5 1

"1664" 0.473053892215569 13 1

"1665" 0.497005988023952 10 1

"1666" 0.514970059880239 10 1

"1667" 0.538922155688623 10 1

"1668" 0.55688622754491 14 1

"1669" 0.580838323353293 13 1

"1670" 0.604790419161677 9 1

"1671" 0.622754491017964 5 1

"1672" 0.640718562874252 14 1

"1673" 0.664670658682635 10 1

"1674" 0.682634730538922 8 1

"1675" 0.706586826347305 11 1

"1676" 0.724550898203593 5 1

"1677" 0.748502994011976 5 1

"1678" 0.766467065868264 5 1

"1679" 0.790419161676647 6 1

"1680" 0.808383233532934 5 1

"1681" 0.832335329341317 4 1

"1682" 0.850299401197605 2 1

"1683" 0.874251497005988 10 1

"1684" 0.892215568862275 5 1

"1685" 0.916167664670659 5 1

"1686" 0.934131736526946 0 1

"1687" 0.958083832335329 0 1

"1688" 0.988023952095808 0 1

"1689" 1 0 1

"1690" 0.0113636363636364 0 1

"1691" 0.147727272727273 4 1

"1692" 0.215909090909091 0 1

"1693" 0.318181818181818 0 1

"1694" 0.397727272727273 10 1

"1695" 0.443181818181818 13 1

"1696" 0.477272727272727 17 1

"1697" 0.522727272727273 16 1

"1698" 0.568181818181818 15 1

"1699" 0.602272727272727 14 1

"1700" 0.636363636363636 10 1

"1701" 0.681818181818182 10 1

"1702" 0.715909090909091 13 1

"1703" 0.761363636363636 16 1

"1704" 0.806818181818182 14 1

"1705" 0.840909090909091 10 1

"1706" 0.875 6 1

"1707" 0.920454545454545 12 1

"1708" 0.954545454545455 7 1

"1709" 1 8 1

"1710" 1.03409090909091 8 1

"1711" 1.07954545454545 1 1

"1712" 1.11363636363636 0 1

"1713" 1.15909090909091 0 1

"1714" 1.20454545454545 0 1

"1715" 1.23863636363636 0 1

"1716" 1.27272727272727 0 1

"1717" 1.31818181818182 0 1

"1718" 0.00704225352112676 0 1

"1719" 0.0563380281690141 0 1

"1720" 0.0985915492957746 0 1

"1721" 0.169014084507042 0 1

"1722" 0.211267605633803 3 1

"1723" 0.23943661971831 0 1

"1724" 0.26056338028169 5 1

"1725" 0.288732394366197 3 1

"1726" 0.316901408450704 3 1

"1727" 0.338028169014085 5 1

"1728" 0.359154929577465 3 1

"1729" 0.387323943661972 5 1

"1730" 0.408450704225352 4 1

"1731" 0.436619718309859 7 1

"1732" 0.464788732394366 2 1

"1733" 0.485915492957746 2 1

"1734" 0.507042253521127 4 1

"1735" 0.535211267605634 0 1

"1736" 0.556338028169014 6 1

"1737" 0.584507042253521 3 1

"1738" 0.605633802816901 5 1

"1739" 0.633802816901408 5 1

"1740" 0.654929577464789 2 1

"1741" 0.683098591549296 2 1

"1742" 0.711267605633803 2 1

"1743" 0.732394366197183 2 1

"1744" 0.753521126760563 1 1

"1745" 0.78169014084507 5 1

"1746" 0.802816901408451 4 1

"1747" 0.830985915492958 5 1

"1748" 0.852112676056338 2 1

"1749" 0.880281690140845 0 1

"1750" 0.901408450704225 0 1

"1751" 0.929577464788732 0 1

"1752" 0.950704225352113 0 1

"1753" 0.97887323943662 0 1

"1754" 1 0 1

"1755" 0.00840336134453781 0 1

"1756" 0.0840336134453782 0 1

"1757" 0.117647058823529 1 1

"1758" 0.142857142857143 2 1

"1759" 0.176470588235294 5 1

"1760" 0.201680672268908 5 1

"1761" 0.235294117647059 6 1

"1762" 0.26890756302521 5 1

"1763" 0.294117647058824 10 1

"1764" 0.319327731092437 4 1

"1765" 0.352941176470588 10 1

"1766" 0.378151260504202 16 1

"1767" 0.411764705882353 10 1

"1768" 0.445378151260504 12 1

"1769" 0.470588235294118 10 1

"1770" 0.495798319327731 15 1

"1771" 0.529411764705882 11 1

"1772" 0.554621848739496 12 1

"1773" 0.588235294117647 13 1

"1774" 0.613445378151261 12 1

"1775" 0.647058823529412 8 1

"1776" 0.672268907563025 15 1

"1777" 0.705882352941177 10 1

"1778" 0.739495798319328 25 1

"1779" 0.764705882352941 10 1

"1780" 0.789915966386555 23 1

"1781" 0.823529411764706 12 1

"1782" 0.848739495798319 8 1

"1783" 0.882352941176471 12 1

"1784" 0.907563025210084 10 1

"1785" 0.941176470588235 17 1

"1786" 0.966386554621849 20 1

"1787" 1 7 1

"1788" 1.02521008403361 3 1

"1789" 1.05882352941176 0 1

"1790" 1.08403361344538 0 1

"1791" 1.11764705882353 0 1

"1792" 1.14285714285714 0 1

"1793" 1.17647058823529 0 1

"1794" 1.20168067226891 0 1

"1795" 1.23529411764706 0 1

"1796" 1.27731092436975 0 1

"1797" 0.00757575757575758 0 1

"1798" 0.0606060606060606 3 1

"1799" 0.106060606060606 0 1

"1800" 0.174242424242424 0 1

"1801" 0.204545454545455 0 1

"1802" 0.227272727272727 10 1

"1803" 0.257575757575758 0 1

"1804" 0.28030303030303 5 1

"1805" 0.310606060606061 3 1

"1806" 0.340909090909091 9 1

"1807" 0.363636363636364 5 1

"1808" 0.386363636363636 9 1

"1809" 0.416666666666667 10 1

"1810" 0.439393939393939 4 1

"1811" 0.46969696969697 2 1

"1812" 0.5 9 1

"1813" 0.522727272727273 5 1

"1814" 0.545454545454545 8 1

"1815" 0.575757575757576 3 1

"1816" 0.598484848484849 5 1

"1817" 0.628787878787879 7 1

"1818" 0.651515151515151 5 1

"1819" 0.681818181818182 7 1

"1820" 0.704545454545455 7 1

"1821" 0.734848484848485 10 1

"1822" 0.765151515151515 0 1

"1823" 0.787878787878788 5 1

"1824" 0.810606060606061 5 1

"1825" 0.840909090909091 2 1

"1826" 0.863636363636364 4 1

"1827" 0.893939393939394 3 1

"1828" 0.916666666666667 1 1

"1829" 0.946969696969697 1 1

"1830" 0.96969696969697 1 1

"1831" 1 0 1

"1832" 1.02272727272727 0 1

"1833" 1.0530303030303 0 1

"1834" 1.07575757575758 0 1

"1835" 1.10606060606061 0 1

"1836" 0.00684931506849315 0 1

"1837" 0.0547945205479452 2 1

"1838" 0.10958904109589 0 1

"1839" 0.157534246575342 0 1

"1840" 0.205479452054795 10 1

"1841" 0.232876712328767 10 1

"1842" 0.253424657534247 6 1

"1843" 0.280821917808219 5 1

"1844" 0.308219178082192 7 1

"1845" 0.328767123287671 5 1

"1846" 0.349315068493151 9 1

"1847" 0.376712328767123 5 1

"1848" 0.397260273972603 6 1

"1849" 0.424657534246575 3 1

"1850" 0.452054794520548 3 1

"1851" 0.472602739726027 6 1

"1852" 0.493150684931507 5 1

"1853" 0.520547945205479 5 1

"1854" 0.541095890410959 8 1

"1855" 0.568493150684932 12 1

"1856" 0.589041095890411 9 1

"1857" 0.616438356164384 7 1

"1858" 0.636986301369863 6 1

"1859" 0.664383561643836 5 1

"1860" 0.691780821917808 6 1

"1861" 0.712328767123288 3 1

"1862" 0.732876712328767 1 1

"1863" 0.76027397260274 3 1

"1864" 0.780821917808219 10 1

"1865" 0.808219178082192 10 1

"1866" 0.828767123287671 4 1

"1867" 0.856164383561644 9 1

"1868" 0.876712328767123 5 1

"1869" 0.904109589041096 10 1

"1870" 0.924657534246575 7 1

"1871" 0.952054794520548 6 1

"1872" 0.972602739726027 4 1

"1873" 1 8 1

"1874" 1 0 1

"1875" 0.00531914893617021 0 1

"1876" 0.0478723404255319 2 1

"1877" 0.0851063829787234 0 1

"1878" 0.122340425531915 5 1

"1879" 0.143617021276596 5 1

"1880" 0.159574468085106 6 1

"1881" 0.180851063829787 6 1

"1882" 0.202127659574468 14 1

"1883" 0.218085106382979 10 1

"1884" 0.234042553191489 9 1

"1885" 0.25531914893617 0 1

"1886" 0.271276595744681 11 1

"1887" 0.292553191489362 6 1

"1888" 0.313829787234043 8 1

"1889" 0.329787234042553 7 1

"1890" 0.345744680851064 8 1

"1891" 0.367021276595745 10 1

"1892" 0.382978723404255 8 1

"1893" 0.404255319148936 11 1

"1894" 0.420212765957447 10 1

"1895" 0.441489361702128 15 1

"1896" 0.457446808510638 6 1

"1897" 0.478723404255319 15 1

"1898" 0.5 0 1

"1899" 0.515957446808511 10 1

"1900" 0.531914893617021 8 1

"1901" 0.553191489361702 10 1

"1902" 0.569148936170213 13 1

"1903" 0.590425531914894 12 1

"1904" 0.606382978723404 2 1

"1905" 0.627659574468085 1 1

"1906" 0.643617021276596 4 1

"1907" 0.664893617021277 6 1

"1908" 0.680851063829787 2 1

"1909" 0.702127659574468 4 1

"1910" 0.718085106382979 1 1

"1911" 0.73936170212766 8 1

"1912" 0.75531914893617 5 1

"1913" 0.776595744680851 0 1

"1914" 0.792553191489362 0 1

"1915" 0.813829787234043 0 1

"1916" 0.840425531914894 9 1

"1917" 0.851063829787234 11 1

"1918" 0.867021276595745 10 1

"1919" 0.888297872340426 0 1

"1920" 0.904255319148936 1 1

"1921" 0.925531914893617 0 1

"1922" 0.941489361702128 1 1

"1923" 0.962765957446808 2 1

"1924" 0.978723404255319 0 1

"1925" 1 0 1

"1926" 0.00719424460431655 0 1

"1927" 0.0575539568345324 3 1

"1928" 0.115107913669065 0 1

"1929" 0.165467625899281 0 1

"1930" 0.215827338129496 8 1

"1931" 0.244604316546763 6 1

"1932" 0.266187050359712 5 1

"1933" 0.294964028776978 0 1

"1934" 0.323741007194245 2 1

"1935" 0.345323741007194 5 1

"1936" 0.366906474820144 4 1

"1937" 0.39568345323741 4 1

"1938" 0.41726618705036 5 1

"1939" 0.446043165467626 2 1

"1940" 0.474820143884892 6 1

"1941" 0.496402877697842 3 1

"1942" 0.517985611510791 1 1

"1943" 0.546762589928058 3 1

"1944" 0.568345323741007 4 1

"1945" 0.597122302158273 4 1

"1946" 0.618705035971223 4 1

"1947" 0.647482014388489 5 1

"1948" 0.669064748201439 4 1

"1949" 0.697841726618705 8 1

"1950" 0.726618705035971 3 1

"1951" 0.748201438848921 4 1

"1952" 0.76978417266187 2 1

"1953" 0.798561151079137 4 1

"1954" 0.820143884892086 2 1

"1955" 0.848920863309353 2 1

"1956" 0.870503597122302 3 1

"1957" 0.899280575539568 2 1

"1958" 0.920863309352518 2 1

"1959" 0.949640287769784 2 1

"1960" 0.971223021582734 0 1

"1961" 1 0 1

"1962" 0.00719424460431655 0 1

"1963" 0.0647482014388489 2 1

"1964" 0.115107913669065 0 1

"1965" 0.165467625899281 5 1

"1966" 0.194244604316547 5 1

"1967" 0.215827338129496 3 1

"1968" 0.244604316546763 3 1

"1969" 0.273381294964029 0 1

"1970" 0.294964028776978 4 1

"1971" 0.316546762589928 3 1

"1972" 0.345323741007194 5 1

"1973" 0.366906474820144 6 1

"1974" 0.39568345323741 2 1

"1975" 0.424460431654676 5 1

"1976" 0.446043165467626 3 1

"1977" 0.467625899280576 9 1

"1978" 0.496402877697842 5 1

"1979" 0.517985611510791 8 1

"1980" 0.546762589928058 3 1

"1981" 0.568345323741007 5 1

"1982" 0.597122302158273 5 1

"1983" 0.618705035971223 5 1

"1984" 0.647482014388489 10 1

"1985" 0.676258992805755 6 1

"1986" 0.697841726618705 1 1

"1987" 0.719424460431655 4 1

"1988" 0.748201438848921 6 1

"1989" 0.76978417266187 5 1

"1990" 0.798561151079137 6 1

"1991" 0.820143884892086 4 1

"1992" 0.848920863309353 3 1

"1993" 0.870503597122302 6 1

"1994" 0.899280575539568 2 1

"1995" 0.920863309352518 7 1

"1996" 0.949640287769784 3 1

"1997" 0.971223021582734 3 1

"1998" 1 0 1

"1999" 1.02158273381295 0 1

"2000" 1.05035971223022 0 1

"2001" 1.07194244604317 0 1

"2002" 1.10071942446043 0 1

"2003" 1.13669064748201 0 1

"2004" 0.00884955752212389 0 1

"2005" 0.0442477876106195 0 1

"2006" 0.079646017699115 4 1

"2007" 0.106194690265487 7 1

"2008" 0.132743362831858 3 1

"2009" 0.168141592920354 5 1

"2010" 0.194690265486726 6 1

"2011" 0.230088495575221 5 1

"2012" 0.265486725663717 6 1

"2013" 0.292035398230089 10 1

"2014" 0.31858407079646 8 1

"2015" 0.353982300884956 8 1

"2016" 0.380530973451327 10 1

"2017" 0.415929203539823 13 1

"2018" 0.442477876106195 6 1

"2019" 0.47787610619469 3 1

"2020" 0.504424778761062 6 1

"2021" 0.539823008849557 5 1

"2022" 0.575221238938053 7 1

"2023" 0.601769911504425 5 1

"2024" 0.628318584070796 2 1

"2025" 0.663716814159292 5 1

"2026" 0.690265486725664 1 1

"2027" 0.725663716814159 2 1

"2028" 0.752212389380531 4 1

"2029" 0.787610619469027 5 1

"2030" 0.814159292035398 4 1

"2031" 0.849557522123894 5 1

"2032" 0.876106194690266 0 1

"2033" 0.911504424778761 1 1

"2034" 0.938053097345133 0 1

"2035" 0.973451327433628 1 1

"2036" 1 1 1

"2037" 1.0353982300885 0 1

"2038" 0.00746268656716418 0 1

"2039" 0.0671641791044776 1 1

"2040" 0.082089552238806 0 1

"2041" 0.111940298507463 0 1

"2042" 0.134328358208955 3 1

"2043" 0.164179104477612 4 1

"2044" 0.186567164179104 8 1

"2045" 0.216417910447761 3 1

"2046" 0.246268656716418 3 1

"2047" 0.26865671641791 5 1

"2048" 0.291044776119403 0 1

"2049" 0.32089552238806 2 1

"2050" 0.343283582089552 0 1

"2051" 0.373134328358209 2 1

"2052" 0.402985074626866 4 1

"2053" 0.425373134328358 0 1

"2054" 0.447761194029851 0 1

"2055" 0.477611940298507 1 1

"2056" 0.5 0 1

"2057" 0.529850746268657 3 1

"2058" 0.552238805970149 2 1

"2059" 0.582089552238806 4 1

"2060" 0.604477611940298 2 1

"2061" 0.634328358208955 3 1

"2062" 0.664179104477612 3 1

"2063" 0.686567164179104 3 1

"2064" 0.708955223880597 1 1

"2065" 0.738805970149254 3 1

"2066" 0.761194029850746 3 1

"2067" 0.791044776119403 4 1

"2068" 0.813432835820896 3 1

"2069" 0.843283582089552 2 1

"2070" 0.865671641791045 0 1

"2071" 0.895522388059702 1 1

"2072" 0.917910447761194 0 1

"2073" 0.947761194029851 2 1

"2074" 0.970149253731343 0 1

"2075" 1 0 1

"2076" 0.00628930817610063 0 1

"2077" 0.0314465408805031 0 1

"2078" 0.050314465408805 1 1

"2079" 0.0754716981132075 0 1

"2080" 0.0943396226415094 6 1

"2081" 0.119496855345912 5 1

"2082" 0.144654088050314 8 1

"2083" 0.163522012578616 5 1

"2084" 0.182389937106918 5 1

"2085" 0.207547169811321 9 1

"2086" 0.226415094339623 10 1

"2087" 0.251572327044025 7 1

"2088" 0.276729559748428 11 1

"2089" 0.29559748427673 5 1

"2090" 0.314465408805031 11 1

"2091" 0.339622641509434 20 1

"2092" 0.358490566037736 26 1

"2093" 0.383647798742138 12 1

"2094" 0.40251572327044 15 1

"2095" 0.427672955974843 20 1

"2096" 0.446540880503145 12 1

"2097" 0.471698113207547 8 1

"2098" 0.49685534591195 16 1

"2099" 0.515723270440252 17 1

"2100" 0.534591194968553 13 1

"2101" 0.559748427672956 15 1

"2102" 0.578616352201258 12 1

"2103" 0.60377358490566 15 1

"2104" 0.622641509433962 14 1

"2105" 0.647798742138365 17 1

"2106" 0.666666666666667 12 1

"2107" 0.691823899371069 15 1

"2108" 0.710691823899371 15 1

"2109" 0.735849056603774 10 1

"2110" 0.754716981132076 12 1

"2111" 0.779874213836478 10 1

"2112" 0.79874213836478 12 1

"2113" 0.823899371069182 10 1

"2114" 0.842767295597484 8 1

"2115" 0.867924528301887 3 1

"2116" 0.89937106918239 0 1

"2117" 0.911949685534591 5 1

"2118" 0.930817610062893 0 1

"2119" 0.955974842767296 0 1

"2120" 0.974842767295597 0 1

"2121" 1 0 1

"2122" 1.0188679245283 0 1

"2123" 1.0440251572327 0 1

"2124" 1.06289308176101 0 1

"2125" 1.08805031446541 0 1

"2126" 0.0108695652173913 0 1

"2127" 0.0434782608695652 0 1

"2128" 0.0869565217391304 8 1

"2129" 0.119565217391304 10 1

"2130" 0.16304347826087 8 1

"2131" 0.206521739130435 9 1

"2132" 0.239130434782609 3 1

"2133" 0.271739130434783 8 1

"2134" 0.315217391304348 6 1

"2135" 0.347826086956522 5 1

"2136" 0.391304347826087 8 1

"2137" 0.434782608695652 10 1

"2138" 0.467391304347826 11 1

"2139" 0.5 3 1

"2140" 0.543478260869565 6 1

"2141" 0.576086956521739 7 1

"2142" 0.619565217391304 6 1

"2143" 0.652173913043478 0 1

"2144" 0.695652173913043 3 1

"2145" 0.728260869565217 0 1

"2146" 0.771739130434783 1 1

"2147" 0.815217391304348 0 1

"2148" 0.847826086956522 1 1

"2149" 0.880434782608696 0 1

"2150" 0.923913043478261 0 1

"2151" 0.956521739130435 0 1

"2152" 1 0 1

"2153" 1.03260869565217 0 1

"2154" 1.07608695652174 0 1

"2155" 1.10869565217391 0 1

"2156" 1.15217391304348 0 1

"2157" 0.0175438596491228 0 1

"2158" 0.0701754385964912 0 1

"2159" 0.140350877192982 3 1

"2160" 0.210526315789474 6 1

"2161" 0.263157894736842 0 1

"2162" 0.315789473684211 3 1

"2163" 0.385964912280702 0 1

"2164" 0.43859649122807 5 1

"2165" 0.508771929824561 10 1

"2166" 0.578947368421053 8 1

"2167" 0.631578947368421 3 1

"2168" 0.684210526315789 8 1

"2169" 0.754385964912281 5 1

"2170" 0.807017543859649 5 1

"2171" 0.87719298245614 2 1

"2172" 0.929824561403509 0 1

"2173" 1 0 1

"2174" 1.05263157894737 0 1

"2175" 1.12280701754386 0 1

"2176" 1.19298245614035 0 1

"2177" 1.24561403508772 0 1

"2178" 0.0105263157894737 0 1

"2179" 0.0421052631578947 0 1

"2180" 0.0842105263157895 6 1

"2181" 0.126315789473684 7 1

"2182" 0.157894736842105 3 1

"2183" 0.189473684210526 3 1

"2184" 0.231578947368421 3 1

"2185" 0.263157894736842 2 1

"2186" 0.305263157894737 3 1

"2187" 0.336842105263158 1 1

"2188" 0.378947368421053 2 1

"2189" 0.410526315789474 3 1

"2190" 0.452631578947368 0 1

"2191" 0.494736842105263 2 1

"2192" 0.526315789473684 0 1

"2193" 0.557894736842105 1 1

"2194" 0.6 3 1

"2195" 0.631578947368421 2 1

"2196" 0.673684210526316 3 1

"2197" 0.705263157894737 5 1

"2198" 0.747368421052632 4 1

"2199" 0.778947368421053 5 1

"2200" 0.821052631578947 5 1

"2201" 0.852631578947368 8 1

"2202" 0.894736842105263 0 1

"2203" 0.926315789473684 1 1

"2204" 0.968421052631579 1 1

"2205" 1 0 1

"2206" 0.00675675675675676 0 1

"2207" 0.027027027027027 1 1

"2208" 0.0540540540540541 5 1

"2209" 0.0810810810810811 5 1

"2210" 0.101351351351351 4 1

"2211" 0.121621621621622 3 1

"2212" 0.148648648648649 4 1

"2213" 0.168918918918919 6 1

"2214" 0.195945945945946 0 1

"2215" 0.216216216216216 4 1

"2216" 0.243243243243243 4 1

"2217" 0.263513513513513 5 1

"2218" 0.290540540540541 3 1

"2219" 0.317567567567568 3 1

"2220" 0.337837837837838 2 1

"2221" 0.358108108108108 5 1

"2222" 0.385135135135135 5 1

"2223" 0.405405405405405 6 1

"2224" 0.432432432432432 6 1

"2225" 0.452702702702703 3 1

"2226" 0.47972972972973 2 1

"2227" 0.5 3 1

"2228" 0.527027027027027 3 1

"2229" 0.547297297297297 5 1

"2230" 0.574324324324324 4 1

"2231" 0.594594594594595 4 1

"2232" 0.621621621621622 5 1

"2233" 0.641891891891892 3 1

"2234" 0.668918918918919 3 1

"2235" 0.689189189189189 3 1

"2236" 0.716216216216216 3 1

"2237" 0.75 2 1

"2238" 0.763513513513513 5 1

"2239" 0.783783783783784 7 1

"2240" 0.810810810810811 11 1

"2241" 0.831081081081081 9 1

"2242" 0.858108108108108 5 1

"2243" 0.878378378378378 7 1

"2244" 0.905405405405405 9 1

"2245" 0.925675675675676 9 1

"2246" 0.952702702702703 5 1

"2247" 0.972972972972973 4 1

"2248" 1 4 1

"2249" 1.02027027027027 0 1

"2250" 1.0472972972973 0 1

"2251" 1.06756756756757 0 1

"2252" 1.09459459459459 0 1

"2253" 1.11486486486486 0 1

"2254" 1.14189189189189 0 1

"2255" 1.16216216216216 0 1

"2256" 1.18918918918919 0 1

"2257" 0.02 0 1

"2258" 0.08 1 1

"2259" 0.16 6 1

"2260" 0.24 8 1

"2261" 0.3 8 1

"2262" 0.36 5 1

"2263" 0.44 0 1

"2264" 0.5 0 1

"2265" 0.58 5 1

"2266" 0.64 8 1

"2267" 0.72 8 1

"2268" 0.78 7 1

"2269" 0.86 3 1

"2270" 0.94 0 1

"2271" 1 0 1

"2272" 0.00694444444444444 0 2

"2273" 0.0277777777777778 0 2

"2274" 0.0555555555555556 0 2

"2275" 0.0763888888888889 0 2

"2276" 0.104166666666667 1 2

"2277" 0.125 3 2

"2278" 0.152777777777778 5 2

"2279" 0.173611111111111 3 2

"2280" 0.201388888888889 1 2

"2281" 0.222222222222222 3 2

"2282" 0.25 2 2

"2283" 0.270833333333333 3 2

"2284" 0.298611111111111 0 2

"2285" 0.319444444444444 0 2

"2286" 0.347222222222222 0 2

"2287" 0.368055555555556 3 2

"2288" 0.423611111111111 4 2

"2289" 0.444444444444444 4 2

"2290" 0.465277777777778 5 2

"2291" 0.493055555555556 8 2

"2292" 0.513888888888889 7 2

"2293" 0.541666666666667 6 2

"2294" 0.569444444444444 7 2

"2295" 0.590277777777778 5 2

"2296" 0.618055555555556 9 2

"2297" 0.638888888888889 7 2

"2298" 0.659722222222222 11 2

"2299" 0.6875 5 2

"2300" 0.715277777777778 1 2

"2301" 0.736111111111111 0 2

"2302" 0.763888888888889 7 2

"2303" 0.784722222222222 8 2

"2304" 0.8125 5 2

"2305" 0.833333333333333 5 2

"2306" 0.854166666666667 6 2

"2307" 0.881944444444444 9 2

"2308" 0.902777777777778 9 2

"2309" 0.9375 0 2

"2310" 0.965277777777778 0 2

"2311" 0.979166666666667 0 2

"2312" 1 0 2

"2313" 1.02777777777778 0 2

"2314" 1.04861111111111 0 2

"2315" 1.07638888888889 0 2

"2316" 1.09722222222222 0 2

"2317" 0.00884955752212389 0 2

"2318" 0.0353982300884956 0 2

"2319" 0.0707964601769911 0 2

"2320" 0.0973451327433628 0 2

"2321" 0.132743362831858 1 2

"2322" 0.15929203539823 1 2

"2323" 0.194690265486726 1 2

"2324" 0.221238938053097 3 2

"2325" 0.292035398230089 1 2

"2326" 0.31858407079646 2 2

"2327" 0.345132743362832 3 2

"2328" 0.380530973451327 3 2

"2329" 0.407079646017699 3 2

"2330" 0.442477876106195 5 2

"2331" 0.47787610619469 1 2

"2332" 0.504424778761062 4 2

"2333" 0.539823008849557 3 2

"2334" 0.566371681415929 0 2

"2335" 0.592920353982301 5 2

"2336" 0.628318584070796 11 2

"2337" 0.663716814159292 10 2

"2338" 0.690265486725664 6 2

"2339" 0.725663716814159 9 2

"2340" 0.752212389380531 7 2

"2341" 0.787610619469027 16 2

"2342" 0.814159292035398 7 2

"2343" 0.84070796460177 8 2

"2344" 0.876106194690266 8 2

"2345" 0.902654867256637 9 2

"2346" 0.946902654867257 2 2

"2347" 1 0 2

"2348" 1.02654867256637 0 2

"2349" 1.06194690265487 0 2

"2350" 1.08849557522124 0 2

"2351" 0.0133333333333333 0 2

"2352" 0.0533333333333333 0 2

"2353" 0.106666666666667 0 2

"2354" 0.146666666666667 0 2

"2355" 0.2 0 2

"2356" 0.24 0 2

"2357" 0.293333333333333 0 2

"2358" 0.333333333333333 1 2

"2359" 0.386666666666667 2 2

"2360" 0.426666666666667 2 2

"2361" 0.48 6 2

"2362" 0.52 6 2

"2363" 0.626666666666667 9 2

"2364" 0.666666666666667 9 2

"2365" 0.706666666666667 12 2

"2366" 0.76 8 2

"2367" 0.8 11 2

"2368" 0.853333333333333 10 2

"2369" 0.906666666666667 7 2

"2370" 0.946666666666667 9 2

"2371" 1 0 2

"2372" 0.0232558139534884 0 2

"2373" 0.209302325581395 0 2

"2374" 0.27906976744186 0 2

"2375" 0.348837209302326 1 2

"2376" 0.441860465116279 8 2

"2377" 0.511627906976744 4 2

"2378" 0.604651162790698 3 2

"2379" 0.697674418604651 0 2

"2380" 0.767441860465116 0 2

"2381" 0.86046511627907 0 2

"2382" 0.930232558139535 0 2

"2383" 1 0 2

"2384" 1.09302325581395 0 2

"2385" 0.00854700854700855 0 2

"2386" 0.0427350427350427 0 2

"2387" 0.0683760683760684 0 2

"2388" 0.102564102564103 6 2

"2389" 0.128205128205128 3 2

"2390" 0.162393162393162 5 2

"2391" 0.188034188034188 2 2

"2392" 0.222222222222222 4 2

"2393" 0.247863247863248 2 2

"2394" 0.282051282051282 4 2

"2395" 0.307692307692308 8 2

"2396" 0.341880341880342 9 2

"2397" 0.367521367521368 4 2

"2398" 0.401709401709402 4 2

"2399" 0.427350427350427 6 2

"2400" 0.461538461538462 9 2

"2401" 0.487179487179487 10 2

"2402" 0.521367521367521 10 2

"2403" 0.547008547008547 9 2

"2404" 0.581196581196581 7 2

"2405" 0.606837606837607 15 2

"2406" 0.675213675213675 15 2

"2407" 0.700854700854701 25 2

"2408" 0.726495726495726 15 2

"2409" 0.760683760683761 10 2

"2410" 0.786324786324786 22 2

"2411" 0.82051282051282 14 2

"2412" 0.854700854700855 15 2

"2413" 0.88034188034188 25 2

"2414" 0.914529914529915 18 2

"2415" 0.94017094017094 13 2

"2416" 0.965811965811966 7 2

"2417" 1 0 2

"2418" 1.03418803418803 0 2

"2419" 1.05982905982906 0 2

"2420" 1.09401709401709 0 2

"2421" 1.11965811965812 0 2

"2422" 1.15384615384615 0 2

"2423" 1.17948717948718 0 2

"2424" 1.20512820512821 0 2

"2425" 1.23931623931624 0 2

"2426" 1.26495726495726 0 2

"2427" 0.00675675675675676 0 2

"2428" 0.0540540540540541 0 2

"2429" 0.0810810810810811 4 2

"2430" 0.101351351351351 5 2

"2431" 0.128378378378378 3 2

"2432" 0.148648648648649 5 2

"2433" 0.175675675675676 6 2

"2434" 0.195945945945946 8 2

"2435" 0.222972972972973 8 2

"2436" 0.243243243243243 11 2

"2437" 0.27027027027027 12 2

"2438" 0.290540540540541 10 2

"2439" 0.317567567567568 10 2

"2440" 0.337837837837838 8 2

"2441" 0.364864864864865 5 2

"2442" 0.385135135135135 9 2

"2443" 0.412162162162162 7 2

"2444" 0.432432432432432 8 2

"2445" 0.459459459459459 13 2

"2446" 0.47972972972973 4 2

"2447" 0.533783783783784 10 2

"2448" 0.554054054054054 15 2

"2449" 0.574324324324324 14 2

"2450" 0.601351351351351 12 2

"2451" 0.621621621621622 12 2

"2452" 0.648648648648649 12 2

"2453" 0.675675675675676 9 2

"2454" 0.695945945945946 11 2

"2455" 0.722972972972973 12 2

"2456" 0.743243243243243 13 2

"2457" 0.763513513513513 12 2

"2458" 0.790540540540541 11 2

"2459" 0.817567567567568 14 2

"2460" 0.837837837837838 10 2

"2461" 0.864864864864865 18 2

"2462" 0.885135135135135 14 2

"2463" 0.912162162162162 13 2

"2464" 0.932432432432432 15 2

"2465" 0.952702702702703 0 2

"2466" 0.97972972972973 0 2

"2467" 1 0 2

"2468" 1.03378378378378 0 2

"2469" 1.06081081081081 0 2

"2470" 1.07432432432432 0 2

"2471" 0.00480769230769231 0 2

"2472" 0.0192307692307692 0 2

"2473" 0.0384615384615385 1 2

"2474" 0.0528846153846154 1 2

"2475" 0.0721153846153846 0 2

"2476" 0.0865384615384615 0 2

"2477" 0.105769230769231 0 2

"2478" 0.120192307692308 0 2

"2479" 0.139423076923077 0 2

"2480" 0.153846153846154 0 2

"2481" 0.173076923076923 0 2

"2482" 0.1875 1 2

"2483" 0.206730769230769 0 2

"2484" 0.221153846153846 0 2

"2485" 0.240384615384615 0 2

"2486" 0.254807692307692 0 2

"2487" 0.274038461538462 0 2

"2488" 0.288461538461538 0 2

"2489" 0.326923076923077 1 2

"2490" 0.341346153846154 3 2

"2491" 0.355769230769231 5 2

"2492" 0.375 8 2

"2493" 0.389423076923077 7 2

"2494" 0.408653846153846 8 2

"2495" 0.427884615384615 13 2

"2496" 0.442307692307692 8 2

"2497" 0.461538461538462 13 2

"2498" 0.475961538461538 11 2

"2499" 0.490384615384615 12 2

"2500" 0.509615384615385 13 2

"2501" 0.528846153846154 14 2

"2502" 0.543269230769231 8 2

"2503" 0.5625 17 2

"2504" 0.576923076923077 16 2

"2505" 0.596153846153846 14 2

"2506" 0.610576923076923 10 2

"2507" 0.625 35 2

"2508" 0.644230769230769 27 2

"2509" 0.658653846153846 28 2

"2510" 0.682692307692308 25 2

"2511" 0.711538461538462 40 2

"2512" 0.725961538461538 28 2

"2513" 0.745192307692308 34 2

"2514" 0.759615384615385 30 2

"2515" 0.778846153846154 29 2

"2516" 0.793269230769231 21 2

"2517" 0.817307692307692 24 2

"2518" 0.831730769230769 17 2

"2519" 0.846153846153846 18 2

"2520" 0.865384615384615 15 2

"2521" 4.50961538461539 16 2

"2522" 0.0106382978723404 0 2

"2523" 0.0531914893617021 0 2

"2524" 0.0851063829787234 0 2

"2525" 0.170212765957447 1 2

"2526" 0.202127659574468 0 2

"2527" 0.234042553191489 0 2

"2528" 0.276595744680851 0 2

"2529" 0.308510638297872 0 2

"2530" 0.351063829787234 0 2

"2531" 0.393617021276596 0 2

"2532" 0.425531914893617 0 2

"2533" 0.468085106382979 0 2

"2534" 0.5 0 2

"2535" 0.531914893617021 0 2

"2536" 0.574468085106383 4 2

"2537" 0.617021276595745 8 2

"2538" 0.648936170212766 10 2

"2539" 0.691489361702128 6 2

"2540" 0.723404255319149 8 2

"2541" 0.765957446808511 6 2

"2542" 0.797872340425532 13 2

"2543" 0.829787234042553 14 2

"2544" 0.872340425531915 9 2

"2545" 0.904255319148936 15 2

"2546" 0.957446808510638 2 2

"2547" 1 0 2

"2548" 1 0 2

"2549" 1.02127659574468 0 2

"2550" 1.0531914893617 0 2

"2551" 1.09574468085106 0 2

"2552" 1.12765957446809 0 2

"2553" 1.17021276595745 0 2

"2554" 0.00617283950617284 0 2

"2555" 0.0308641975308642 0 2

"2556" 0.0493827160493827 0 2

"2557" 0.0740740740740741 1 2

"2558" 0.0925925925925926 3 2

"2559" 0.117283950617284 6 2

"2560" 0.135802469135802 5 2

"2561" 0.160493827160494 6 2

"2562" 0.179012345679012 7 2

"2563" 0.203703703703704 12 2

"2564" 0.222222222222222 9 2

"2565" 0.246913580246914 10 2

"2566" 0.265432098765432 10 2

"2567" 0.290123456790123 12 2

"2568" 0.308641975308642 17 2

"2569" 0.333333333333333 13 2

"2570" 0.351851851851852 12 2

"2571" 0.376543209876543 12 2

"2572" 0.395061728395062 20 2

"2573" 0.419753086419753 17 2

"2574" 0.438271604938272 18 2

"2575" 0.487654320987654 20 2

"2576" 0.506172839506173 14 2

"2577" 0.524691358024691 15 2

"2578" 0.549382716049383 5 2

"2579" 0.567901234567901 4 2

"2580" 0.592592592592593 3 2

"2581" 0.617283950617284 4 2

"2582" 0.635802469135803 11 2

"2583" 0.660493827160494 6 2

"2584" 0.679012345679012 3 2

"2585" 0.697530864197531 12 2

"2586" 0.722222222222222 14 2

"2587" 0.746913580246914 22 2

"2588" 0.765432098765432 25 2

"2589" 0.790123456790123 19 2

"2590" 0.808641975308642 10 2

"2591" 0.833333333333333 24 2

"2592" 0.851851851851852 15 2

"2593" 0.87037037037037 25 2

"2594" 0.895061728395062 27 2

"2595" 0.91358024691358 11 2

"2596" 0.944444444444444 4 2

"2597" 0.969135802469136 14 2

"2598" 0.969135802469136 0 2

"2599" 0.981481481481482 0 2

"2600" 1 0 2

"2601" 1.02469135802469 0 2

"2602" 1.04320987654321 0 2

"2603" 1.0679012345679 0 2

"2604" 0.00442477876106195 0 2

"2605" 0.0221238938053097 0 2

"2606" 0.0353982300884956 0 2

"2607" 0.0530973451327434 1 2

"2608" 0.0663716814159292 7 2

"2609" 0.084070796460177 9 2

"2610" 0.0973451327433628 6 2

"2611" 0.115044247787611 6 2

"2612" 0.128318584070796 5 2

"2613" 0.146017699115044 9 2

"2614" 0.15929203539823 12 2

"2615" 0.176991150442478 11 2

"2616" 0.190265486725664 8 2

"2617" 0.207964601769911 9 2

"2618" 0.221238938053097 15 2

"2619" 0.238938053097345 18 2

"2620" 0.252212389380531 13 2

"2621" 0.269911504424779 14 2

"2622" 0.283185840707965 19 2

"2623" 0.300884955752212 16 2

"2624" 0.314159292035398 17 2

"2625" 0.331858407079646 18 2

"2626" 0.345132743362832 21 2

"2627" 0.380530973451327 9 2

"2628" 0.393805309734513 19 2

"2629" 0.407079646017699 18 2

"2630" 0.424778761061947 15 2

"2631" 0.438053097345133 13 2

"2632" 0.455752212389381 5 2

"2633" 0.473451327433628 1 2

"2634" 0.486725663716814 16 2

"2635" 0.504424778761062 22 2

"2636" 0.517699115044248 17 2

"2637" 0.530973451327434 21 2

"2638" 0.548672566371681 14 2

"2639" 0.566371681415929 19 2

"2640" 0.579646017699115 15 2

"2641" 0.597345132743363 7 2

"2642" 0.610619469026549 14 2

"2643" 0.628318584070796 13 2

"2644" 0.641592920353982 30 2

"2645" 0.654867256637168 19 2

"2646" 0.672566371681416 17 2

"2647" 0.685840707964602 41 2

"2648" 0.707964601769911 30 2

"2649" 0.725663716814159 45 2

"2650" 0.734513274336283 41 2

"2651" 0.747787610619469 26 2

"2652" 0.765486725663717 31 2

"2653" 0.778761061946903 34 2

"2654" 0.79646017699115 29 2

"2655" 0.809734513274336 26 2

"2656" 0.831858407079646 18 2

"2657" 0.845132743362832 22 2

"2658" 0.858407079646018 24 2

"2659" 0.876106194690266 21 2

"2660" 1 20 2

"2661" 0.0140845070422535 0 2

"2662" 0.0704225352112676 0 2

"2663" 0.112676056338028 0 2

"2664" 0.169014084507042 1 2

"2665" 0.211267605633803 4 2

"2666" 0.267605633802817 6 2

"2667" 0.309859154929577 5 2

"2668" 0.366197183098592 6 2

"2669" 0.408450704225352 7 2

"2670" 0.464788732394366 2 2

"2671" 0.507042253521127 9 2

"2672" 0.563380281690141 13 2

"2673" 0.605633802816901 9 2

"2674" 0.661971830985915 9 2

"2675" 0.704225352112676 11 2

"2676" 0.76056338028169 10 2

"2677" 0.802816901408451 6 2

"2678" 0.859154929577465 4 2

"2679" 0.901408450704225 2 2

"2680" 0.957746478873239 1 2

"2681" 1 0 2

"2682" 1.05633802816901 0 2

"2683" 1.09859154929577 0 2

"2684" 1.2112676056338 0 2

"2685" 1.25352112676056 0 2

"2686" 1.29577464788732 0 2

"2687" 1.35211267605634 0 2

"2688" 0.00636942675159236 0 2

"2689" 0.0318471337579618 1 2

"2690" 0.0509554140127389 5 2

"2691" 0.0764331210191083 6 2

"2692" 0.0955414012738854 5 2

"2693" 0.121019108280255 6 2

"2694" 0.140127388535032 6 2

"2695" 0.165605095541401 7 2

"2696" 0.184713375796178 9 2

"2697" 0.210191082802548 11 2

"2698" 0.229299363057325 8 2

"2699" 0.254777070063694 7 2

"2700" 0.273885350318471 9 2

"2701" 0.299363057324841 10 2

"2702" 0.318471337579618 12 2

"2703" 0.343949044585987 13 2

"2704" 0.363057324840764 14 2

"2705" 0.388535031847134 9 2

"2706" 0.407643312101911 19 2

"2707" 0.43312101910828 21 2

"2708" 0.452229299363057 18 2

"2709" 0.503184713375796 14 2

"2710" 0.522292993630573 22 2

"2711" 0.54140127388535 10 2

"2712" 0.56687898089172 18 2

"2713" 0.585987261146497 21 2

"2714" 0.611464968152866 25 2

"2715" 0.636942675159236 29 2

"2716" 0.656050955414013 28 2

"2717" 0.681528662420382 15 2

"2718" 0.700636942675159 21 2

"2719" 0.719745222929936 24 2

"2720" 0.745222929936306 21 2

"2721" 0.770700636942675 23 2

"2722" 0.789808917197452 20 2

"2723" 0.815286624203822 27 2

"2724" 0.834394904458599 15 2

"2725" 0.859872611464968 26 2

"2726" 0.878980891719745 30 2

"2727" 0.898089171974522 10 2

"2728" 0.923566878980892 8 2

"2729" 0.942675159235669 0 2

"2730" 0.974522292993631 0 2

"2731" 1 0 2

"2732" 1.01273885350318 0 2

"2733" 1.03184713375796 0 2

"2734" 1.05732484076433 0 2

"2735" 1.07643312101911 0 2

"2736" 1.10191082802548 0 2

"2737" 0.00543478260869565 0 2

"2738" 0.0217391304347826 0 2

"2739" 0.0434782608695652 1 2

"2740" 0.0597826086956522 4 2

"2741" 0.0815217391304348 5 2

"2742" 0.0978260869565217 8 2

"2743" 0.119565217391304 5 2

"2744" 0.135869565217391 4 2

"2745" 0.157608695652174 4 2

"2746" 0.173913043478261 6 2

"2747" 0.195652173913043 8 2

"2748" 0.21195652173913 6 2

"2749" 0.233695652173913 4 2

"2750" 0.25 9 2

"2751" 0.271739130434783 13 2

"2752" 0.28804347826087 11 2

"2753" 0.309782608695652 16 2

"2754" 0.326086956521739 12 2

"2755" 0.347826086956522 14 2

"2756" 0.364130434782609 13 2

"2757" 0.385869565217391 13 2

"2758" 0.402173913043478 7 2

"2759" 0.445652173913043 13 2

"2760" 0.46195652173913 20 2

"2761" 0.478260869565217 21 2

"2762" 0.5 21 2

"2763" 0.516304347826087 28 2

"2764" 0.53804347826087 26 2

"2765" 0.559782608695652 27 2

"2766" 0.576086956521739 32 2

"2767" 0.597826086956522 20 2

"2768" 0.614130434782609 21 2

"2769" 0.630434782608696 26 2

"2770" 0.652173913043478 25 2

"2771" 0.673913043478261 25 2

"2772" 0.71195652173913 33 2

"2773" 0.728260869565217 20 2

"2774" 0.75 24 2

"2775" 0.766304347826087 20 2

"2776" 0.782608695652174 15 2

"2777" 0.804347826086957 10 2

"2778" 0.820652173913043 41 2

"2779" 0.847826086956522 44 2

"2780" 0.869565217391304 42 2

"2781" 0.880434782608696 37 2

"2782" 0.896739130434783 40 2

"2783" 0.918478260869565 25 2

"2784" 0.934782608695652 22 2

"2785" 0.956521739130435 33 2

"2786" 0.972826086956522 24 2

"2787" 1 0 2

"2788" 0.00819672131147541 0 2

"2789" 0.0491803278688525 0 2

"2790" 0.0737704918032787 1 2

"2791" 0.10655737704918 7 2

"2792" 0.131147540983607 4 2

"2793" 0.163934426229508 8 2

"2794" 0.188524590163934 9 2

"2795" 0.221311475409836 4 2

"2796" 0.245901639344262 12 2

"2797" 0.278688524590164 9 2

"2798" 0.30327868852459 9 2

"2799" 0.336065573770492 10 2

"2800" 0.360655737704918 11 2

"2801" 0.39344262295082 12 2

"2802" 0.418032786885246 18 2

"2803" 0.450819672131148 17 2

"2804" 0.475409836065574 17 2

"2805" 0.508196721311475 18 2

"2806" 0.532786885245902 19 2

"2807" 0.565573770491803 25 2

"2808" 0.590163934426229 32 2

"2809" 0.655737704918033 13 2

"2810" 0.680327868852459 25 2

"2811" 0.704918032786885 24 2

"2812" 0.737704918032787 25 2

"2813" 0.762295081967213 28 2

"2814" 0.795081967213115 23 2

"2815" 0.827868852459016 31 2

"2816" 0.852459016393443 30 2

"2817" 0.885245901639344 20 2

"2818" 0.909836065573771 13 2

"2819" 0.934426229508197 9 2

"2820" 0.967213114754098 0 2

"2821" 1 0 2

"2822" 1.02459016393443 0 2

"2823" 1.05737704918033 0 2

"2824" 1.08196721311475 0 2

"2825" 1.11475409836066 0 2

"2826" 1.13934426229508 0 2

"2827" 1.16393442622951 0 2

"2828" 1.19672131147541 0 2

"2829" 0.00523560209424084 0 2

"2830" 0.0261780104712042 0 2

"2831" 0.0418848167539267 0 2

"2832" 0.06282722513089 1 2

"2833" 0.0785340314136126 7 2

"2834" 0.0994764397905759 5 2

"2835" 0.115183246073298 7 2

"2836" 0.136125654450262 6 2

"2837" 0.151832460732984 6 2

"2838" 0.193717277486911 9 2

"2839" 0.209424083769634 7 2

"2840" 0.225130890052356 9 2

"2841" 0.246073298429319 7 2

"2842" 0.261780104712042 12 2

"2843" 0.282722513089005 14 2

"2844" 0.303664921465969 11 2

"2845" 0.319371727748691 20 2

"2846" 0.340314136125654 12 2

"2847" 0.356020942408377 15 2

"2848" 0.371727748691099 19 2

"2849" 0.392670157068063 6 2

"2850" 0.413612565445026 19 2

"2851" 0.429319371727749 20 2

"2852" 0.450261780104712 24 2

"2853" 0.465968586387435 15 2

"2854" 0.486910994764398 20 2

"2855" 0.50261780104712 15 2

"2856" 0.518324607329843 22 2

"2857" 0.539267015706806 12 2

"2858" 0.554973821989529 14 2

"2859" 0.581151832460733 10 2

"2860" 0.602094240837696 24 2

"2861" 0.612565445026178 20 2

"2862" 0.628272251308901 15 2

"2863" 0.649214659685864 24 2

"2864" 0.664921465968586 14 2

"2865" 0.68586387434555 14 2

"2866" 0.701570680628272 16 2

"2867" 0.727748691099476 18 2

"2868" 0.743455497382199 12 2

"2869" 0.759162303664921 13 2

"2870" 0.780104712041885 16 2

"2871" 0.926701570680628 21 2

"2872" 1 14 2

"2873" 0.0108695652173913 0 2

"2874" 0.0434782608695652 0 2

"2875" 0.0869565217391304 5 2

"2876" 0.119565217391304 7 2

"2877" 0.16304347826087 7 2

"2878" 0.195652173913043 8 2

"2879" 0.239130434782609 6 2

"2880" 0.16304347826087 9 2

"2881" 0.315217391304348 11 2

"2882" 0.347826086956522 12 2

"2883" 0.391304347826087 12 2

"2884" 0.423913043478261 13 2

"2885" 0.467391304347826 17 2

"2886" 0.5 19 2

"2887" 0.543478260869565 10 2

"2888" 0.576086956521739 19 2

"2889" 0.619565217391304 27 2

"2890" 0.652173913043478 14 2

"2891" 0.739130434782609 22 2

"2892" 0.771739130434783 17 2

"2893" 0.804347826086957 14 2

"2894" 0.847826086956522 13 2

"2895" 0.880434782608696 6 2

"2896" 0.923913043478261 0 2

"2897" 0.967391304347826 0 2

"2898" 1 0 2

"2899" 1.04347826086957 0 2

"2900" 1.07608695652174 0 2

"2901" 1.10869565217391 0 2

"2902" 1.15217391304348 0 2

"2903" 1.19565217391304 0 2

"2904" 1.22826086956522 0 2

"2905" 1.27173913043478 0 2

"2906" 1.30434782608696 0 2

"2907" 0.0133333333333333 0 2

"2908" 0.0533333333333333 0 2

"2909" 0.106666666666667 4 2

"2910" 0.146666666666667 8 2

"2911" 0.2 7 2

"2912" 0.24 5 2

"2913" 0.293333333333333 8 2

"2914" 0.333333333333333 9 2

"2915" 0.44 10 2

"2916" 0.48 8 2

"2917" 0.52 12 2

"2918" 0.573333333333333 14 2

"2919" 0.613333333333333 17 2

"2920" 0.666666666666667 11 2

"2921" 0.72 15 2

"2922" 0.76 25 2

"2923" 0.813333333333333 15 2

"2924" 0.853333333333333 10 2

"2925" 0.893333333333333 9 2

"2926" 0.946666666666667 0 2

"2927" 1 0 2

"2928" 1.04 0 2

"2929" 1.09333333333333 0 2

"2930" 1.13333333333333 0 2

"2931" 1.18666666666667 0 2

"2932" 1.22666666666667 0 2

"2933" 1.26666666666667 0 2

"2934" 0.0104166666666667 0 2

"2935" 0.0520833333333333 0 2

"2936" 0.0833333333333333 0 2

"2937" 0.125 0 2

"2938" 0.15625 0 2

"2939" 0.197916666666667 1 2

"2940" 0.229166666666667 4 2

"2941" 0.270833333333333 4 2

"2942" 0.302083333333333 6 2

"2943" 0.34375 3 2

"2944" 0.375 4 2

"2945" 0.416666666666667 0 2

"2946" 0.447916666666667 2 2

"2947" 0.489583333333333 4 2

"2948" 0.520833333333333 12 2

"2949" 0.5625 12 2

"2950" 0.59375 10 2

"2951" 0.635416666666667 18 2

"2952" 0.666666666666667 18 2

"2953" 0.75 10 2

"2954" 0.78125 14 2

"2955" 0.8125 19 2

"2956" 0.854166666666667 23 2

"2957" 0.885416666666667 21 2

"2958" 0.927083333333333 5 2

"2959" 0.96875 2 2

"2960" 1 0 2

"2961" 1.04166666666667 0 2

"2962" 1.07291666666667 0 2

"2963" 1.10416666666667 0 2

"2964" 1.14583333333333 0 2

"2965" 1.1875 0 2

"2966" 1.21875 0 2

"2967" 1.26041666666667 0 2

"2968" 1.29166666666667 0 2

"2969" 0.0053475935828877 0 2

"2970" 0.0267379679144385 1 2

"2971" 0.0427807486631016 0 2

"2972" 0.0641711229946524 0 2

"2973" 0.0802139037433155 0 2

"2974" 0.101604278074866 0 2

"2975" 0.117647058823529 0 2

"2976" 0.13903743315508 0 2

"2977" 0.155080213903743 0 2

"2978" 0.176470588235294 0 2

"2979" 0.192513368983957 4 2

"2980" 0.213903743315508 7 2

"2981" 0.229946524064171 6 2

"2982" 0.251336898395722 8 2

"2983" 0.267379679144385 11 2

"2984" 0.288770053475936 10 2

"2985" 0.304812834224599 9 2

"2986" 0.32620320855615 11 2

"2987" 0.342245989304813 14 2

"2988" 0.363636363636364 14 2

"2989" 0.379679144385027 14 2

"2990" 0.422459893048128 20 2

"2991" 0.438502673796791 24 2

"2992" 0.454545454545455 24 2

"2993" 0.475935828877005 10 2

"2994" 0.491978609625668 15 2

"2995" 0.513368983957219 19 2

"2996" 0.53475935828877 16 2

"2997" 0.550802139037433 23 2

"2998" 0.572192513368984 17 2

"2999" 0.588235294117647 26 2

"3000" 0.60427807486631 38 2

"3001" 0.625668449197861 16 2

"3002" 0.647058823529412 24 2

"3003" 0.663101604278075 20 2

"3004" 0.663101604278075 25 2

"3005" 0.684491978609626 21 2

"3006" 0.700534759358289 16 2

"3007" 0.72192513368984 23 2

"3008" 0.737967914438503 30 2

"3009" 0.754010695187166 35 2

"3010" 0.775401069518717 28 2

"3011" 0.79144385026738 30 2

"3012" 0.818181818181818 42 2

"3013" 0.839572192513369 44 2

"3014" 0.850267379679144 31 2

"3015" 0.866310160427808 25 2

"3016" 0.887700534759358 28 2

"3017" 0.903743315508021 34 2

"3018" 0.925133689839572 40 2

"3019" 0.941176470588235 29 2

"3020" 0.967914438502674 5 2

"3021" 0.983957219251337 4 2

"3022" 1 2 2

"3023" 1.02139037433155 0 2

"3024" 0.0175438596491228 0 2

"3025" 0.087719298245614 0 2

"3026" 0.140350877192982 0 2

"3027" 0.210526315789474 0 2

"3028" 0.263157894736842 0 2

"3029" 0.333333333333333 0 2

"3030" 0.385964912280702 1 2

"3031" 0.456140350877193 2 2

"3032" 0.508771929824561 4 2

"3033" 0.578947368421053 5 2

"3034" 0.631578947368421 5 2

"3035" 0.701754385964912 4 2

"3036" 0.754385964912281 7 2

"3037" 0.824561403508772 4 2

"3038" 0.87719298245614 0 2

"3039" 0.947368421052632 0 2

"3040" 1 0 2

"3041" 1.14035087719298 0 2

"3042" 1.19298245614035 0 2

"3043" 1.24561403508772 0 2

"3044" 1.31578947368421 0 2

"3045" 0.00471698113207547 0 2

"3046" 0.0235849056603774 0 2

"3047" 0.0377358490566038 0 2

"3048" 0.0566037735849057 2 2

"3049" 0.0707547169811321 3 2

"3050" 0.089622641509434 5 2

"3051" 0.10377358490566 2 2

"3052" 0.122641509433962 8 2

"3053" 0.136792452830189 7 2

"3054" 0.155660377358491 6 2

"3055" 0.169811320754717 5 2

"3056" 0.188679245283019 4 2

"3057" 0.202830188679245 6 2

"3058" 0.221698113207547 7 2

"3059" 0.235849056603774 7 2

"3060" 0.273584905660377 8 2

"3061" 0.287735849056604 16 2

"3062" 0.30188679245283 10 2

"3063" 0.320754716981132 13 2

"3064" 0.334905660377358 16 2

"3065" 0.35377358490566 12 2

"3066" 0.372641509433962 13 2

"3067" 0.386792452830189 14 2

"3068" 0.405660377358491 10 2

"3069" 0.419811320754717 13 2

"3070" 0.433962264150943 9 2

"3071" 0.452830188679245 7 2

"3072" 0.471698113207547 11 2

"3073" 0.485849056603774 17 2

"3074" 0.504716981132076 21 2

"3075" 0.518867924528302 7 2

"3076" 0.537735849056604 18 2

"3077" 0.55188679245283 14 2

"3078" 0.566037735849057 8 2

"3079" 0.584905660377358 15 2

"3080" 0.599056603773585 10 2

"3081" 0.622641509433962 12 2

"3082" 0.641509433962264 23 2

"3083" 0.650943396226415 17 2

"3084" 0.665094339622642 20 2

"3085" 0.683962264150943 25 2

"3086" 0.69811320754717 9 2

"3087" 0.716981132075472 12 2

"3088" 0.731132075471698 8 2

"3089" 0.754716981132076 6 2

"3090" 0.768867924528302 10 2

"3091" 0.783018867924528 14 2

"3092" 0.80188679245283 12 2

"3093" 1 7 2

"3094" 0.00578034682080925 0 2

"3095" 0.023121387283237 0 2

"3096" 0.046242774566474 2 2

"3097" 0.0635838150289017 4 2

"3098" 0.109826589595376 4 2

"3099" 0.127167630057803 5 2

"3100" 0.144508670520231 6 2

"3101" 0.167630057803468 5 2

"3102" 0.184971098265896 8 2

"3103" 0.208092485549133 12 2

"3104" 0.23121387283237 11 2

"3105" 0.248554913294798 8 2

"3106" 0.271676300578035 17 2

"3107" 0.289017341040462 15 2

"3108" 0.30635838150289 17 2

"3109" 0.329479768786127 12 2

"3110" 0.352601156069364 14 2

"3111" 0.369942196531792 13 2

"3112" 0.393063583815029 21 2

"3113" 0.410404624277457 15 2

"3114" 0.433526011560694 18 2

"3115" 0.450867052023121 27 2

"3116" 0.468208092485549 24 2

"3117" 0.491329479768786 25 2

"3118" 0.508670520231214 24 2

"3119" 0.53757225433526 12 2

"3120" 0.560693641618497 24 2

"3121" 0.572254335260116 12 2

"3122" 0.589595375722543 10 2

"3123" 0.61271676300578 14 2

"3124" 0.630057803468208 13 2

"3125" 0.653179190751445 20 2

"3126" 0.670520231213873 32 2

"3127" 0.872832369942196 21 2

"3128" 0.716763005780347 24 2

"3129" 0.734104046242775 18 2

"3130" 0.757225433526012 14 2

"3131" 0.919075144508671 12 2

"3132" 1 0 2

"3133" 0.0153846153846154 0 2

"3134" 0.0769230769230769 0 2

"3135" 0.123076923076923 1 2

"3136" 0.184615384615385 3 2

"3137" 0.230769230769231 7 2

"3138" 0.292307692307692 7 2

"3139" 0.338461538461538 5 2

"3140" 0.4 2 2

"3141" 0.446153846153846 9 2

"3142" 0.569230769230769 9 2

"3143" 0.615384615384615 8 2

"3144" 0.661538461538462 11 2

"3145" 0.723076923076923 5 2

"3146" 0.769230769230769 10 2

"3147" 0.830769230769231 10 2

"3148" 0.892307692307692 10 2

"3149" 0.938461538461538 17 2

"3150" 1 0 2

"3151" 0.01 0 2

"3152" 0.05 0 2

"3153" 0.09 4 2

"3154" 0.12 8 2

"3155" 0.16 0 2

"3156" 0.19 2 2

"3157" 0.22 5 2

"3158" 0.26 8 2

"3159" 0.29 6 2

"3160" 0.34 10 2

"3161" 0.38 2 2

"3162" 0.4 15 2

"3163" 0.43 13 2

"3164" 0.47 27 2

"3165" 0.5 18 2

"3166" 0.54 25 2

"3167" 0.57 16 2

"3168" 0.62 18 2

"3169" 0.65 25 2

"3170" 0.68 22 2

"3171" 0.72 28 2

"3172" 0.76 12 2

"3173" 0.84 14 2

"3174" 0.9 10 2

"3175" 1 0 2

"3176" 0.00847457627118644 0 2

"3177" 0.0508474576271186 0 2

"3178" 0.076271186440678 10 2

"3179" 0.110169491525424 12 2

"3180" 0.135593220338983 7 2

"3181" 0.169491525423729 7 2

"3182" 0.194915254237288 10 2

"3183" 0.220338983050847 6 2

"3184" 0.254237288135593 8 2

"3185" 0.279661016949153 10 2

"3186" 0.322033898305085 15 2

"3187" 0.355932203389831 18 2

"3188" 0.372881355932203 11 2

"3189" 0.398305084745763 13 2

"3190" 0.432203389830508 11 2

"3191" 0.457627118644068 16 2

"3192" 0.491525423728814 15 2

"3193" 0.516949152542373 12 2

"3194" 0.559322033898305 8 2

"3195" 0.584745762711864 13 2

"3196" 0.610169491525424 14 2

"3197" 0.644067796610169 15 2

"3198" 0.88135593220339 17 2

"3199" 1 12 2

"3200" 0.00869565217391304 0 2

"3201" 0.0260869565217391 0 2

"3202" 0.0521739130434783 0 2

"3203" 0.0869565217391304 1 2

"3204" 0.11304347826087 5 2

"3205" 0.147826086956522 8 2

"3206" 0.173913043478261 8 2

"3207" 0.2 6 2

"3208" 0.234782608695652 8 2

"3209" 0.260869565217391 7 2

"3210" 0.304347826086957 8 2

"3211" 0.339130434782609 20 2

"3212" 0.356521739130435 15 2

"3213" 0.382608695652174 11 2

"3214" 0.417391304347826 18 2

"3215" 0.443478260869565 17 2

"3216" 0.478260869565217 15 2

"3217" 0.504347826086956 12 2

"3218" 0.547826086956522 19 2

"3219" 0.573913043478261 17 2

"3220" 0.6 26 2

"3221" 0.634782608695652 25 2

"3222" 0.878260869565217 12 2

"3223" 1 10 2

**3)Colony level trade-off:**

-counts

sexuals workers japBra

[1,] 16 116 1

[2,] 4 22 2

[3,] 34 112 1

[4,] 95 245 2

[5,] 132 173 1

[6,] 7 22 2

[7,] 119 251 1

[8,] 100 261 2

[9,] 63 228 2

[10,] 57 174 2

[11,] 5 47 1

[12,] 107 118 2

[13,] 14 95 1

[14,] 14 48 2

[15,] 17 104 2

[16,] 17 121 1

[17,] 100 220 2

[18,] 11 65 2

[19,] 40 121 1

[20,] 24 23 2

[21,] 9 134 1

[22,] NA 1 2

[23,] 13 101 1

[24,] 1 12 2

[25,] 51 200 1

[26,] 28 78 2

[27,] 51 209 1

[28,] 32 83 2

[29,] 280 180 1

[30,] 1 7 2

[31,] 9 140 1

[32,] 17 76 1

[33,] NA 1 2

[34,] 3 53 1

[35,] 3 21 2

[36,] 1 40 1

[37,] NA 1 2

[38,] 3 47 1

[39,] 3 43 2

[40,] 1 37 1

[41,] 3 10 2

[42,] 50 126 1

[43,] 5 58 2

[44,] 8 89 1

[45,] 4 58 1

[46,] 4 79 1

[47,] 9 53 1

[48,] 17 166 1

[49,] 9 94 1

[50,] 11 144 1

[51,] 70 294 1

[52,] 7 67 1

[53,] 10 77 1

[54,] 9 22 2

[55,] 10 104 1

[56,] 17 163 1

[57,] 9 91 1

[58,] 8 232 1

[59,] 11 42 1

[60,] 3 115 1

[61,] 18 101 1

[62,] 3 56 1

[63,] 20 140 1

[64,] 88 164 1

[65,] 44 170 2

[66,] 4 69 1

[67,] 12 114 1

[68,] 10 88 1

[69,] 7 76 1

[70,] 4 53 1

[71,] 18 68 1

[72,] 14 79 2

[73,] 9 65 1

[74,] 12 48 1

[75,] 1 16 1

[76,] 2 20 1

[77,] 47 173 1

[78,] 14 113 1

[79,] 35 168 2

[80,] 4 71 1

[81,] 11 26 1

[82,] 3 17 1

[83,] 10 47 1

[84,] 1 6 1

[85,] 9 87 1

[86,] 4 24 2

[87,] 23 114 1

[88,] 71 93 2

-biomass fig. 4

sexbio workbio japbra

[1,] 1.01349 5.13015 1

[2,] 0.16560 0.96537 2

[3,] 2.33380 4.95171 1

[4,] 4.99080 11.21668 2

[5,] 9.22715 7.67292 1

[6,] 0.26550 0.96537 2

[7,] 7.96046 11.15250 1

[8,] 5.38290 11.95220 2

[9,] 3.36570 10.43519 2

[10,] 2.99760 7.95281 2

[11,] 0.23328 2.05206 1

[12,] 5.91960 5.37849 2

[13,] 0.80439 4.19334 1

[14,] 0.64920 2.16059 2

[15,] 0.77430 4.73491 2

[16,] 1.11804 5.35320 1

[17,] 5.45310 10.06743 2

[18,] 0.55470 2.94208 2

[19,] 2.65973 5.35320 1

[20,] 0.95910 1.01134 2

[21,] 0.45447 5.93313 1

[22,] NA NA NA

[23,] 0.69984 4.46100 1

[24,] 2.94682 8.87739 1

[25,] 1.41660 3.53969 2

[26,] 3.16256 9.27888 1

[27,] 1.69050 3.76954 2

[28,] 20.22149 7.98519 1

[29,] 0.43574 6.20079 1

[30,] 1.02558 3.34575 1

[31,] NA NA NA

[32,] 0.14746 2.31972 1

[33,] 0.13380 0.91940 2

[34,] NA NA NA

[35,] 0.11664 2.05206 1

[36,] 0.11400 1.93074 2

[37,] 0.11400 0.41373 2

[38,] 3.48949 5.57625 1

[39,] 0.18210 2.62029 2

[40,] 0.48529 3.92568 1

[41,] 0.19037 2.54277 1

[42,] 0.22119 3.47958 1

[43,] 0.49738 2.31972 1

[44,] 1.05640 7.36065 1

[45,] 0.48529 4.14873 1

[46,] 0.67566 6.37923 1

[47,] 4.40933 13.07073 1

[48,] 0.38074 2.94426 1

[49,] 0.63275 3.39036 1

[50,] 0.44070 0.96537 2

[51,] 0.52663 4.59483 1

[52,] 1.02558 7.22682 1

[53,] 0.43574 4.01490 1

[54,] 0.42365 10.30491 1

[55,] 0.67566 1.82901 1

[56,] 0.08582 5.08554 1

[57,] 1.13013 4.46100 1

[58,] 0.11664 2.45355 1

[59,] 1.20912 6.20079 1

[60,] 6.13713 7.27143 1

[61,] 2.38440 7.76893 2

[62,] 0.15955 3.03348 1

[63,] 0.65693 5.04093 1

[64,] 0.44783 3.88107 1

[65,] 0.34992 3.34575 1

[66,] 0.19037 2.31972 1

[67,] 1.06849 2.98887 1

[68,] 0.67980 3.58566 2

[69,] 0.49738 2.85504 1

[70,] 0.80420 2.09667 1

[71,] NA NA NA

[72,] 0.07373 0.84759 1

[73,] 3.26830 7.67292 1

[74,] 0.89685 4.99632 1

[75,] 1.90650 7.67699 2

[76,] 0.22119 3.12270 1

[77,] 0.73730 1.11525 1

[78,] 0.14746 0.71376 1

[79,] 0.50947 2.05206 1

[80,] NA NA NA

[81,] 0.52820 3.83646 1

[82,] 0.12510 1.05731 2

[83,] 1.59124 5.04093 1

[84,] 3.92880 4.22924 2

-Fig. 5 & 6

"workers" "productivity" "japBra"

"1" 20 0 1

"2" 20 0.04461 1

"3" 20 0 1

"4" 20 0 1

"5" 20 0.22305 1

"6" 20 0.35688 1

"7" 20 0.31227 1

"8" 14 0.84759 1

"9" 17 0.22305 1

"10" 12 0.98142 1

"11" 12 0.84759 1

"12" 16 0.75837 1

"13" 19 1.24908 1

"14" 24 1.38291 1

"15" 22 1.24908 1

"16" 26 0.66915 1

"17" 25 1.38291 1

"18" 19 1.02077 1

"19" 18 1.48576 1

"20" 18 0.78749 1

"21" 20 1.02603 1

"22" 20 1.0979 1

"23" 19 0.8905 1

"24" 18 0.80298 1

"25" 15 0.772 1

"26" 20 0.62454 1

"27" 18 0.93681 1

"28" 20 1.07064 1

"29" 16 0.84759 1

"30" 20 0.49071 1

"31" 25 0.13383 1

"32" 24 0.04461 1

"33" 16 0.04461 1

"34" 20 0.04461 1

"35" 20 0 1

"36" 20 0.04461 1

"37" 20 0.22305 1

"38" 20 0.22305 1

"39" 20 0.17844 1

"40" 20 0.22305 1

"41" 20 0.17844 1

"42" 20 0.13383 1

"43" 20 0.22305 1

"44" 20 0.35688 1

"45" 20 0.40149 1

"46" 20 0.49071 1

"47" 15 0.66915 1

"48" 17 0.4461 1

"49" 17 0.84759 1

"50" 16 0.75837 1

"51" 13 0.4461 1

"52" 10 0.84759 1

"53" 11 1.02603 1

"54" 10 0.84759 1

"55" 10 1.12888 1

"56" 10 0.97972 1

"57" 12 1.53037 1

"58" 13 1.41203 1

"59" 13 1.07064 1

"60" 17 1.39654 1

"61" 18 0.75837 1

"62" 18 0.80298 1

"63" 17 1.17349 1

"64" 18 1.05515 1

"65" 22 1.24908 1

"66" 20 0.96423 1

"67" 22 1.02603 1

"68" 32 0.71376 1

"69" 38 1.23359 1

"70" 38 1.29199 1

"71" 34 0.57993 1

"72" 28 0.8922 1

"73" 33 0.81661 1

"74" 37 0.49071 1

"75" 27 0.56444 1

"76" 24 0.35518 1

"77" 27 0.75837 1

"78" 28 0.69827 1

"79" 26 0.53532 1

"80" 27 0.6518 1

"81" 29 0.69827 1

"82" 31 0.7703 1

"83" 26 0.40149 1

"84" 24 0.54895 1

"85" 25 0.3259 1

"86" 18 0.25047 1

"87" 16 0.25217 1

"88" 15 0.22305 1

"89" 12 0.17674 1

"90" 15 0.25217 1

"91" 15 0.17844 1

"92" 12 0.29678 1

"93" 12 0.29678 1

"94" 20 0 1

"95" 20 0 1

"96" 20 0.08922 1

"97" 20 0.35688 1

"98" 20 0.35688 1

"99" 20 0.4461 1

"100" 20 0.35688 1

"101" 20 0.08922 1

"102" 20 0.31227 1

"103" 20 0.26766 1

"104" 20 0.49071 1

"105" 20 0.62454 1

"106" 20 0.40149 1

"107" 14 0.26766 1

"108" 12 0.4461 1

"109" 11 0.49071 1

"110" 11 0.49071 1

"111" 10 0.62454 1

"112" 10 0.53532 1

"113" 11 0.40149 1

"114" 11 0.62454 1

"115" 9 0.62454 1

"116" 8 0.62454 1

"117" 9 0.31227 1

"118" 9 0.40149 1

"119" 10 0.35688 1

"120" 11 0.49071 1

"121" 14 0.4461 1

"122" 11 0.31227 1

"123" 10 0.17844 1

"124" 6 0 1

"125" 8 0.04461 1

"126" 4 0 1

"127" 2 0 1

"128" 1 0 1

"129" 20 0 1

"130" 20 0 1

"131" 20 0.17844 1

"132" 20 0 1

"133" 20 0 1

"134" 20 0.26766 1

"135" 20 0.26766 1

"136" 20 0.8922 1

"137" 14 1.24908 1

"138" 18 0.93681 1

"139" 20 1.07064 1

"140" 24 1.02603 1

"141" 27 0.53532 1

"142" 29 0.80298 1

"143" 28 0.80298 1

"144" 29 1.57498 1

"145" 32 0.99505 1

"146" 34 0.8321 1

"147" 35 0.8304 1

"148" 37 0.29678 1

"149" 30 0.16295 1

"150" 28 0.26766 1

"151" 27 0.17844 1

"152" 23 0.13383 1

"153" 22 0 1

"154" 20 0 1

"155" 20 0 1

"156" 20 0 1

"157" 20 0.08922 1

"158" 20 0.26766 1

"159" 20 0.4461 1

"160" 20 0.35688 1

"161" 20 0.22305 1

"162" 20 0.26766 1

"163" 20 0.26766 1

"164" 20 0.71376 1

"165" 20 1.07064 1

"166" 20 0.8922 1

"167" 22 1.24908 1

"168" 22 1.07064 1

"169" 23 1.15986 1

"170" 21 0.93681 1

"171" 23 1.24908 1

"172" 28 1.02603 1

"173" 29 1.84078 1

"174" 34 1.24908 1

"175" 30 1.42582 1

"176" 34 1.3383 1

"177" 37 1.32281 1

"178" 29 1.32281 1

"179" 38 1.47213 1

"180" 39 1.67783 1

"181" 42 1.51674 1

"182" 47 1.47213 1

"183" 40 2.19596 1

"184" 58 1.60596 1

"185" 60 1.39654 1

"186" 65 1.81182 1

"187" 55 1.60596 1

"188" 57 1.82715 1

"189" 61 1.73979 1

"190" 57 1.08427 1

"191" 55 1.35193 1

"192" 64 0.92132 1

"193" 68 1.12888 1

"194" 67 1.18898 1

"195" 65 0.47522 1

"196" 52 0.4461 1

"197" 55 0.35688 1

"198" 57 0.78393 1

"199" 54 0.57807 1

"200" 50 0.27959 1

"201" 45 0.66543 1

"202" 46 0.94502 1

"203" 37 0.62268 1

"204" 40 0.45973 1

"205" 38 0.67736 1

"206" 40 0.44424 1

"207" 39 0.47522 1

"208" 32 0.51643 1

"209" 33 0.25217 1

"210" 32 0.33953 1

"211" 31 0.07373 1

"212" 28 0.04461 1

"213" 26 0.13383 1

"214" 26 0.35688 1

"215" 21 0.49071 1

"216" 19 0.4461 1

"217" 19 0.26766 1

"218" 22 0.08922 1

"219" 16 0.13383 1

"220" 14 0 1

"221" 9 0 1

"222" 10 0 1

"223" 20 0 1

"224" 20 0.13383 1

"225" 20 0 1

"226" 20 0 1

"227" 20 0 1

"228" 20 0 1

"229" 20 0 1

"230" 20 0 1

"231" 20 0.35688 1

"232" 7 0.40149 1

"233" 10 0.66915 1

"234" 9 0.53532 1

"235" 11 0.62454 1

"236" 11 0.53532 1

"237" 12 0.57993 1

"238" 13 0.49071 1

"239" 10 0.74118 1

"240" 12 0.57993 1

"241" 12 0.66915 1

"242" 14 0.74288 1

"243" 14 0.66915 1

"244" 14 0.66915 1

"245" 12 0.66915 1

"246" 14 0.97972 1

"247" 17 0.66915 1

"248" 15 0.74288 1

"249" 18 0.93495 1

"250" 18 0.66915 1

"251" 14 0.87501 1

"252" 15 1.02433 1

"253" 15 0.75837 1

"254" 17 0.66915 1

"255" 21 0.57823 1

"256" 25 0.48885 1

"257" 24 0.39979 1

"258" 25 0.08922 1

"259" 24 0.13383 1

"260" 24 0.17844 1

"261" 20 0.13383 1

"262" 20 0.11834 1

"263" 22 0 1

"264" 25 0 1

"265" 22 0 1

"266" 21 0 1

"267" 18 0 1

"268" 20 0 1

"269" 20 0 1

"270" 20 0.22305 1

"271" 20 0 1

"272" 20 0 1

"273" 20 0.26766 1

"274" 20 0.26766 1

"275" 20 0.4461 1

"276" 20 0.84759 1

"277" 19 1.29369 1

"278" 19 1.20447 1

"279" 16 1.11525 1

"280" 21 1.65057 1

"281" 22 2.05206 1

"282" 24 1.96284 1

"283" 26 1.65057 1

"284" 34 1.3383 1

"285" 35 1.3366 1

"286" 41 1.11525 1

"287" 43 0.73932 1

"288" 52 0.53532 1

"289" 55 0.97956 1

"290" 58 0.53532 1

"291" 55 0.66729 1

"292" 64 0.386 1

"293" 20 0 1

"294" 20 0.26766 1

"295" 20 0 1

"296" 20 0 1

"297" 20 0 1

"298" 20 0 1

"299" 20 0.66915 1

"300" 20 1.24908 1

"301" 20 1.51674 1

"302" 20 1.42752 1

"303" 19 1.20261 1

"304" 29 0.90583 1

"305" 33 1.02603 1

"306" 33 1.20447 1

"307" 40 1.24568 1

"308" 49 0.80298 1

"309" 43 0.78749 1

"310" 48 0.40149 1

"311" 40 0.17844 1

"312" 41 0 1

"313" 41 0 1

"314" 20 0 1

"315" 20 0.04461 1

"316" 20 0.08922 1

"317" 20 0.4461 1

"318" 20 0.4461 1

"319" 20 0.35688 1

"320" 20 0.31227 1

"321" 20 0.57993 1

"322" 20 0.53532 1

"323" 20 0.57993 1

"324" 20 1.02603 1

"325" 20 0.75837 1

"326" 18 1.15986 1

"327" 21 1.11525 1

"328" 21 1.42752 1

"329" 23 1.12888 1

"330" 21 1.38291 1

"331" 22 0.98142 1

"332" 22 1.52851 1

"333" 25 1.3383 1

"334" 31 1.3383 1

"335" 25 1.56135 1

"336" 25 1.17163 1

"337" 33 0.65366 1

"338" 32 1.02603 1

"339" 39 1.39654 1

"340" 39 1.42752 1

"341" 38 1.11525 1

"342" 42 1.38291 1

"343" 45 1.20277 1

"344" 44 0.87671 1

"345" 45 0.80298 1

"346" 48 0.68278 1

"347" 40 0.31227 1

"348" 40 0.37051 1

"349" 33 0.40149 1

"350" 28 0.31227 1

"351" 29 0 1

"352" 26 0 1

"353" 25 0 1

"354" 20 0.22305 1

"355" 20 0.25217 1

"356" 20 0.26766 1

"357" 18 0.31227 1

"358" 14 0.17844 1

"359" 13 0.04461 1

"360" 12 0.04461 1

"361" 13 0 1

"362" 20 0 1

"363" 20 0 1

"364" 20 0.04461 1

"365" 20 0.26766 1

"366" 20 0.26766 1

"367" 20 0.35688 1

"368" 20 0.35688 1

"369" 20 0.35688 1

"370" 20 0.22305 1

"371" 20 0.26766 1

"372" 20 0.31227 1

"373" 20 0.4461 1

"374" 20 0.62454 1

"375" 20 0.84759 1

"376" 20 0.8922 1

"377" 13 0.93681 1

"378" 18 1.07064 1

"379" 18 1.20447 1

"380" 22 1.15986 1

"381" 13 0.66915 1

"382" 13 0.74288 1

"383" 16 0.80128 1

"384" 18 0.97972 1

"385" 19 0.84759 1

"386" 21 1.36556 1

"387" 23 0.93681 1

"388" 28 1.02603 1

"389" 26 0.75837 1

"390" 31 0.92132 1

"391" 30 0.57823 1

"392" 20 1.26085 1

"393" 26 0.51983 1

"394" 31 0.69827 1

"395" 32 0.75481 1

"396" 35 0.59356 1

"397" 40 0.49071 1

"398" 38 0.49071 1

"399" 38 0.22305 1

"400" 37 0.35688 1

"401" 34 0.22305 1

"402" 33 0 1

"403" 33 0.14746 1

"404" 33 0.22119 1

"405" 32 0.29492 1

"406" 35 0.14746 1

"407" 20 0 1

"408" 20 0 1

"409" 20 0 1

"410" 20 0 1

"411" 20 0.08922 1

"412" 20 0.31227 1

"413" 20 0.31227 1

"414" 20 0.35688 1

"415" 20 0.22305 1

"416" 20 0.17844 1

"417" 20 0.26766 1

"418" 20 0.53532 1

"419" 20 0.62454 1

"420" 20 0.80298 1

"421" 13 0.62454 1

"422" 16 0.71376 1

"423" 15 0.71376 1

"424" 16 1.07064 1

"425" 16 0.75837 1

"426" 19 0.80298 1

"427" 17 0.80298 1

"428" 15 0.75837 1

"429" 14 0.47522 1

"430" 18 0.75837 1

"431" 21 1.07064 1

"432" 19 0.98142 1

"433" 18 1.02603 1

"434" 19 1.29369 1

"435" 15 1.15816 1

"436" 14 0.93681 1

"437" 14 1.12888 1

"438" 14 0.93681 1

"439" 16 1.02603 1

"440" 21 1.24908 1

"441" 18 1.11525 1

"442" 23 0.8905 1

"443" 25 0.71376 1

"444" 22 1.09976 1

"445" 28 1.02263 1

"446" 27 1.48576 1

"447" 26 1.47213 1

"448" 28 1.4839 1

"449" 28 0.96593 1

"450" 26 1.26085 1

"451" 33 0.78749 1

"452" 33 0.8922 1

"453" 39 1.23359 1

"454" 42 0.8922 1

"455" 41 0.66915 1

"456" 42 0.65366 1

"457" 44 0.71376 1

"458" 44 0.17844 1

"459" 35 0.47522 1

"460" 35 0.8922 1

"461" 35 1.18712 1

"462" 31 1.02603 1

"463" 29 1.24908 1

"464" 30 1.08427 1

"465" 33 1.14437 1

"466" 32 1.43945 1

"467" 31 1.4839 1

"468" 32 0.98142 1

"469" 34 1.47027 1

"470" 34 0.75481 1

"471" 37 1.01054 1

"472" 35 1.36572 1

"473" 40 1.3383 1

"474" 32 1.20261 1

"475" 30 0.81661 1

"476" 46 0.85936 1

"477" 35 0.87485 1

"478" 39 0.68092 1

"479" 40 1.00868 1

"480" 36 1.21268 1

"481" 36 0.78749 1

"482" 37 0.54895 1

"483" 34 0.75481 1

"484" 35 1.00682 1

"485" 40 0.93309 1

"486" 19 0.30871 1

"487" 22 0.48885 1

"488" 19 0.96221 1

"489" 17 0.60549 1

"490" 14 0.15955 1

"491" 11 0.22119 1

"492" 9 0.16125 1

"493" 9 0.11834 1

"494" 7 0 1

"495" 6 0.07373 1

"496" 5 0 1

"497" 20 0 1

"498" 20 0.17844 1

"499" 20 0 1

"500" 20 0 1

"501" 20 0 1

"502" 20 0 1

"503" 20 0.22305 1

"504" 20 0.31227 1

"505" 20 0.35688 1

"506" 20 0.26766 1

"507" 12 0.35688 1

"508" 14 0.40149 1

"509" 14 0.4461 1

"510" 13 0.42891 1

"511" 14 0.35688 1

"512" 16 0.40149 1

"513" 16 0.49071 1

"514" 16 0.386 1

"515" 13 0.22305 1

"516" 9 0.22135 1

"517" 14 0.31227 1

"518" 15 0.22305 1

"519" 15 0.31227 1

"520" 11 0.17844 1

"521" 10 0.40149 1

"522" 10 0.31227 1

"523" 9 0.13383 1

"524" 8 0.13383 1

"525" 10 0.04461 1

"526" 9 0 1

"527" 20 0 1

"528" 20 0.22305 1

"529" 20 0 1

"530" 20 0 1

"531" 20 0 1

"532" 20 0.49071 1

"533" 20 1.02603 1

"534" 20 0.8922 1

"535" 14 0.98142 1

"536" 13 0.98142 1

"537" 14 0.93681 1

"538" 14 1.56135 1

"539" 13 1.51674 1

"540" 15 1.60596 1

"541" 16 1.02603 1

"542" 25 0.84759 1

"543" 25 0.84759 1

"544" 22 1.09976 1

"545" 20 1.15816 1

"546" 21 1.65057 1

"547" 21 1.38291 1

"548" 27 1.73979 1

"549" 33 1.69518 1

"550" 35 0.98142 1

"551" 37 1.24908 1

"552" 38 0.8922 1

"553" 38 0.80128 1

"554" 40 0.57823 1

"555" 42 0.72399 1

"556" 40 0.57993 1

"557" 43 0.60905 1

"558" 39 0.7119 1

"559" 37 0.29678 1

"560" 42 0.17844 1

"561" 20 0 1

"562" 20 0.13383 1

"563" 20 0 1

"564" 20 0 1

"565" 20 0 1

"566" 20 0.26766 1

"567" 20 0.49071 1

"568" 20 1.07064 1

"569" 10 0.75837 1

"570" 14 1.20447 1

"571" 19 1.11525 1

"572" 26 1.42752 1

"573" 24 1.24908 1

"574" 26 1.56135 1

"575" 21 1.47213 1

"576" 23 1.96284 1

"577" 32 1.55965 1

"578" 34 1.49939 1

"579" 42 1.00884 1

"580" 40 1.35193 1

"581" 37 0.96593 1

"582" 38 1.94549 1

"583" 45 0.8922 1

"584" 50 1.03966 1

"585" 50 0.17844 1

"586" 20 0 1

"587" 20 0 1

"588" 20 0.31227 1

"589" 20 0.35688 1

"590" 20 0.35688 1

"591" 20 0.35688 1

"592" 20 0.31227 1

"593" 20 0.40149 1

"594" 20 0.35688 1

"595" 20 0.57993 1

"596" 20 0.80298 1

"597" 20 1.11525 1

"598" 15 1.15986 1

"599" 16 1.11525 1

"600" 16 1.11525 1

"601" 17 1.11355 1

"602" 20 1.05515 1

"603" 23 0.71376 1

"604" 28 0.66915 1

"605" 26 0.75837 1

"606" 23 0.71376 1

"607" 24 0.96593 1

"608" 25 1.2782 1

"609" 20 0.84759 1

"610" 22 1.06724 1

"611" 17 0.80298 1

"612" 16 0.60905 1

"613" 17 0.71376 1

"614" 20 0.57993 1

"615" 20 0.66915 1

"616" 20 0.49071 1

"617" 18 0.49071 1

"618" 17 0.35688 1

"619" 12 0.4461 1

"620" 12 0.31227 1

"621" 11 0.35688 1

"622" 8 0.13383 1

"623" 8 0.04461 1

"624" 10 0.13383 1

"625" 8 0.22305 1

"626" 7 0.17844 1

"627" 8 0.35688 1

"628" 7 0.04461 1

"629" 7 0 1

"630" 6 0 1

"631" 4 0 1

"632" 3 0 1

"633" 1 0.22305 1

"634" 1 0.11834 1

"635" 1 0.17844 1

"636" 1 0.26766 1

"637" 1 0.13383 1

"638" 1 0.08922 1

"639" 1 0.13383 1

"640" 1 0.13383 1

"641" 1 0.26766 1

"642" 1 0.13383 1

"643" 1 0.17844 1

"644" 1 0.08922 1

"645" 1 0.31227 1

"646" 1 0.31227 1

"647" 2 0.22305 1

"648" 2 0.31227 1

"649" 1 0.4461 1

"650" 1 0.35688 1

"651" 3 0.35688 1

"652" 4 0.62454 1

"653" 5 0.53532 1

"654" 8 0.62454 1

"655" 9 0.62454 1

"656" 8 0.62454 1

"657" 10 1.15986 1

"658" 8 0.75837 1

"659" 8 0.57993 1

"660" 8 0.4461 1

"661" 7 0.40149 1

"662" 7 0.49071 1

"663" 9 0.75837 1

"664" 8 0.57993 1

"665" 11 0.75837 1

"666" 11 0.4461 1

"667" 13 0.80298 1

"668" 11 0.75837 1

"669" 6 0.31227 1

"670" 5 0.49071 1

"671" 8 0.04461 1

"672" 9 0.49071 1

"673" 10 0.04461 1

"674" 6 0.22305 1

"675" 7 0.31227 1

"676" 7 0.17844 1

"677" 6 0.26766 1

"678" 8 0.13383 1

"679" 6 0.04461 1

"680" 20 0 1

"681" 20 0.04461 1

"682" 20 0 1

"683" 20 0 1

"684" 20 0 1

"685" 20 0.40149 1

"686" 20 0.35688 1

"687" 20 0.8922 1

"688" 10 0.71376 1

"689" 12 0.62454 1

"690" 15 0.8922 1

"691" 17 0.75837 1

"692" 18 0.8922 1

"693" 18 0.98142 1

"694" 15 0.93681 1

"695" 13 1.07064 1

"696" 23 1.06894 1

"697" 23 1.15986 1

"698" 24 0.87671 1

"699" 25 0.69827 1

"700" 27 0.43061 1

"701" 26 0.49071 1

"702" 26 0.53532 1

"703" 27 0.75837 1

"704" 27 0.75837 1

"705" 26 0.57993 1

"706" 25 0.35688 1

"707" 29 0.57823 1

"708" 28 0.57993 1

"709" 23 0.8321 1

"710" 19 0.57993 1

"711" 21 0.71376 1

"712" 19 0.66915 1

"713" 17 0.35518 1

"714" 17 0.60565 1

"715" 20 0.34139 1

"716" 11 0.4461 1

"717" 11 0 1

"718" 20 0.31057 1

"719" 13 0.26766 1

"720" 11 0.08922 1

"721" 16 0.04461 1

"722" 16 0 1

"723" 13 0 1

"724" 20 0 1

"725" 20 0.04461 1

"726" 20 0 1

"727" 20 0 1

"728" 20 0 1

"729" 20 0 1

"730" 20 0 1

"731" 20 0.17844 1

"732" 20 0.4461 1

"733" 17 0.40149 1

"734" 13 0.40149 1

"735" 16 0.35688 1

"736" 17 0.57993 1

"737" 18 0.35688 1

"738" 18 0.53532 1

"739" 11 0.53532 1

"740" 12 0.53532 1

"741" 7 0.69657 1

"742" 8 0.80298 1

"743" 9 0.92132 1

"744" 14 0.84759 1

"745" 15 0.66745 1

"746" 15 0.93681 1

"747" 15 0.75837 1

"748" 15 0.80298 1

"749" 9 0.80298 1

"750" 12 0.62454 1

"751" 9 0.66745 1

"752" 14 0.78749 1

"753" 14 0.66915 1

"754" 13 0.74288 1

"755" 11 0.40149 1

"756" 15 0.71376 1

"757" 15 0.66915 1

"758" 12 0.49071 1

"759" 11 0.62454 1

"760" 7 1.05515 1

"761" 6 0.62114 1

"762" 5 0.53532 1

"763" 10 0.51983 1

"764" 11 0.35688 1

"765" 10 0.31227 1

"766" 6 0.17844 1

"767" 4 0.26766 1

"768" 6 0.66915 1

"769" 7 0.62454 1

"770" 8 0.57993 1

"771" 6 0.17844 1

"772" 4 0.04461 1

"773" 3 0.66915 1

"774" 5 0.80298 1

"775" 3 0.8922 1

"776" 5 0.84759 1

"777" 7 0.80298 1

"778" 8 0.53532 1

"779" 6 0.92132 1

"780" 5 0.57993 1

"781" 5 0.8922 1

"782" 6 1.09976 1

"783" 8 1.11355 1

"784" 12 0.71376 1

"785" 12 0.62454 1

"786" 11 0.62454 1

"787" 12 0.53532 1

"788" 9 0.62284 1

"789" 11 1.24738 1

"790" 13 1.38121 1

"791" 15 1.36742 1

"792" 15 1.09976 1

"793" 15 1.48406 1

"794" 15 1.46873 1

"795" 18 1.38291 1

"796" 15 1.35023 1

"797" 18 1.47213 1

"798" 23 1.11525 1

"799" 24 1.38291 1

"800" 27 1.3349 1

"801" 26 1.47213 1

"802" 26 1.56135 1

"803" 25 2.28874 1

"804" 39 1.91823 1

"805" 30 1.61789 1

"806" 41 1.51334 1

"807" 36 1.20447 1

"808" 41 1.00714 1

"809" 40 0.84759 1

"810" 47 0.87331 1

"811" 45 1.00884 1

"812" 46 0.92132 1

"813" 47 0.93681 1

"814" 39 1.02417 1

"815" 44 0.47352 1

"816" 35 0.4461 1

"817" 37 0.31227 1

"818" 40 0.13383 1

"819" 37 0 1

"820" 35 0.08922 1

"821" 22 0 1

"822" 20 0 1

"823" 20 0 1

"824" 20 0.08922 1

"825" 20 0.08922 1

"826" 20 0.17844 1

"827" 20 0.26766 1

"828" 20 0.22305 1

"829" 20 0.26766 1

"830" 20 0.35688 1

"831" 20 0.26766 1

"832" 20 0.35688 1

"833" 20 0.71376 1

"834" 20 0.75837 1

"835" 17 0.71376 1

"836" 13 1.11525 1

"837" 15 1.60596 1

"838" 14 0.84759 1

"839" 18 0.57993 1

"840" 20 0.53532 1

"841" 19 1.20447 1

"842" 20 1.65057 1

"843" 23 1.65057 1

"844" 27 1.15986 1

"845" 20 1.51504 1

"846" 26 0.98142 1

"847" 30 1.57498 1

"848" 29 1.2181 1

"849" 29 1.07064 1

"850" 27 0.84589 1

"851" 26 0.93681 1

"852" 20 1.15986 1

"853" 16 1.18898 1

"854" 15 1.02433 1

"855" 8 1.07064 1

"856" 12 0.53532 1

"857" 19 0.71376 1

"858" 14 0.98142 1

"859" 16 0.93681 1

"860" 15 0.53532 1

"861" 14 0.53532 1

"862" 20 0.08922 1

"863" 13 0.31227 1

"864" 8 0.17844 1

"865" 8 0.17844 1

"866" 6 0.26766 1

"867" 6 0.4461 1

"868" 7 0.17844 1

"869" 7 0.35688 1

"870" 7 0.35688 1

"871" 6 0.35688 1

"872" 4 0.13383 1

"873" 4 0.13383 1

"874" 5 0.35688 1

"875" 4 0.35688 1

"876" 4 0.57993 1

"877" 4 0.4461 1

"878" 2 0.17844 1

"879" 5 0.40149 1

"880" 3 0.35688 1

"881" 5 0.49071 1

"882" 6 0.62454 1

"883" 6 0.66915 1

"884" 6 0.80298 1

"885" 6 0.93681 1

"886" 5 0.84759 1

"887" 7 1.07064 1

"888" 5 0.93511 1

"889" 7 1.2181 1

"890" 7 1.07064 1

"891" 13 1.76551 1

"892" 11 1.73979 1

"893" 12 1.60426 1

"894" 15 1.7844 1

"895" 18 1.36402 1

"896" 20 1.67799 1

"897" 26 1.213 1

"898" 25 1.97461 1

"899" 30 1.30206 1

"900" 33 1.61959 1

"901" 35 1.42752 1

"902" 34 1.14437 1

"903" 29 1.20447 1

"904" 29 0.96593 1

"905" 32 1.35023 1

"906" 28 0.97972 1

"907" 33 1.05515 1

"908" 34 1.2181 1

"909" 35 0.25217 1

"910" 38 0.11834 1

"911" 20 0 1

"912" 20 0 1

"913" 20 0 1

"914" 20 0.08922 1

"915" 20 0.22305 1

"916" 20 0.40149 1

"917" 20 0 1

"918" 20 0 1

"919" 20 0.31227 1

"920" 20 0.35688 1

"921" 20 0.75837 1

"922" 20 0.84759 1

"923" 20 1.02603 1

"924" 20 0.75837 1

"925" 20 1.3383 1

"926" 16 1.11525 1

"927" 17 1.7844 1

"928" 16 1.39654 1

"929" 23 1.47213 1

"930" 25 1.18898 1

"931" 23 1.84264 1

"932" 24 1.38121 1

"933" 25 1.69146 1

"934" 26 1.14251 1

"935" 27 1.85813 1

"936" 33 1.9901 1

"937" 34 1.3383 1

"938" 30 1.82901 1

"939" 35 2.7621 1

"940" 36 2.4345 1

"941" 39 2.25776 1

"942" 41 2.41545 1

"943" 46 1.6642 1

"944" 45 2.34528 1

"945" 49 2.62657 1

"946" 47 1.11525 1

"947" 50 1.8699 1

"948" 55 2.68465 1

"949" 55 2.30067 1

"950" 56 1.78068 1

"951" 59 3.08056 1

"952" 62 1.25713 1

"953" 66 2.34775 1

"954" 64 0.63631 1

"955" 66 1.30004 1

"956" 63 1.49195 1

"957" 59 0.97584 1

"958" 57 0.98947 1

"959" 51 0.41512 1

"960" 49 0.51797 1

"961" 48 0.60719 1

"962" 49 0.54709 1

"963" 42 0.17844 1

"964" 39 0.39979 1

"965" 38 0.41342 1

"966" 38 0.27959 1

"967" 38 0.62268 1

"968" 38 0.56274 1

"969" 34 0.35688 1

"970" 33 0.50434 1

"971" 29 0.60549 1

"972" 30 0.56258 1

"973" 24 0.3259 1

"974" 20 0.25047 1

"975" 19 0.57807 1

"976" 18 0.57807 1

"977" 17 0.68278 1

"978" 17 1.00868 1

"979" 12 1.05329 1

"980" 10 0.34139 1

"981" 10 0.43061 1

"982" 12 0.44424 1

"983" 7 0.50434 1

"984" 9 0.53532 1

"985" 20 0 1

"986" 20 0.04461 1

"987" 20 0.22305 1

"988" 20 0.22305 1

"989" 20 0.35688 1

"990" 20 0.26766 1

"991" 20 0.53532 1

"992" 20 0.62454 1

"993" 20 0.71376 1

"994" 20 0.57993 1

"995" 27 0.8922 1

"996" 20 0.71376 1

"997" 22 0.93681 1

"998" 25 1.15986 1

"999" 28 0.80298 1

"1000" 28 0.62454 1

"1001" 28 0.71376 1

"1002" 28 1.02603 1

"1003" 30 1.02263 1

"1004" 30 0.84759 1

"1005" 22 0.66915 1

"1006" 22 1.11525 1

"1007" 25 0.57993 1

"1008" 20 0.87671 1

"1009" 18 1.38121 1

"1010" 16 1.59047 1

"1011" 22 0.98142 1

"1012" 20 1.29369 1

"1013" 24 1.38291 1

"1014" 22 1.56135 1

"1015" 21 1.38291 1

"1016" 22 1.56135 1

"1017" 25 1.38121 1

"1018" 22 0.66915 1

"1019" 27 0.8922 1

"1020" 24 0.98142 1

"1021" 20 0.75667 1

"1022" 17 0.04461 1

"1023" 22 0.29678 1

"1024" 20 0 1

"1025" 20 0 1

"1026" 20 0 1

"1027" 20 0.26766 1

"1028" 20 0 1

"1029" 20 0.22305 1

"1030" 20 0.35688 1

"1031" 20 0.4461 1

"1032" 20 0.62454 1

"1033" 17 0.69641 1

"1034" 20 0.49071 1

"1035" 20 0.60905 1

"1036" 16 0.49071 1

"1037" 15 0.68278 1

"1038" 14 0.56444 1

"1039" 18 0.49071 1

"1040" 18 0.80298 1

"1041" 19 0.84759 1

"1042" 13 0.71376 1

"1043" 11 0.78749 1

"1044" 12 0.97802 1

"1045" 13 0.98142 1

"1046" 17 0.66915 1

"1047" 15 0.93681 1

"1048" 16 0.62284 1

"1049" 12 0.8905 1

"1050" 11 0.74288 1

"1051" 14 0.57993 1

"1052" 14 0.74288 1

"1053" 11 0.75667 1

"1054" 10 0.31227 1

"1055" 12 0.62454 1

"1056" 12 0.4461 1

"1057" 8 0.04461 1

"1058" 5 0 1

"1059" 2 0 1

"1060" 1 0 1

"1061" 20 0 1

"1062" 20 0.13383 1

"1063" 20 0 1

"1064" 20 0 1

"1065" 20 0 1

"1066" 20 0.31227 1

"1067" 20 0.40149 1

"1068" 20 0.80298 1

"1069" 11 0.71376 1

"1070" 17 0.66915 1

"1071" 20 0.75837 1

"1072" 24 0.71376 1

"1073" 24 0.71376 1

"1074" 27 0.66915 1

"1075" 35 0.34139 1

"1076" 35 0.17844 1

"1077" 39 0.08922 1

"1078" 25 0.04461 1

"1079" 25 0.11834 1

"1080" 21 0.04461 1

"1081" 19 0.04461 1

"1082" 20 0 1

"1083" 20 0 1

"1084" 20 0.08922 1

"1085" 20 0.13383 1

"1086" 20 0.26766 1

"1087" 20 0.40149 1

"1088" 20 0.26766 1

"1089" 20 0.08922 1

"1090" 20 0.35688 1

"1091" 20 0.13383 1

"1092" 20 0.75837 1

"1093" 20 0.49071 1

"1094" 20 0.93681 1

"1095" 13 0.8922 1

"1096" 12 0.80298 1

"1097" 15 0.62454 1

"1098" 18 1.07064 1

"1099" 17 0.75837 1

"1100" 17 0.22305 1

"1101" 1 0.26766 1

"1102" 9 0.4461 1

"1103" 9 0.62454 1

"1104" 8 0.40149 1

"1105" 7 0.22305 1

"1106" 7 0.26766 1

"1107" 8 0.26766 1

"1108" 7 0.04461 1

"1109" 6 0.08922 1

"1110" 2 0.22305 1

"1111" 1 0 1

"1112" 20 0 1

"1113" 20 0 1

"1114" 20 0.26766 1

"1115" 20 0.40149 1

"1116" 20 0.4461 1

"1117" 20 0.40149 1

"1118" 20 0.31227 1

"1119" 20 0.22305 1

"1120" 20 0.17844 1

"1121" 20 0.80298 1

"1122" 20 0.80298 1

"1123" 20 0.98142 1

"1124" 20 0.93681 1

"1125" 19 0.98142 1

"1126" 22 1.24908 1

"1127" 23 0.93681 1

"1128" 18 0.53532 1

"1129" 13 0.57993 1

"1130" 13 0.4461 1

"1131" 14 0.26766 1

"1132" 13 0.53532 1

"1133" 11 0.386 1

"1134" 11 0.35688 1

"1135" 10 0.4461 1

"1136" 6 0.57993 1

"1137" 5 0.62454 1

"1138" 5 0.53362 1

"1139" 5 0.40149 1

"1140" 8 0.40149 1

"1141" 8 0.49071 1

"1142" 7 0.17844 1

"1143" 5 0.04461 1

"1144" 4 0.04461 1

"1145" 20 0 1

"1146" 20 0.22305 1

"1147" 20 0 1

"1148" 20 0.31227 1

"1149" 20 0.26766 1

"1150" 20 0.35688 1

"1151" 20 0.26766 1

"1152" 20 0.57993 1

"1153" 20 0.75837 1

"1154" 20 0.75837 1

"1155" 20 0.26766 1

"1156" 21 0.71376 1

"1157" 24 1.15986 1

"1158" 26 2.2305 1

"1159" 25 1.24908 1

"1160" 28 2.27511 1

"1161" 28 2.04866 1

"1162" 33 1.96284 1

"1163" 41 1.76535 1

"1164" 40 0.98142 1

"1165" 44 1.0378 1

"1166" 46 1.49939 1

"1167" 47 1.30732 1

"1168" 48 1.77898 1

"1169" 50 1.12888 1

"1170" 46 1.14251 1

"1171" 51 0.5316 1

"1172" 50 0.36865 1

"1173" 40 0.29492 1

"1174" 45 0.22119 1

"1175" 20 0 1

"1176" 20 0 1

"1177" 20 0.08922 1

"1178" 20 0.22305 1

"1179" 20 0.22305 1

"1180" 20 0.31227 1

"1181" 20 0.4461 1

"1182" 20 0.35688 1

"1183" 20 0.35688 1

"1184" 20 0.71376 1

"1185" 20 0.84759 1

"1186" 22 0.8922 1

"1187" 20 0.84759 1

"1188" 19 0.84759 1

"1189" 17 0.71376 1

"1190" 18 0.62454 1

"1191" 16 0.57993 1

"1192" 25 1.26271 1

"1193" 19 0.84759 1

"1194" 17 1.15986 1

"1195" 18 0.8321 1

"1196" 18 1.51488 1

"1197" 7 1.20447 1

"1198" 10 0.8922 1

"1199" 12 0.80298 1

"1200" 13 0.66915 1

"1201" 10 0.31227 1

"1202" 5 0.13383 1

"1203" 4 0.22135 1

"1204" 5 0.22305 1

"1205" 4 0.35688 1

"1206" 5 0.4461 1

"1207" 5 0.53532 1

"1208" 5 0.4461 1

"1209" 10 0.17844 1

"1210" 13 0.17844 1

"1211" 15 0.04461 1

"1212" 20 0 1

"1213" 20 0 1

"1214" 20 0 1

"1215" 20 0 1

"1216" 20 0.35688 1

"1217" 20 0.4461 1

"1218" 20 0.80298 1

"1219" 15 0.80298 1

"1220" 13 0.71376 1

"1221" 13 0.98142 1

"1222" 16 0.98142 1

"1223" 17 0.80298 1

"1224" 22 0.66915 1

"1225" 22 0.4461 1

"1226" 24 0.65196 1

"1227" 27 0.35688 1

"1228" 21 0.40149 1

"1229" 22 0.26766 1

"1230" 21 0.08922 1

"1231" 21 0.08922 1

"1232" 19 0.17844 1

"1233" 17 0.49071 1

"1234" 14 0.25217 1

"1235" 11 0 1

"1236" 8 0.35688 1

"1237" 7 0.31227 1

"1238" 7 0.22305 1

"1239" 6 0 1

"1240" 5 0.04461 1

"1241" 5 0.08922 1

"1242" 4 0.49071 1

"1243" 2 0 1

"1244" 20 0 1

"1245" 20 0 1

"1246" 20 0.08922 1

"1247" 20 0 1

"1248" 20 0 1

"1249" 20 0.53532 1

"1250" 20 0.53532 1

"1251" 20 0.71376 1

"1252" 20 1.07064 1

"1253" 11 0.62454 1

"1254" 13 1.11525 1

"1255" 13 1.02603 1

"1256" 16 0.98142 1

"1257" 19 1.02603 1

"1258" 19 0.84759 1

"1259" 20 0.71376 1

"1260" 24 0.92132 1

"1261" 30 0.62454 1

"1262" 35 0.47522 1

"1263" 35 0.22305 1

"1264" 40 0.13383 1

"1265" 35 0.20756 1

"1266" 49 0.08922 1

"1267" 20 0 1

"1268" 20 0 1

"1269" 20 0 1

"1270" 20 0.08922 1

"1271" 20 0.31227 1

"1272" 20 0.31227 1

"1273" 20 0.22305 1

"1274" 20 0.4461 1

"1275" 20 0.13383 1

"1276" 20 0.53532 1

"1277" 20 0.4461 1

"1278" 20 0.80298 1

"1279" 20 0.71376 1

"1280" 15 0.84759 1

"1281" 17 0.96593 1

"1282" 16 0.69827 1

"1283" 17 0.74288 1

"1284" 17 0.53532 1

"1285" 12 0.66745 1

"1286" 11 1.05345 1

"1287" 13 0.66915 1

"1288" 16 0.75837 1

"1289" 10 0.49071 1

"1290" 11 0.62284 1

"1291" 10 0.47522 1

"1292" 7 0.17844 1

"1293" 5 0.04461 1

"1294" 5 0 1

"1295" 3 0 1

"1296" 1 0 1

"1297" 20 0 1

"1298" 20 0.08922 1

"1299" 20 0 1

"1300" 20 0 1

"1301" 20 0 1

"1302" 20 0.4461 1

"1303" 20 1.02603 1

"1304" 20 1.38291 1

"1305" 20 1.29369 1

"1306" 17 1.56135 1

"1307" 18 1.15986 1

"1308" 19 1.56135 1

"1309" 24 1.47213 1

"1310" 22 1.30562 1

"1311" 30 1.33644 1

"1312" 31 2.25622 1

"1313" 36 1.76891 1

"1314" 35 2.63199 1

"1315" 48 2.15491 1

"1316" 35 1.29369 1

"1317" 39 1.11525 1

"1318" 35 1.60596 1

"1319" 46 1.6642 1

"1320" 35 1.55965 1

"1321" 32 1.02603 1

"1322" 40 1.15986 1

"1323" 46 0.71376 1

"1324" 45 0.56444 1

"1325" 20 0 1

"1326" 20 0.04461 1

"1327" 20 0 1

"1328" 20 0 1

"1329" 20 0 1

"1330" 20 0.31227 1

"1331" 20 0.71376 1

"1332" 20 0.71376 1

"1333" 20 0.84759 1

"1334" 10 1.07064 1

"1335" 12 1.15986 1

"1336" 15 1.07064 1

"1337" 17 1.3383 1

"1338" 19 1.11525 1

"1339" 27 1.02603 1

"1340" 27 1.09806 1

"1341" 36 1.41203 1

"1342" 35 0.92132 1

"1343" 34 1.14267 1

"1344" 40 0.78749 1

"1345" 42 0.93681 1

"1346" 40 0.66915 1

"1347" 59 0.57993 1

"1348" 45 0.43061 1

"1349" 47 0.22305 1

"1350" 20 0 1

"1351" 20 0.04461 1

"1352" 20 0 1

"1353" 20 0 1

"1354" 20 0 1

"1355" 20 0.4461 1

"1356" 20 0.93681 1

"1357" 20 0.8922 1

"1358" 14 0.8922 1

"1359" 14 0.66915 1

"1360" 9 1.24908 1

"1361" 11 1.60596 1

"1362" 12 1.02603 1

"1363" 17 1.05515 1

"1364" 25 1.38105 1

"1365" 30 1.07064 1

"1366" 21 1.3383 1

"1367" 19 1.51674 1

"1368" 21 1.15986 1

"1369" 19 1.29029 1

"1370" 20 1.20447 1

"1371" 24 0.84759 1

"1372" 28 1.2782 1

"1373" 31 1.07064 1

"1374" 24 1.3383 1

"1375" 19 0.98142 1

"1376" 21 1.02603 1

"1377" 16 0.71376 1

"1378" 15 1.02603 1

"1379" 14 0.93681 1

"1380" 18 1.38291 1

"1381" 19 0.98142 1

"1382" 20 0.84759 1

"1383" 23 0.57993 1

"1384" 22 0.57993 1

"1385" 17 0.4461 1

"1386" 12 0.57993 1

"1387" 12 0.74288 1

"1388" 14 0.86122 1

"1389" 11 0.40149 1

"1390" 10 0.17844 1

"1391" 12 0.22305 1

"1392" 10 0.71376 1

"1393" 4 0.49071 1

"1394" 4 0.40149 1

"1395" 2 0.53532 1

"1396" 5 0.57993 1

"1397" 6 0.17844 1

"1398" 6 0.04461 1

"1399" 3 0.26766 1

"1400" 3 0.35688 1

"1401" 3 0.22305 1

"1402" 2 0 1

"1403" 20 0 1

"1404" 20 0.08922 1

"1405" 20 0 1

"1406" 20 0.4461 1

"1407" 20 0.4461 1

"1408" 20 0.35688 1

"1409" 20 0.4461 1

"1410" 20 0.4461 1

"1411" 20 0.84759 1

"1412" 20 0.8922 1

"1413" 20 1.38291 1

"1414" 16 1.60596 1

"1415" 15 1.65057 1

"1416" 20 1.47213 1

"1417" 24 1.60426 1

"1418" 25 1.87362 1

"1419" 31 1.3383 1

"1420" 29 1.79617 1

"1421" 37 2.0485 1

"1422" 35 2.61464 1

"1423" 27 2.57189 1

"1424" 27 2.98887 1

"1425" 29 2.49646 1

"1426" 31 2.05206 1

"1427" 37 2.08118 1

"1428" 42 2.53767 1

"1429" 46 2.31632 1

"1430" 47 2.9407 1

"1431" 43 3.6543 1

"1432" 53 3.79185 1

"1433" 59 3.47788 1

"1434" 67 2.31972 1

"1435" 56 2.23887 1

"1436" 63 2.63029 1

"1437" 78 1.76195 1

"1438" 75 2.27511 1

"1439" 78 2.11883 1

"1440" 60 1.75156 1

"1441" 68 1.51318 1

"1442" 61 1.42752 1

"1443" 62 1.18558 1

"1444" 55 0.62454 1

"1445" 31 0.50434 1

"1446" 36 0.08922 1

"1447" 1 0 1

"1448" 20 0 1

"1449" 20 0 1

"1450" 20 0 1

"1451" 20 0 1

"1452" 20 0 1

"1453" 20 0.26766 1

"1454" 20 0.26766 1

"1455" 20 0.71376 1

"1456" 20 0.62454 1

"1457" 16 0.57993 1

"1458" 15 0.53532 1

"1459" 20 0.71376 1

"1460" 21 0.75667 1

"1461" 18 0.49071 1

"1462" 23 0.53532 1

"1463" 25 0.3259 1

"1464" 30 0.40149 1

"1465" 30 0.40149 1

"1466" 26 0.47522 1

"1467" 22 0.13383 1

"1468" 25 0.35688 1

"1469" 24 0.31227 1

"1470" 21 0.60905 1

"1471" 19 0.08922 1

"1472" 16 0.4461 1

"1473" 19 0.40149 1

"1474" 17 0.66915 1

"1475" 16 0.53532 1

"1476" 17 0.4461 1

"1477" 18 0.35688 1

"1478" 11 0.31227 1

"1479" 10 0.4444 1

"1480" 11 0.53532 1

"1481" 14 0.08922 1

"1482" 15 0 1

"1483" 20 0 1

"1484" 20 0 1

"1485" 20 0.04461 1

"1486" 20 0.08922 1

"1487" 20 0.22305 1

"1488" 20 0.40149 1

"1489" 20 0.35688 1

"1490" 20 0.35688 1

"1491" 20 0.31227 1

"1492" 20 0.40149 1

"1493" 20 0.53532 1

"1494" 20 0.8922 1

"1495" 20 0.84759 1

"1496" 11 0.66915 1

"1497" 12 0.80298 1

"1498" 16 1.20447 1

"1499" 15 1.15986 1

"1500" 8 0.97972 1

"1501" 10 0.93511 1

"1502" 13 1.29183 1

"1503" 16 0.98142 1

"1504" 16 1.07064 1

"1505" 16 0.75497 1

"1506" 15 0.98142 1

"1507" 18 0.8922 1

"1508" 18 1.02603 1

"1509" 20 1.07064 1

"1510" 20 1.02603 1

"1511" 19 0.53532 1

"1512" 20 0.84759 1

"1513" 12 1.15986 1

"1514" 10 1.07064 1

"1515" 9 1.20447 1

"1516" 8 1.11525 1

"1517" 12 0.93681 1

"1518" 18 1.38291 1

"1519" 15 1.37951 1

"1520" 16 1.20447 1

"1521" 18 0.35688 1

"1522" 8 0.26766 1

"1523" 8 0 1

"1524" 20 0 1

"1525" 20 0.17844 1

"1526" 20 0 1

"1527" 20 0 1

"1528" 20 0.53532 1

"1529" 20 0.53532 1

"1530" 20 1.07064 1

"1531" 20 1.20447 1

"1532" 10 1.38291 1

"1533" 12 1.11525 1

"1534" 14 1.65057 1

"1535" 14 1.65057 1

"1536" 17 1.87362 1

"1537" 18 1.02433 1

"1538" 22 1.3383 1

"1539" 26 1.65057 1

"1540" 28 2.85504 1

"1541" 28 2.42087 1

"1542" 31 2.45355 1

"1543" 36 1.97647 1

"1544" 38 2.2305 1

"1545" 35 2.18419 1

"1546" 40 2.64562 1

"1547" 31 1.92846 1

"1548" 33 1.82901 1

"1549" 38 1.33644 1

"1550" 45 0.71376 1

"1551" 53 0.98142 1

"1552" 55 1.15986 1

"1553" 48 1.24908 1

"1554" 20 0 1

"1555" 20 0.04461 1

"1556" 20 0 1

"1557" 20 0 1

"1558" 20 0.53532 1

"1559" 20 0.35688 1

"1560" 20 1.11525 1

"1561" 20 1.07064 1

"1562" 17 1.20447 1

"1563" 20 1.11525 1

"1564" 20 1.15986 1

"1565" 21 1.07064 1

"1566" 18 1.24908 1

"1567" 23 1.64717 1

"1568" 25 1.24908 1

"1569" 32 1.2147 1

"1570" 36 0.93681 1

"1571" 43 0.53532 1

"1572" 45 0.3843 1

"1573" 49 0.35688 1

"1574" 40 0.4461 1

"1575" 34 0.22305 1

"1576" 27 0.08922 1

"1577" 27 0 1

"1578" 20 0 1

"1579" 20 0.26766 1

"1580" 20 0 1

"1581" 20 0 1

"1582" 20 0.35688 1

"1583" 20 1.15986 1

"1584" 20 1.24908 1

"1585" 20 1.15986 1

"1586" 20 1.64717 1

"1587" 22 1.23359 1

"1588" 28 1.67969 1

"1589" 34 1.06894 1

"1590" 30 0.99505 1

"1591" 45 0.57993 1

"1592" 33 0.4461 1

"1593" 33 0.31227 1

"1594" 33 0.53532 1

"1595" 33 0.75837 1

"1596" 30 0.84759 1

"1597" 30 0.66915 1

"1598" 35 0.53532 1

"1599" 36 0.35688 1

"1600" 38 0.35688 1

"1601" 38 0.17844 1

"1602" 35 0.26766 1

"1603" 38 0.17844 1

"1604" 28 0 1

"1605" 27 0 1

"1606" 25 0 1

"1607" 25 0 1

"1608" 22 0.04461 1

"1609" 20 0 1

"1610" 20 0 1

"1611" 20 0.08922 1

"1612" 20 0.22305 1

"1613" 20 0.40149 1

"1614" 20 0.31227 1

"1615" 20 0.4461 1

"1616" 20 0.26766 1

"1617" 20 0.22305 1

"1618" 20 0.4461 1

"1619" 20 0.26766 1

"1620" 20 0.40149 1

"1621" 12 0.35688 1

"1622" 11 0.40149 1

"1623" 14 0.75837 1

"1624" 12 0.84759 1

"1625" 13 0.66915 1

"1626" 13 0.75651 1

"1627" 16 0.39979 1

"1628" 20 0.4461 1

"1629" 20 0.22305 1

"1630" 15 0.13383 1

"1631" 15 0.26766 1

"1632" 10 0.31227 1

"1633" 10 0.69827 1

"1634" 10 0.71376 1

"1635" 10 0.98142 1

"1636" 10 0.71376 1

"1637" 14 0.63817 1

"1638" 12 0.22305 1

"1639" 15 0.47522 1

"1640" 11 0.35688 1

"1641" 12 0.386 1

"1642" 12 0.26766 1

"1643" 11 0.08922 1

"1644" 9 0 1

"1645" 7 0.04291 1

"1646" 20 0 1

"1647" 20 0.31227 1

"1648" 20 0 1

"1649" 20 0 1

"1650" 20 0.40149 1

"1651" 20 0.8922 1

"1652" 20 0.93681 1

"1653" 20 0.80298 1

"1654" 10 1.07064 1

"1655" 7 0.98142 1

"1656" 10 1.15986 1

"1657" 11 1.02603 1

"1658" 16 1.20447 1

"1659" 13 0.84759 1

"1660" 23 1.20447 1

"1661" 23 1.11525 1

"1662" 20 0.62454 1

"1663" 23 0.57993 1

"1664" 26 0.98142 1

"1665" 26 0.71376 1

"1666" 29 0.75837 1

"1667" 29 0.71376 1

"1668" 28 0.71376 1

"1669" 29 0.80128 1

"1670" 28 0.66915 1

"1671" 21 0.53532 1

"1672" 16 0.97972 1

"1673" 11 0.80298 1

"1674" 12 0.8922 1

"1675" 10 1.11525 1

"1676" 13 0.80298 1

"1677" 13 0.62454 1

"1678" 19 0.66915 1

"1679" 15 0.75837 1

"1680" 18 0.8922 1

"1681" 15 0.71376 1

"1682" 17 0.4461 1

"1683" 21 0.57993 1

"1684" 24 0.26766 1

"1685" 22 0.22305 1

"1686" 16 0 1

"1687" 14 0 1

"1688" 12 0 1

"1689" 11 0 1

"1690" 20 0 1

"1691" 20 0.17844 1

"1692" 20 0 1

"1693" 20 0 1

"1694" 20 0.4461 1

"1695" 20 0.80298 1

"1696" 20 1.11525 1

"1697" 20 1.15986 1

"1698" 13 1.15986 1

"1699" 16 1.11525 1

"1700" 15 0.80298 1

"1701" 17 0.84759 1

"1702" 17 1.29369 1

"1703" 17 1.65057 1

"1704" 17 1.7243 1

"1705" 20 1.23189 1

"1706" 25 1.47213 1

"1707" 33 1.48576 1

"1708" 35 1.17349 1

"1709" 32 0.87331 1

"1710" 43 1.05515 1

"1711" 41 0.42891 1

"1712" 40 0.60905 1

"1713" 37 0.54895 1

"1714" 35 0.71376 1

"1715" 39 0.75837 1

"1716" 35 0.93681 1

"1717" 37 0.60905 1

"1718" 20 0 1

"1719" 20 0 1

"1720" 20 0 1

"1721" 20 0 1

"1722" 20 0.13383 1

"1723" 20 0 1

"1724" 20 0.49071 1

"1725" 20 0.40149 1

"1726" 15 0.31227 1

"1727" 15 0.4461 1

"1728" 17 0.31227 1

"1729" 17 0.35688 1

"1730" 18 0.42891 1

"1731" 21 0.49071 1

"1732" 17 0.31227 1

"1733" 19 0.22305 1

"1734" 18 0.26766 1

"1735" 19 0.13383 1

"1736" 16 0.49071 1

"1737" 14 0.40149 1

"1738" 12 0.53532 1

"1739" 15 0.4461 1

"1740" 15 0.26766 1

"1741" 14 0.22305 1

"1742" 17 0.22305 1

"1743" 18 0.26766 1

"1744" 20 0.26766 1

"1745" 19 0.40149 1

"1746" 20 0.40149 1

"1747" 20 0.35688 1

"1748" 19 0.22305 1

"1749" 16 0.13383 1

"1750" 16 0.08922 1

"1751" 15 0.13383 1

"1752" 12 0.17844 1

"1753" 11 0.22305 1

"1754" 9 0.17844 1

"1755" 20 0 1

"1756" 20 0 1

"1757" 20 0.04461 1

"1758" 20 0.08922 1

"1759" 20 0.22305 1

"1760" 20 0.22305 1

"1761" 20 0.26766 1

"1762" 20 0.22305 1

"1763" 20 0.49071 1

"1764" 20 0.26766 1

"1765" 20 0.62454 1

"1766" 20 0.98142 1

"1767" 20 0.71376 1

"1768" 8 0.8922 1

"1769" 9 0.71376 1

"1770" 10 1.07064 1

"1771" 11 0.93681 1

"1772" 9 1.07064 1

"1773" 10 1.11525 1

"1774" 12 1.15986 1

"1775" 16 0.80298 1

"1776" 16 1.3366 1

"1777" 19 0.84589 1

"1778" 19 1.67969 1

"1779" 21 1.15986 1

"1780" 24 1.87362 1

"1781" 27 1.3383 1

"1782" 21 1.3315 1

"1783" 15 1.3383 1

"1784" 27 1.2181 1

"1785" 29 1.65057 1

"1786" 30 1.47043 1

"1787" 32 1.57312 1

"1788" 30 1.07064 1

"1789" 26 0.62454 1

"1790" 32 0.8922 1

"1791" 36 0.80298 1

"1792" 27 0.81661 1

"1793" 25 0.43061 1

"1794" 29 0.26766 1

"1795" 39 0.20756 1

"1796" 33 0.04461 1

"1797" 20 0 1

"1798" 20 0.13383 1

"1799" 20 0 1

"1800" 20 0 1

"1801" 20 0 1

"1802" 20 0.4461 1

"1803" 20 0 1

"1804" 20 0.22305 1

"1805" 20 0.35688 1

"1806" 11 0.57993 1

"1807" 10 0.49071 1

"1808" 8 0.62454 1

"1809" 9 0.75837 1

"1810" 10 0.4461 1

"1811" 10 0.31227 1

"1812" 10 0.57993 1

"1813" 11 0.53532 1

"1814" 11 0.62454 1

"1815" 13 0.49071 1

"1816" 13 0.40149 1

"1817" 15 0.57993 1

"1818" 18 0.62454 1

"1819" 15 0.71376 1

"1820" 16 0.75837 1

"1821" 17 0.66915 1

"1822" 18 0 1

"1823" 20 0.53362 1

"1824" 8 0.4461 1

"1825" 4 0.17844 1

"1826" 6 0.35688 1

"1827" 7 0.35518 1

"1828" 7 0.22305 1

"1829" 6 0.17844 1

"1830" 8 0.29678 1

"1831" 6 0.26766 1

"1832" 6 0.17844 1

"1833" 5 0.04461 1

"1834" 5 0 1

"1835" 6 0.08922 1

"1836" 20 0 1

"1837" 20 0.08922 1

"1838" 20 0 1

"1839" 20 0 1

"1840" 20 0.4461 1

"1841" 20 0.75837 1

"1842" 20 0.71376 1

"1843" 20 0.53532 1

"1844" 18 0.57993 1

"1845" 21 0.35688 1

"1846" 17 0.4461 1

"1847" 20 0.26766 1

"1848" 20 0.53532 1

"1849" 17 0.31227 1

"1850" 16 0.4461 1

"1851" 16 0.62454 1

"1852" 18 0.49071 1

"1853" 17 0.49071 1

"1854" 18 0.80128 1

"1855" 17 0.75837 1

"1856" 18 0.62454 1

"1857" 13 0.40149 1

"1858" 16 0.53532 1

"1859" 13 0.57993 1

"1860" 13 0.65366 1

"1861" 14 0.49071 1

"1862" 15 0.35688 1

"1863" 15 0.57993 1

"1864" 13 0.84589 1

"1865" 13 0.80128 1

"1866" 12 0.4461 1

"1867" 12 0.71036 1

"1868" 12 0.386 1

"1869" 14 0.79958 1

"1870" 11 0.57993 1

"1871" 13 0.71376 1

"1872" 12 0.66915 1

"1873" 10 0.84759 1

"1874" 14 0 1

"1875" 20 0 1

"1876" 20 0.08922 1

"1877" 20 0 1

"1878" 20 0.22305 1

"1879" 20 0.22305 1

"1880" 20 0.26766 1

"1881" 20 0.57993 1

"1882" 20 0.84759 1

"1883" 20 0.66915 1

"1884" 18 0.57993 1

"1885" 16 0 1

"1886" 20 0.57993 1

"1887" 7 0.31227 1

"1888" 8 0.35688 1

"1889" 9 0.35688 1

"1890" 9 0.49071 1

"1891" 6 0.66915 1

"1892" 8 0.62454 1

"1893" 8 0.66915 1

"1894" 9 0.53532 1

"1895" 9 0.80298 1

"1896" 11 0.53532 1

"1897" 11 1.02603 1

"1898" 11 0 1

"1899" 20 0.75837 1

"1900" 5 0.57993 1

"1901" 20 0.62454 1

"1902" 7 0.75837 1

"1903" 8 0.8922 1

"1904" 7 0.57823 1

"1905" 7 0.35688 1

"1906" 12 0.54895 1

"1907" 9 0.39979 1

"1908" 10 0.22305 1

"1909" 10 0.51983 1

"1910" 7 0.26766 1

"1911" 8 0.4461 1

"1912" 8 0.26766 1

"1913" 9 0.04461 1

"1914" 9 0.08922 1

"1915" 9 0.13213 1

"1916" 7 0.53532 1

"1917" 8 0.66915 1

"1918" 8 0.53532 1

"1919" 4 0.04461 1

"1920" 4 0.04461 1

"1921" 3 0 1

"1922" 4 0.04461 1

"1923" 3 0.08922 1

"1924" 3 0 1

"1925" 2 0 1

"1926" 20 0 1

"1927" 20 0.13383 1

"1928" 20 0 1

"1929" 20 0 1

"1930" 20 0.35688 1

"1931" 20 0.53532 1

"1932" 20 0.71376 1

"1933" 20 0.40149 1

"1934" 20 0.35688 1

"1935" 19 0.49071 1

"1936" 19 0.4461 1

"1937" 22 0.35688 1

"1938" 22 0.4461 1

"1939" 20 0.31227 1

"1940" 20 0.40149 1

"1941" 19 0.4461 1

"1942" 20 0.35688 1

"1943" 12 0.54895 1

"1944" 13 0.53532 1

"1945" 11 0.35688 1

"1946" 13 0.4461 1

"1947" 14 0.4444 1

"1948" 14 0.49071 1

"1949" 18 0.53532 1

"1950" 17 0.31227 1

"1951" 17 0.31227 1

"1952" 12 0.35688 1

"1953" 11 0.31227 1

"1954" 10 0.26766 1

"1955" 5 0.26766 1

"1956" 6 0.35688 1

"1957" 7 0.17844 1

"1958" 9 0.13383 1

"1959" 5 0.08922 1

"1960" 3 0.04461 1

"1961" 3 0 1

"1962" 20 0 1

"1963" 20 0.08922 1

"1964" 20 0 1

"1965" 20 0.22305 1

"1966" 20 0.22305 1

"1967" 20 0.13383 1

"1968" 20 0.13383 1

"1969" 20 0.13383 1

"1970" 20 0.35688 1

"1971" 20 0.26766 1

"1972" 17 0.40149 1

"1973" 20 0.40149 1

"1974" 18 0.31227 1

"1975" 15 0.40149 1

"1976" 18 0.35688 1

"1977" 13 0.75837 1

"1978" 15 0.49071 1

"1979" 16 0.72739 1

"1980" 15 0.53532 1

"1981" 16 0.53532 1

"1982" 17 0.53532 1

"1983" 17 0.71376 1

"1984" 19 0.80298 1

"1985" 21 0.71376 1

"1986" 20 0.40149 1

"1987" 24 0.4461 1

"1988" 26 0.49071 1

"1989" 27 0.51813 1

"1990" 28 0.31227 1

"1991" 28 0.29678 1

"1992" 29 0.26596 1

"1993" 28 0.49071 1

"1994" 25 0.43061 1

"1995" 25 0.53532 1

"1996" 26 0.13383 1

"1997" 26 0.13383 1

"1998" 26 0.04291 1

"1999" 21 0 1

"2000" 20 0 1

"2001" 20 0.04461 1

"2002" 20 0.08922 1

"2003" 20 0.04461 1

"2004" 20 0 1

"2005" 20 0 1

"2006" 20 0.17844 1

"2007" 20 0.31227 1

"2008" 20 0.13383 1

"2009" 20 0.22305 1

"2010" 20 0.26766 1

"2011" 20 0.35688 1

"2012" 20 0.35688 1

"2013" 20 0.75837 1

"2014" 20 0.75667 1

"2015" 20 0.80298 1

"2016" 11 0.98142 1

"2017" 12 0.96593 1

"2018" 16 0.53532 1

"2019" 15 0.56444 1

"2020" 16 0.56444 1

"2021" 12 0.62454 1

"2022" 9 0.84759 1

"2023" 7 0.78749 1

"2024" 10 0.57993 1

"2025" 11 0.72739 1

"2026" 10 0.29678 1

"2027" 8 0.17844 1

"2028" 10 0.29678 1

"2029" 10 0.34139 1

"2030" 5 0.25217 1

"2031" 5 0.35688 1

"2032" 3 0.13383 1

"2033" 1 0.13383 1

"2034" 2 0.04461 1

"2035" 3 0.04461 1

"2036" 3 0.04461 1

"2037" 3 0 1

"2038" 20 0 1

"2039" 20 0.04461 1

"2040" 20 0 1

"2041" 20 0 1

"2042" 20 0.13383 1

"2043" 20 0.17844 1

"2044" 20 0.35688 1

"2045" 20 0.4461 1

"2046" 20 0.40149 1

"2047" 20 0.49071 1

"2048" 20 0.22305 1

"2049" 20 0.35688 1

"2050" 15 0.22305 1

"2051" 15 0.26766 1

"2052" 15 0.386 1

"2053" 15 0.08922 1

"2054" 16 0.04461 1

"2055" 16 0.04461 1

"2056" 16 0 1

"2057" 15 0.13383 1

"2058" 12 0.08922 1

"2059" 11 0.17844 1

"2060" 9 0.08922 1

"2061" 9 0.17844 1

"2062" 8 0.17844 1

"2063" 7 0.17844 1

"2064" 6 0.04461 1

"2065" 5 0.13383 1

"2066" 4 0.22305 1

"2067" 2 0.31227 1

"2068" 2 0.22305 1

"2069" 1 0.22305 1

"2070" 2 0.08922 1

"2071" 3 0.13383 1

"2072" 3 0 1

"2073" 3 0.08922 1

"2074" 3 0 1

"2075" 1 0 1

"2076" 20 0 1

"2077" 20 0 1

"2078" 20 0.04461 1

"2079" 20 0 1

"2080" 20 0.26766 1

"2081" 20 0.22305 1

"2082" 20 0.35688 1

"2083" 20 0.35688 1

"2084" 20 0.53532 1

"2085" 20 0.8922 1

"2086" 20 0.98142 1

"2087" 20 0.75837 1

"2088" 17 0.98142 1

"2089" 17 0.71376 1

"2090" 18 1.02603 1

"2091" 15 1.69518 1

"2092" 17 2.05206 1

"2093" 14 1.3383 1

"2094" 15 1.56135 1

"2095" 20 1.73979 1

"2096" 22 1.39654 1

"2097" 28 1.11525 1

"2098" 25 1.84264 1

"2099" 22 1.61959 1

"2100" 24 1.38291 1

"2101" 22 1.96284 1

"2102" 25 2.01938 1

"2103" 24 1.54586 1

"2104" 31 1.2181 1

"2105" 39 1.41203 1

"2106" 37 1.2782 1

"2107" 35 1.781 1

"2108" 37 1.38291 1

"2109" 43 1.57312 1

"2110" 50 1.51488 1

"2111" 47 0.87671 1

"2112" 46 1.12702 1

"2113" 32 0.75837 1

"2114" 37 0.63817 1

"2115" 36 0.50264 1

"2116" 35 0.14746 1

"2117" 32 0.51797 1

"2118" 33 0.22119 1

"2119" 34 0.07373 1

"2120" 31 0 1

"2121" 28 0 1

"2122" 29 0 1

"2123" 28 0.07373 1

"2124" 28 0 1

"2125" 27 0 1

"2126" 20 0 1

"2127" 20 0 1

"2128" 20 0.35688 1

"2129" 20 0.4461 1

"2130" 20 0.35688 1

"2131" 20 0.40149 1

"2132" 20 0.13383 1

"2133" 20 0.35688 1

"2134" 20 0.35688 1

"2135" 20 0.31227 1

"2136" 20 0.71376 1

"2137" 17 1.02603 1

"2138" 17 1.15986 1

"2139" 16 0.71376 1

"2140" 20 0.8922 1

"2141" 22 0.75837 1

"2142" 26 0.66915 1

"2143" 20 0.53532 1

"2144" 22 0.62454 1

"2145" 22 0.69827 1

"2146" 17 0.49071 1

"2147" 22 0.53532 1

"2148" 21 0.49071 1

"2149" 23 0.17844 1

"2150" 24 0.23668 1

"2151" 25 0.04461 1

"2152" 24 0.04461 1

"2153" 20 0.04461 1

"2154" 18 0.04461 1

"2155" 18 0.04461 1

"2156" 18 0.04461 1

"2157" 20 0 1

"2158" 20 0 1

"2159" 20 0.13383 1

"2160" 20 0.26766 1

"2161" 20 0 1

"2162" 20 0.13383 1

"2163" 20 0.04461 1

"2164" 20 0.26766 1

"2165" 20 0.53532 1

"2166" 20 0.40149 1

"2167" 23 0.22305 1

"2168" 23 0.40149 1

"2169" 21 0.35688 1

"2170" 18 0.31227 1

"2171" 14 0.37051 1

"2172" 15 0.08922 1

"2173" 9 0.23668 1

"2174" 8 0.16295 1

"2175" 9 0.2658 1

"2176" 9 0.19207 1

"2177" 7 0.04461 1

"2178" 20 0 1

"2179" 20 0 1

"2180" 20 0.26766 1

"2181" 20 0.31227 1

"2182" 20 0.13383 1

"2183" 20 0.13383 1

"2184" 20 0.13383 1

"2185" 20 0.08922 1

"2186" 20 0.17844 1

"2187" 20 0.13383 1

"2188" 20 0.26766 1

"2189" 20 0.49071 1

"2190" 20 0.49071 1

"2191" 5 0.4461 1

"2192" 8 0.22305 1

"2193" 9 0.22305 1

"2194" 10 0.22305 1

"2195" 9 0.13383 1

"2196" 9 0.17844 1

"2197" 8 0.26766 1

"2198" 8 0.29678 1

"2199" 7 0.31227 1

"2200" 7 0.31227 1

"2201" 7 0.47522 1

"2202" 4 0.08922 1

"2203" 5 0.08922 1

"2204" 2 0.04461 1

"2205" 1 0 1

"2206" 20 0 1

"2207" 20 0.04461 1

"2208" 20 0.22305 1

"2209" 20 0.22305 1

"2210" 20 0.17844 1

"2211" 20 0.13383 1

"2212" 20 0.17844 1

"2213" 20 0.26766 1

"2214" 20 0.04461 1

"2215" 20 0.35688 1

"2216" 20 0.35688 1

"2217" 20 0.53532 1

"2218" 20 0.35688 1

"2219" 10 0.40149 1

"2220" 9 0.22305 1

"2221" 11 0.40149 1

"2222" 9 0.4461 1

"2223" 9 0.53532 1

"2224" 5 0.49071 1

"2225" 8 0.31227 1

"2226" 6 0.35688 1

"2227" 7 0.26766 1

"2228" 6 0.26766 1

"2229" 4 0.40149 1

"2230" 5 0.386 1

"2231" 3 0.31227 1

"2232" 8 0.4461 1

"2233" 8 0.31227 1

"2234" 9 0.26766 1

"2235" 9 0.17844 1

"2236" 11 0.22305 1

"2237" 6 0.22135 1

"2238" 5 0.26766 1

"2239" 6 0.53532 1

"2240" 6 0.66915 1

"2241" 5 0.78749 1

"2242" 5 0.40149 1

"2243" 8 0.4461 1

"2244" 9 0.49071 1

"2245" 10 0.49071 1

"2246" 8 0.40149 1

"2247" 8 0.31227 1

"2248" 9 0.40149 1

"2249" 9 0.13383 1

"2250" 6 0.22135 1

"2251" 6 0.13383 1

"2252" 6 0.25047 1

"2253" 5 0.13213 1

"2254" 5 0.13043 1

"2255" 6 0.08922 1

"2256" 6 0.11834 1

"2257" 20 0 1

"2258" 20 0.04461 1

"2259" 20 0.26766 1

"2260" 20 0.35688 1

"2261" 20 0.35688 1

"2262" 20 0.22305 1

"2263" 20 0 1

"2264" 20 0 1

"2265" 20 0.26766 1

"2266" 20 0.35688 1

"2267" 20 0.49071 1

"2268" 20 0.49071 1

"2269" 20 0.26766 1

"2270" 2 0 1

"2271" 2 0 1

"2272" 20 0 2

"2273" 20 0 2

"2274" 20 0 2

"2275" 20 0 2

"2276" 20 0.04597 2

"2277" 20 0.13791 2

"2278" 20 0.22985 2

"2279" 20 0.13791 2

"2280" 20 0.04597 2

"2281" 20 0.13791 2

"2282" 20 0.09194 2

"2283" 20 0.13791 2

"2284" 20 0 2

"2285" 20 0 2

"2286" 20 0 2

"2287" 20 0.13791 2

"2288" 20 0.18388 2

"2289" 20 0.18388 2

"2290" 20 0.22985 2

"2291" 20 0.36776 2

"2292" 20 0.41373 2

"2293" 20 0.50567 2

"2294" 20 0.78149 2

"2295" 10 0.50567 2

"2296" 12 0.55164 2

"2297" 8 0.59761 2

"2298" 9 0.68955 2

"2299" 8 0.31752 2

"2300" 6 0.09194 2

"2301" 5 0.09194 2

"2302" 5 0.47073 2

"2303" 5 0.41373 2

"2304" 4 0.27582 2

"2305" 3 0.27582 2

"2306" 3 0.36776 2

"2307" 3 0.54737 2

"2308" 3 0.59334 2

"2309" 3 0.13791 2

"2310" 4 0.13791 2

"2311" 4 0.18388 2

"2312" 5 0.13791 2

"2313" 6 0.13791 2

"2314" 7 0.09194 2

"2315" 6 0.18388 2

"2316" 6 0.13791 2

"2317" 20 0 2

"2318" 20 0 2

"2319" 20 0 2

"2320" 20 0 2

"2321" 20 0.04597 2

"2322" 20 0.04597 2

"2323" 20 0.04597 2

"2324" 20 0.13791 2

"2325" 20 0.04597 2

"2326" 20 0.09194 2

"2327" 20 0.13791 2

"2328" 20 0.13791 2

"2329" 20 0.13791 2

"2330" 20 0.22985 2

"2331" 20 0.04597 2

"2332" 20 0.18388 2

"2333" 20 0.13791 2

"2334" 20 0 2

"2335" 20 0.22985 2

"2336" 20 0.64358 2

"2337" 20 0.82746 2

"2338" 20 0.73552 2

"2339" 14 0.55164 2

"2340" 18 0.32179 2

"2341" 15 0.86489 2

"2342" 13 0.40519 2

"2343" 14 0.45116 2

"2344" 10 0.61796 2

"2345" 11 0.62223 2

"2346" 8 0.34214 2

"2347" 7 0.0417 2

"2348" 6 0 2

"2349" 6 0 2

"2350" 6 0.0417 2

"2351" 20 0 2

"2352" 20 0 2

"2353" 20 0 2

"2354" 20 0 2

"2355" 20 0 2

"2356" 20 0 2

"2357" 20 0 2

"2358" 20 0.04597 2

"2359" 20 0.09194 2

"2360" 20 0.09194 2

"2361" 20 0.27582 2

"2362" 20 0.27582 2

"2363" 20 0.41373 2

"2364" 20 0.55164 2

"2365" 19 0.73552 2

"2366" 18 0.55164 2

"2367" 18 0.78149 2

"2368" 16 0.78149 2

"2369" 18 0.78149 2

"2370" 22 1.0334 2

"2371" 6 0 2

"2372" 20 0 2

"2373" 20 0 2

"2374" 20 0 2

"2375" 20 0.04597 2

"2376" 20 0.36776 2

"2377" 20 0.18388 2

"2378" 20 0.41373 2

"2379" 20 0.44682 2

"2380" 10 0.42476 2

"2381" 7 0.75758 2

"2382" 7 0.73125 2

"2383" 16 0.64358 2

"2384" 18 0.65461 2

"2385" 20 0 2

"2386" 20 0 2

"2387" 20 0 2

"2388" 20 0.27582 2

"2389" 20 0.13791 2

"2390" 20 0.22985 2

"2391" 20 0.09194 2

"2392" 20 0.18388 2

"2393" 20 0.09194 2

"2394" 20 0.36776 2

"2395" 20 0.64358 2

"2396" 20 0.68955 2

"2397" 20 0.4597 2

"2398" 19 0.27582 2

"2399" 18 0.36776 2

"2400" 20 0.41373 2

"2401" 20 0.4597 2

"2402" 17 0.55164 2

"2403" 17 0.73552 2

"2404" 16 0.64358 2

"2405" 14 1.14925 2

"2406" 18 1.14925 2

"2407" 20 1.65492 2

"2408" 23 1.24119 2

"2409" 25 1.21728 2

"2410" 24 1.8388 2

"2411" 30 1.59607 2

"2412" 30 1.74259 2

"2413" 35 2.07541 2

"2414" 55 2.70796 2

"2415" 70 2.12565 2

"2416" 78 2.50017 2

"2417" 80 1.53907 2

"2418" 83 1.87189 2

"2419" 70 1.63777 2

"2420" 83 1.75177 2

"2421" 75 1.68801 2

"2422" 74 1.76707 2

"2423" 58 1.51516 2

"2424" 60 1.06834 2

"2425" 58 0.56267 2

"2426" 64 0.13791 2

"2427" 20 0 2

"2428" 20 0 2

"2429" 20 0.18388 2

"2430" 20 0.22985 2

"2431" 20 0.13791 2

"2432" 20 0.22985 2

"2433" 20 0.27582 2

"2434" 20 0.36776 2

"2435" 20 0.4597 2

"2436" 20 0.59761 2

"2437" 20 0.68955 2

"2438" 20 0.59761 2

"2439" 14 0.59761 2

"2440" 13 0.50567 2

"2441" 14 0.36776 2

"2442" 13 0.50567 2

"2443" 11 0.41373 2

"2444" 10 0.55164 2

"2445" 7 0.86916 2

"2446" 7 0.36349 2

"2447" 7 0.55164 2

"2448" 7 0.78149 2

"2449" 9 0.80355 2

"2450" 7 0.64358 2

"2451" 7 0.64358 2

"2452" 7 0.71161 2

"2453" 7 0.50567 2

"2454" 7 0.87343 2

"2455" 5 1.05731 2

"2456" 5 1.10328 2

"2457" 9 1.33313 2

"2458" 8 1.14925 2

"2459" 11 1.39013 2

"2460" 14 1.09901 2

"2461" 17 1.47104 2

"2462" 17 1.28716 2

"2463" 18 1.47104 2

"2464" 15 1.47104 2

"2465" 14 0.68955 2

"2466" 17 0.77722 2

"2467" 20 0.78149 2

"2468" 24 0.75758 2

"2469" 21 0.5167 2

"2470" 25 0.47073 2

"2471" 20 0 2

"2472" 20 0 2

"2473" 20 0.04597 2

"2474" 20 0.04597 2

"2475" 20 0 2

"2476" 20 0 2

"2477" 20 0 2

"2478" 20 0 2

"2479" 20 0 2

"2480" 20 0 2

"2481" 20 0 2

"2482" 20 0.04597 2

"2483" 20 0 2

"2484" 20 0 2

"2485" 20 0 2

"2486" 20 0 2

"2487" 20 0 2

"2488" 20 0 2

"2489" 20 0.04597 2

"2490" 20 0.13791 2

"2491" 20 0.22985 2

"2492" 20 0.36776 2

"2493" 20 0.32179 2

"2494" 20 0.4597 2

"2495" 20 0.73552 2

"2496" 20 0.64358 2

"2497" 17 0.82746 2

"2498" 17 0.96537 2

"2499" 18 0.96537 2

"2500" 21 1.14925 2

"2501" 25 1.05731 2

"2502" 24 0.96537 2

"2503" 24 1.51701 2

"2504" 26 1.65492 2

"2505" 29 1.47104 2

"2506" 30 1.47104 2

"2507" 27 2.66626 2

"2508" 31 2.36653 2

"2509" 36 2.76496 2

"2510" 42 2.86793 2

"2511" 44 2.91817 2

"2512" 47 2.51981 2

"2513" 50 2.77599 2

"2514" 49 3.10205 2

"2515" 61 2.73429 2

"2516" 55 3.02114 2

"2517" 60 2.86608 2

"2518" 56 2.72141 2

"2519" 65 1.8805 2

"2520" 83 0.87343 2

"2521" 76 1.45816 2

"2522" 20 0 2

"2523" 20 0 2

"2524" 20 0 2

"2525" 20 0.04597 2

"2526" 20 0 2

"2527" 20 0 2

"2528" 20 0 2

"2529" 20 0 2

"2530" 20 0 2

"2531" 20 0 2

"2532" 20 0 2

"2533" 20 0 2

"2534" 20 0 2

"2535" 20 0 2

"2536" 20 0.18388 2

"2537" 20 0.36776 2

"2538" 20 0.4597 2

"2539" 20 0.27582 2

"2540" 20 0.36776 2

"2541" 20 0.27582 2

"2542" 20 0.59761 2

"2543" 20 0.64358 2

"2544" 20 0.64358 2

"2545" 20 1.01134 2

"2546" 20 0.41373 2

"2547" 2 0.4597 2

"2548" 5 0.30898 2

"2549" 12 0.55164 2

"2550" 13 0.41373 2

"2551" 13 0.41373 2

"2552" 12 0.50567 2

"2553" 14 0.55164 2

"2554" 20 0 2

"2555" 20 0 2

"2556" 20 0 2

"2557" 20 0.04597 2

"2558" 20 0.13791 2

"2559" 20 0.27582 2

"2560" 20 0.22985 2

"2561" 20 0.27582 2

"2562" 20 0.32179 2

"2563" 20 0.73552 2

"2564" 20 0.64358 2

"2565" 19 0.59761 2

"2566" 17 0.73552 2

"2567" 14 0.96537 2

"2568" 13 1.33313 2

"2569" 15 1.24119 2

"2570" 18 1.14925 2

"2571" 23 1.19522 2

"2572" 26 1.65492 2

"2573" 25 1.33313 2

"2574" 24 1.60895 2

"2575" 31 1.60895 2

"2576" 32 1.55871 2

"2577" 35 1.67698 2

"2578" 19 1.18234 2

"2579" 22 1.10328 2

"2580" 35 1.32025 2

"2581" 35 1.24119 2

"2582" 38 1.33313 2

"2583" 38 1.14925 2

"2584" 39 0.87343 2

"2585" 40 1.30922 2

"2586" 40 1.34416 2

"2587" 46 1.61998 2

"2588" 44 1.8388 2

"2589" 48 1.4625 2

"2590" 45 1.19522 2

"2591" 44 1.93074 2

"2592" 47 1.63101 2

"2593" 46 2.59453 2

"2594" 48 2.42353 2

"2595" 47 1.50413 2

"2596" 56 1.29264 2

"2597" 45 1.74686 2

"2598" 47 0.66379 2

"2599" 45 0.57185 2

"2600" 38 0.53449 2

"2601" 36 0.88261 2

"2602" 37 1.05546 2

"2603" 44 0.90652 2

"2604" 20 0 2

"2605" 20 0 2

"2606" 20 0 2

"2607" 20 0.04597 2

"2608" 20 0.32179 2

"2609" 20 0.41373 2

"2610" 20 0.27582 2

"2611" 20 0.27582 2

"2612" 20 0.22985 2

"2613" 20 0.41373 2

"2614" 20 0.55164 2

"2615" 20 0.87343 2

"2616" 20 0.82746 2

"2617" 20 0.82746 2

"2618" 20 1.19522 2

"2619" 21 1.47104 2

"2620" 22 1.14925 2

"2621" 25 1.33313 2

"2622" 25 1.64638 2

"2623" 27 1.60895 2

"2624" 32 1.71192 2

"2625" 34 1.55871 2

"2626" 39 1.94853 2

"2627" 34 1.43183 2

"2628" 34 1.97671 2

"2629" 35 2.12565 2

"2630" 41 1.84983 2

"2631" 51 1.8388 2

"2632" 50 1.32025 2

"2633" 49 1.24119 2

"2634" 40 1.91359 2

"2635" 60 2.16977 2

"2636" 55 2.14771 2

"2637" 68 2.17838 2

"2638" 55 1.59607 2

"2639" 61 1.96383 2

"2640" 57 1.95956 2

"2641" 60 1.28716 2

"2642" 52 2.30768 2

"2643" 55 2.2985 2

"2644" 60 3.30984 2

"2645" 59 2.58535 2

"2646" 65 2.27459 2

"2647" 70 3.53791 2

"2648" 76 3.04505 2

"2649" 73 4.13794 2

"2650" 75 3.39324 2

"2651" 78 2.69935 2

"2652" 83 2.75642 2

"2653" 86 3.06711 2

"2654" 89 3.88852 2

"2655" 93 3.32087 2

"2656" 82 3.20075 2

"2657" 91 2.99908 2

"2658" 93 2.75635 2

"2659" 105 2.93596 2

"2660" 100 2.4125 2

"2661" 20 0 2

"2662" 20 0 2

"2663" 20 0 2

"2664" 20 0.04597 2

"2665" 20 0.18388 2

"2666" 20 0.27582 2

"2667" 20 0.22985 2

"2668" 20 0.27582 2

"2669" 20 0.32179 2

"2670" 20 0.09194 2

"2671" 20 0.41373 2

"2672" 20 0.96537 2

"2673" 20 0.82746 2

"2674" 16 0.78149 2

"2675" 16 0.82746 2

"2676" 14 0.82319 2

"2677" 15 0.55164 2

"2678" 12 0.55164 2

"2679" 14 0.55164 2

"2680" 11 0.54737 2

"2681" 12 0.50567 2

"2682" 15 0.4597 2

"2683" 18 0.42049 2

"2684" 9 0.08767 2

"2685" 7 0.08767 2

"2686" 5 0 2

"2687" 3 0 2

"2688" 20 0 2

"2689" 20 0.04597 2

"2690" 20 0.22985 2

"2691" 20 0.27582 2

"2692" 20 0.22985 2

"2693" 20 0.27582 2

"2694" 20 0.27582 2

"2695" 20 0.32179 2

"2696" 20 0.41373 2

"2697" 20 0.50567 2

"2698" 20 0.36776 2

"2699" 20 0.64358 2

"2700" 20 0.87343 2

"2701" 15 0.87343 2

"2702" 17 1.10328 2

"2703" 18 1.33313 2

"2704" 20 1.19522 2

"2705" 24 0.9194 2

"2706" 27 1.60895 2

"2707" 30 1.74686 2

"2708" 31 1.70089 2

"2709" 40 1.78856 2

"2710" 46 2.16059 2

"2711" 56 1.55871 2

"2712" 63 1.92896 2

"2713" 50 2.66199 2

"2714" 57 2.36226 2

"2715" 56 2.45847 2

"2716" 53 2.43641 2

"2717" 56 2.1502 2

"2718" 61 2.82623 2

"2719" 67 2.71714 2

"2720" 66 2.61659 2

"2721" 72 2.18941 2

"2722" 70 1.98347 2

"2723" 77 3.85408 2

"2724" 78 2.65338 2

"2725" 79 3.11308 2

"2726" 87 3.64757 2

"2727" 95 2.12565 2

"2728" 90 1.93992 2

"2729" 107 1.64638 2

"2730" 103 2.16977 2

"2731" 93 1.94426 2

"2732" 96 1.87004 2

"2733" 102 1.68801 2

"2734" 74 1.07937 2

"2735" 85 0.87343 2

"2736" 87 0.33282 2

"2737" 20 0 2

"2738" 20 0 2

"2739" 20 0.04597 2

"2740" 20 0.18388 2

"2741" 20 0.22985 2

"2742" 20 0.36776 2

"2743" 20 0.22985 2

"2744" 20 0.18388 2

"2745" 20 0.18388 2

"2746" 20 0.27582 2

"2747" 20 0.59761 2

"2748" 20 0.73552 2

"2749" 20 0.55164 2

"2750" 20 0.78149 2

"2751" 16 1.01134 2

"2752" 18 0.78149 2

"2753" 19 1.19522 2

"2754" 19 1.01134 2

"2755" 22 0.96537 2

"2756" 21 1.01134 2

"2757" 22 1.05731 2

"2758" 23 0.82746 2

"2759" 24 1.53907 2

"2760" 28 1.90256 2

"2761" 24 1.65492 2

"2762" 25 1.60895 2

"2763" 32 1.8388 2

"2764" 30 1.96383 2

"2765" 32 1.97671 2

"2766" 30 2.60741 2

"2767" 40 2.26356 2

"2768" 40 2.45235 2

"2769" 43 2.7582 2

"2770" 50 3.12411 2

"2771" 65 2.64235 2

"2772" 72 3.18296 2

"2773" 70 3.1002 2

"2774" 71 2.47811 2

"2775" 76 2.42353 2

"2776" 68 3.03466 2

"2777" 62 2.18265 2

"2778" 78 3.41957 2

"2779" 83 3.36684 2

"2780" 79 3.37787 2

"2781" 75 3.43921 2

"2782" 69 3.58139 2

"2783" 61 3.13948 2

"2784" 63 2.99908 2

"2785" 79 2.85014 2

"2786" 61 2.88323 2

"2787" 1 0 2

"2788" 20 0 2

"2789" 20 0 2

"2790" 20 0.04597 2

"2791" 20 0.32179 2

"2792" 20 0.18388 2

"2793" 20 0.36776 2

"2794" 20 0.41373 2

"2795" 20 0.18388 2

"2796" 20 0.55164 2

"2797" 20 0.55164 2

"2798" 20 0.41373 2

"2799" 13 0.96537 2

"2800" 20 1.05731 2

"2801" 9 1.14925 2

"2802" 9 1.60895 2

"2803" 17 1.33313 2

"2804" 19 1.37056 2

"2805" 21 1.51701 2

"2806" 26 1.56298 2

"2807" 25 2.05577 2

"2808" 34 2.43641 2

"2809" 33 2.32981 2

"2810" 37 2.24826 2

"2811" 47 2.52408 2

"2812" 40 2.49341 2

"2813" 45 2.91817 2

"2814" 48 2.96414 2

"2815" 57 3.55563 2

"2816" 51 2.79563 2

"2817" 59 2.22435 2

"2818" 50 2.50444 2

"2819" 62 2.30099 2

"2820" 71 1.60895 2

"2821" 79 2.18087 2

"2822" 77 1.75789 2

"2823" 71 2.65153 2

"2824" 79 2.39044 2

"2825" 82 3.04505 2

"2826" 80 2.62947 2

"2827" 75 1.28716 2

"2828" 71 0.41373 2

"2829" 20 0 2

"2830" 20 0 2

"2831" 20 0 2

"2832" 20 0.04597 2

"2833" 20 0.32179 2

"2834" 20 0.22985 2

"2835" 20 0.32179 2

"2836" 20 0.27582 2

"2837" 20 0.27582 2

"2838" 20 0.68955 2

"2839" 20 0.73552 2

"2840" 11 0.9194 2

"2841" 12 0.73552 2

"2842" 15 0.96537 2

"2843" 20 1.01134 2

"2844" 22 0.96537 2

"2845" 23 1.33313 2

"2846" 26 1.01134 2

"2847" 28 1.24119 2

"2848" 28 1.69662 2

"2849" 23 1.0181 2

"2850" 28 1.81489 2

"2851" 29 2.08644 2

"2852" 29 1.93074 2

"2853" 27 1.79774 2

"2854" 29 1.90683 2

"2855" 29 1.69904 2

"2856" 33 2.12814 2

"2857" 39 1.81119 2

"2858" 37 1.62916 2

"2859" 38 1.23749 2

"2860" 41 2.08886 2

"2861" 36 1.76892 2

"2862" 31 1.58504 2

"2863" 22 1.96383 2

"2864" 30 1.32025 2

"2865" 29 1.42322 2

"2866" 27 1.46492 2

"2867" 25 1.73889 2

"2868" 27 0.96352 2

"2869" 26 1.02052 2

"2870" 28 1.7744 2

"2871" 31 1.5501 2

"2872" 40 1.33377 2

"2873" 20 0 2

"2874" 20 0 2

"2875" 20 0.22985 2

"2876" 20 0.32179 2

"2877" 20 0.32179 2

"2878" 20 0.36776 2

"2879" 20 0.27582 2

"2880" 20 0.41373 2

"2881" 20 0.50567 2

"2882" 20 0.55164 2

"2883" 20 1.10328 2

"2884" 20 1.19522 2

"2885" 13 1.28716 2

"2886" 13 1.51701 2

"2887" 14 1.09901 2

"2888" 20 1.42507 2

"2889" 21 1.74686 2

"2890" 22 1.14925 2

"2891" 16 1.33313 2

"2892" 12 1.32886 2

"2893" 12 1.06834 2

"2894" 12 0.87343 2

"2895" 12 0.86489 2

"2896" 7 0.47073 2

"2897" 8 0.70058 2

"2898" 11 0.59334 2

"2899" 10 0.55164 2

"2900" 14 0.54737 2

"2901" 16 0.5167 2

"2902" 17 0.6307 2

"2903" 21 0.59761 2

"2904" 20 0.36776 2

"2905" 21 0.13791 2

"2906" 20 0 2

"2907" 20 0 2

"2908" 20 0 2

"2909" 20 0.18388 2

"2910" 20 0.36776 2

"2911" 20 0.32179 2

"2912" 20 0.22985 2

"2913" 20 0.36776 2

"2914" 20 0.4597 2

"2915" 16 0.87343 2

"2916" 16 1.05731 2

"2917" 16 1.19522 2

"2918" 20 1.33313 2

"2919" 21 1.56298 2

"2920" 22 1.3791 2

"2921" 35 1.24119 2

"2922" 36 2.16059 2

"2923" 37 2.12565 2

"2924" 48 1.79283 2

"2925" 51 2.02268 2

"2926" 55 1.65065 2

"2927" 56 1.46677 2

"2928" 54 1.56974 2

"2929" 70 1.32281 2

"2930" 60 1.0028 2

"2931" 72 0.77964 2

"2932" 68 0.59761 2

"2933" 75 0.32179 2

"2934" 20 0 2

"2935" 20 0 2

"2936" 20 0 2

"2937" 20 0 2

"2938" 20 0 2

"2939" 20 0.04597 2

"2940" 20 0.18388 2

"2941" 20 0.18388 2

"2942" 20 0.27582 2

"2943" 20 0.13791 2

"2944" 20 0.18388 2

"2945" 20 0 2

"2946" 20 0.09194 2

"2947" 20 0.18388 2

"2948" 10 0.9194 2

"2949" 11 0.96537 2

"2950" 15 0.82746 2

"2951" 16 1.05731 2

"2952" 18 1.10328 2

"2953" 19 1.10328 2

"2954" 19 1.33313 2

"2955" 24 1.42507 2

"2956" 21 1.80386 2

"2957" 26 1.9528 2

"2958" 24 1.10328 2

"2959" 30 1.05731 2

"2960" 28 1.0751 2

"2961" 19 0.83849 2

"2962" 20 0.78149 2

"2963" 20 1.0334 2

"2964" 21 1.29819 2

"2965" 23 0.82746 2

"2966" 20 0.87343 2

"2967" 20 0.04597 2

"2968" 27 0 2

"2969" 20 0 2

"2970" 20 0.04597 2

"2971" 20 0 2

"2972" 20 0 2

"2973" 20 0 2

"2974" 20 0 2

"2975" 20 0 2

"2976" 20 0 2

"2977" 20 0 2

"2978" 20 0 2

"2979" 20 0.18388 2

"2980" 20 0.32179 2

"2981" 20 0.27582 2

"2982" 20 0.36776 2

"2983" 20 0.50567 2

"2984" 20 0.73552 2

"2985" 20 0.64358 2

"2986" 10 0.78149 2

"2987" 10 1.14925 2

"2988" 11 1.10328 2

"2989" 12 1.28716 2

"2990" 13 1.69662 2

"2991" 15 1.74686 2

"2992" 17 2.07968 2

"2993" 30 0.9194 2

"2994" 32 1.47104 2

"2995" 35 1.8388 2

"2996" 35 2.07541 2

"2997" 42 2.53326 2

"2998" 53 2.25253 2

"2999" 57 2.9862 2

"3000" 71 3.59669 2

"3001" 70 2.77841 2

"3002" 75 3.45451 2

"3003" 80 3.0279 2

"3004" 75 3.30372 2

"3005" 73 3.25775 2

"3006" 77 3.10696 2

"3007" 89 2.80417 2

"3008" 87 3.10205 2

"3009" 89 3.50475 2

"3010" 80 3.0432 2

"3011" 83 3.52681 2

"3012" 94 4.42913 2

"3013" 93 3.69966 2

"3014" 98 3.37787 2

"3015" 92 2.89184 2

"3016" 94 3.30557 2

"3017" 91 3.84433 2

"3018" 104 3.99569 2

"3019" 98 2.64235 2

"3020" 87 2.40638 2

"3021" 74 1.33313 2

"3022" 71 1.28716 2

"3023" 72 1.51765 2

"3024" 20 0 2

"3025" 20 0 2

"3026" 20 0 2

"3027" 20 0 2

"3028" 20 0 2

"3029" 20 0 2

"3030" 20 0.04597 2

"3031" 20 0.09194 2

"3032" 20 0.18388 2

"3033" 20 0.22985 2

"3034" 20 0.22985 2

"3035" 20 0.18388 2

"3036" 20 0.32179 2

"3037" 20 0.22985 2

"3038" 20 0 2

"3039" 20 0.13791 2

"3040" 20 0.18388 2

"3041" 7 0.27582 2

"3042" 7 0.50567 2

"3043" 11 0.41373 2

"3044" 11 0.18388 2

"3045" 20 0 2

"3046" 20 0 2

"3047" 20 0 2

"3048" 20 0.09194 2

"3049" 20 0.13791 2

"3050" 20 0.22985 2

"3051" 20 0.09194 2

"3052" 20 0.36776 2

"3053" 20 0.32179 2

"3054" 20 0.27582 2

"3055" 20 0.22985 2

"3056" 20 0.41373 2

"3057" 20 0.55164 2

"3058" 18 0.64358 2

"3059" 17 0.9194 2

"3060" 24 0.87343 2

"3061" 22 1.23692 2

"3062" 29 0.82746 2

"3063" 29 0.82746 2

"3064" 27 1.10328 2

"3065" 30 1.00707 2

"3066" 30 1.11431 2

"3067" 32 1.01134 2

"3068" 28 1.05304 2

"3069" 28 1.10328 2

"3070" 30 0.9194 2

"3071" 26 0.77722 2

"3072" 29 0.96537 2

"3073" 30 1.53907 2

"3074" 26 1.35519 2

"3075" 24 0.80355 2

"3076" 26 1.60468 2

"3077" 24 1.39013 2

"3078" 20 1.0904 2

"3079" 25 1.0904 2

"3080" 24 0.82319 2

"3081" 22 0.77722 2

"3082" 25 1.35519 2

"3083" 23 1.06834 2

"3084" 23 1.24119 2

"3085" 22 1.3791 2

"3086" 23 0.88019 2

"3087" 23 1.11431 2

"3088" 25 0.82746 2

"3089" 19 0.73552 2

"3090" 18 0.87343 2

"3091" 20 0.78149 2

"3092" 22 0.73552 2

"3093" 21 0.68955 2

"3094" 20 0 2

"3095" 20 0 2

"3096" 20 0.09194 2

"3097" 20 0.18388 2

"3098" 20 0.18388 2

"3099" 20 0.22985 2

"3100" 20 0.4597 2

"3101" 15 0.32179 2

"3102" 11 0.50567 2

"3103" 10 0.87343 2

"3104" 8 0.9194 2

"3105" 12 0.73552 2

"3106" 14 1.24119 2

"3107" 14 1.28716 2

"3108" 18 1.28716 2

"3109" 18 1.19522 2

"3110" 24 1.37483 2

"3111" 25 1.10328 2

"3112" 30 1.65492 2

"3113" 25 1.33313 2

"3114" 29 1.3791 2

"3115" 30 2.03371 2

"3116" 34 1.88477 2

"3117" 31 1.84983 2

"3118" 30 1.65492 2

"3119" 23 1.10328 2

"3120" 25 1.61571 2

"3121" 23 0.9194 2

"3122" 21 0.96537 2

"3123" 27 1.48207 2

"3124" 27 1.39013 2

"3125" 33 1.76465 2

"3126" 36 1.94177 2

"3127" 39 1.53295 2

"3128" 37 1.52619 2

"3129" 38 1.0181 2

"3130" 40 1.2614 2

"3131" 24 0.87343 2

"3132" 1 0 2

"3133" 20 0 2

"3134" 20 0 2

"3135" 20 0.04597 2

"3136" 20 0.13791 2

"3137" 20 0.32179 2

"3138" 20 0.32179 2

"3139" 20 0.22985 2

"3140" 20 0.09194 2

"3141" 20 0.41373 2

"3142" 20 0.73552 2

"3143" 20 0.36776 2

"3144" 20 1.05731 2

"3145" 9 0.59761 2

"3146" 14 1.01134 2

"3147" 17 0.96537 2

"3148" 17 0.82746 2

"3149" 19 1.24119 2

"3150" 3 0 2

"3151" 20 0 2

"3152" 20 0 2

"3153" 20 0.18388 2

"3154" 20 0.36776 2

"3155" 20 0 2

"3156" 20 0.09194 2

"3157" 20 0.22985 2

"3158" 20 0.36776 2

"3159" 20 0.27582 2

"3160" 20 0.50567 2

"3161" 2 0.18388 2

"3162" 4 0.82746 2

"3163" 7 0.87343 2

"3164" 10 1.60895 2

"3165" 12 1.3791 2

"3166" 14 1.74686 2

"3167" 17 1.24119 2

"3168" 8 1.24119 2

"3169" 10 1.70089 2

"3170" 10 1.56298 2

"3171" 15 1.97671 2

"3172" 10 1.09474 2

"3173" 8 1.14925 2

"3174" 8 0.96537 2

"3175" 1 0.41373 2

"3176" 20 0 2

"3177" 20 0 2

"3178" 20 0.4597 2

"3179" 20 0.55164 2

"3180" 20 0.32179 2

"3181" 20 0.32179 2

"3182" 20 0.4597 2

"3183" 20 0.27582 2

"3184" 20 0.36776 2

"3185" 20 0.55164 2

"3186" 2 0.96537 2

"3187" 6 1.3791 2

"3188" 12 0.87343 2

"3189" 17 1.14925 2

"3190" 19 0.96537 2

"3191" 23 1.33313 2

"3192" 25 1.01134 2

"3193" 29 0.9764 2

"3194" 20 0.50567 2

"3195" 13 1.10328 2

"3196" 9 0.9764 2

"3197" 13 0.82746 2

"3198" 15 1.01134 2

"3199" 20 0.9764 2

"3200" 20 0 2

"3201" 20 0 2

"3202" 20 0 2

"3203" 20 0.04597 2

"3204" 20 0.22985 2

"3205" 20 0.36776 2

"3206" 20 0.36776 2

"3207" 20 0.27582 2

"3208" 20 0.36776 2

"3209" 20 0.32179 2

"3210" 20 0.4597 2

"3211" 2 1.19522 2

"3212" 6 1.10328 2

"3213" 13 0.87343 2

"3214" 20 1.28716 2

"3215" 23 1.51701 2

"3216" 28 1.33313 2

"3217" 25 1.33313 2

"3218" 30 1.55444 2

"3219" 34 1.65492 2

"3220" 35 2.16735 2

"3221" 41 1.97671 2

"3222" 38 1.10328 2

"3223" 30 0.9194 2
